# Potential of Marine Sponge Metabolites against Prions: Bromotyrosine Derivatives, a Family of Interest

**DOI:** 10.3390/md22100456

**Published:** 2024-10-04

**Authors:** Maha Sinane, Colin Grunberger, Lucile Gentile, Céline Moriou, Victorien Chaker, Pierre Coutrot, Alain Guenneguez, Marie-Aude Poullaouec, Solène Connan, Valérie Stiger-Pouvreau, Mayalen Zubia, Yannick Fleury, Stéphane Cérantola, Nelly Kervarec, Ali Al-Mourabit, Sylvain Petek, Cécile Voisset

**Affiliations:** 1 Univ Brest, Inserm, EFS, UMR 1078, GGB, School of Medicine, F-29200 Brest, France; maha.sinane@univ-brest.fr (M.S.); lucile.gentile@univ-brest.fr (L.G.); vico.chaker@laposte.net (V.C.); pcoutrot@outlook.fr (P.C.); 2 IRD, CNRS, Ifremer, Univ Brest, LEMAR, IUEM, F-29280 Plouzane, France; colin.grunberger@ird.fr (C.G.); alain.guenneguez@ird.fr (A.G.); marie-aude.poullaouec@univ-brest.fr (M.-A.P.); solene.connan@univ-brest.fr (S.C.); valerie.stiger@univ-brest.fr (V.S.-P.); 3 CNRS, Institut de Chimie des Substances Naturelles, Université Paris-Saclay, F-91190 Gif-sur-Yvette, France; celine.moriou@cnrs.fr (C.M.); ali.almourabit@cnrs.fr (A.A.-M.); 4 UPF, Ifremer, ILM, IRD, UMR 241 SECOPOL, BP6570, 98702 Faa’a, Tahiti, French Polynesia; mayalen.zubia@upf.pf; 5 Univ Brest, Univ Bretagne Sud, CNRS, LBCM, EMR 6076, F-29000 Quimper, France; yannick.fleury@univ-brest.fr; 6 Univ Brest, Plateforme RMN-RPE, F-29238 Brest, France; stephane.cerantola@univ-brest.fr; 7 Univ Brest, Plateforme Spectrométrie de Masse, F-29238 Brest, France; nelly.kervarec@univ-brest.fr; 8 Univ Brest, Inserm, UMR 1101, LaTIM, School of Medicine, F-29200 Brest, France

**Keywords:** marine sponges, prion diseases, yeast-based screening, PrP^Sc^, ER stress, bromotyrosine derivatives, Verongiida, *Suberea laboutei*

## Abstract

The screening of 166 extracts from tropical marine organisms (invertebrates, macroalgae) and 3 cyclolipopeptides from microorganisms against yeast prions highlighted the potential of Verongiida sponges to prevent the propagation of prions. We isolated the known compounds purealidin Q (**1**), aplysamine-2 (**2**), pseudoceratinine A (**3**), aerophobin-2 (**4**), aplysamine-1 (**5**), and pseudoceratinine B (**6**) for the first time from the Wallisian sponge *Suberea laboutei*. We then tested compounds **1**–**6** and sixteen other bromotyrosine and bromophenol derivatives previously isolated from Verongiida sponges against yeast prions, demonstrating the potential of **1**–**3**, **5**, **6**, aplyzanzine C (**7**), purealidin A (**10**), psammaplysenes D (**11**) and F (**12**), anomoian F (**14**), and N,N-dimethyldibromotyramine (**15**). Following biological tests on mammalian cells, we report here the identification of the hitherto unknown ability of the six bromotyrosine derivatives **1**, **2**, **5**, **7**, **11**, and **14** of marine origin to reduce the spread of the PrP^Sc^ prion and the ability of compounds **1** and **2** to reduce endoplasmic reticulum stress. These two biological activities of these bromotyrosine derivatives are, to our knowledge, described here for the first time, offering a new therapeutic perspective for patients suffering from prion diseases that are presently untreatable and consequently fatal.

## 1. Introduction

Unlike traditional infectious agents such as viruses, bacteria, parasites, or fungi, prions are unconventional infectious agents composed solely of a protein with an alternative three-dimensional conformation, and containing no genetic material such as DNA or RNA [1,2,3,4].

The physiological form of the prion protein (PrP^C^) is found in healthy cells, particularly in the nervous system. However, in prion diseases, PrP^C^ undergoes conformational changes, folding into a pathogenic conformation known as PrP^Sc^. The unique characteristic of PrP^Sc^ is its ability to act as a template, inducing PrP^C^ to misfold into the PrP^Sc^ pathological conformation and aggregate. PrP^Sc^ capacity for templating and conversion allows it to self-propagate and spread within an organism as well as between individuals without involving traditional genetic material [5,6]. The accumulation of PrP^Sc^ oligomers and aggregates, which disrupts cellular function over time, induces neuronal loss, and vacuolation, ultimately leading to neurodegenerative disorders. PrP^Sc^ propagation contributes to the spongiform appearance of brain architecture observed in people affected by prions, which is a signature of prion diseases.

Prion diseases, also called transmissible spongiform encephalopathies (TSEs), are a group of rare and fatal neurological disorders that affect both humans and animals. Human prion diseases (Creutzfeldt–Jakob Disease (CJD), variant Creutzfeldt–Jakob Disease (vCJD), Gerstmann–Sträussler–Scheinker Disease (GSS) and Fatal Familial Insomnia (FFI) [7]) are rare, with annual incidence between 1.5 and 2 cases per million worldwide according to the National Public Health Agency. They can occur in sporadic forms, through genetic mutations (inherited forms) or via exposure to prion-contaminated tissues or products. The misfolded prion protein is remarkably resilient and can withstand standard decontamination methods, making these pathogens difficult to control.

The specific features of prions make prion diseases particularly challenging to target using traditional therapeutic approaches. While there are currently no approved cures for prion diseases [7,8], our research group [9,10,11,12] and others [7,13] have been actively exploring different approaches to develop potential therapies that could target prion proteins or the underlying mechanisms involved in prion propagation and aggregation, which are still poorly understood. A large variety of natural compounds derived from plants [14], animals [15,16,17], and other sources has been investigated for their ability to inhibit prion propagation, reduce aggregation of misfolded prion proteins, or mitigate the effects of prion-related neurodegeneration [13]. The anti-prion properties of some natural products [18], including curcumin (turmeric spice) [19], resveratrol (grapes and certain berries), polyphenol epigallocatechin gallate (EGCG, green tea), and flavonoids (fruits, vegetables, and other plants) have thus been described. It is important to emphasize that while these natural products are promising in vitro and in animal models, their effectiveness and safety in treating prion diseases in humans are not yet established.

The chemodiversity of marine organisms offers huge potential as a reservoir for the development of new therapies [20,21,22,23,24]. As a part of our ongoing program on chemical exploration of natural products for anti-prion purposes, several libraries of marine invertebrates and macro-algal extracts from French overseas territories (French Polynesia, Wallis and Futuna) have been screened, as have cyclolipopeptides from *Pseudoaltromonas rhizosphaerae*. These preliminary screenings particularly highlighted the interest of sponges. Indeed, marine sponges are known to produce a wide range of bioactive compounds [25] and two Australian species, *Suberea ianthelliformis* and *Lamellodysidea* cf. *chlorea,* have previously been studied for their potential activity against yeast prions [15,17]. We therefore continued to explore a panel of marine sponges for further investigation. Of the 131 Polynesian sponge extracts tested, two from *Aplysinellidae* sponges and one from a *Pseudoceratinidae* sponge were active against both [*PSI*+] and [URE3] yeast prions. Based on these results, the chemical composition of the as-yet unstudied *Suberea laboutei* from Wallis Island was examined in order to isolate and identify its anti-prion metabolites. Six bioactive bromotyrosine derivatives were thus identified.

In the course of our previous studies, several brominated tyrosine analogs with biological and ecological activities were discovered in the Oceanian marine sponges *Suberea ianthelliformis* [26], *Suberea clavata* [27], and *Pseudoceratina* sp. [28]. Bromotyrosine derivatives have attracted interest in the field of natural products research due to their wide range of biological activities [29,30], including notably antimalarial [31,32], biocidal antifouling [28,33,34,35], antiplasmodial [36], antimycobacterial [37,38], antiviral [39,40] and antifungal activities [41,42], cytotoxicity [43,44], specific histamine H-3 receptor antagonist capacities [45], and inhibition of enzymes [26,27,46]. Several bromotyrosine compounds were also shown to be able to inhibit cancer proliferation, invasion, and migration [26,47,48,49].

In the context of prion diseases, some studies have explored the potential of bromotyrosine metabolites as inhibitors of prion protein aggregation and propagation. In particular, aplysamine-1, aplysamine-2, and purealidin Q, isolated from the Aplysinellidae sponge *Suberea ianthelliformis* have already been described as active against [*PSI*+] and [URE3] yeast prions [15]. Psammaplysene A has recently been shown to reduce prion levels in prion-infected cells and cerebellar organotypic slices and to alleviate locomotor deficits in prion-infected *Drosophila* expressing ovine PrP^C^ [50]. Here, we screened extracts and metabolites from tropical marine organisms (invertebrates, macroalgae) and microorganisms for their capacity to reduce prion propagation, using both [*PSI*+] and [URE3] yeast prions, as well as the PrP^Sc^ mammalian prion, and to alleviate endoplasmic reticulum stress.

## 2. Results and Discussion

### 2.1. Primary Screening of Various Marine Organism Extracts against [PSI+] and [URE3] Yeast Prions

To explore the potential of marine organism chemodiversity, we studied organic extracts (Table S1) of 141 tropical invertebrates from French Polynesia [51,52,53,54] and Wallis and Futuna [55], 25 macroalgae from French Polynesia [56,57], and 3 cyclolipopeptides known as alterins produced by *Pseudoalteromonas* strains isolated in Brittany [58].

To select the extracts containing molecules with anti-prion activity, we used a yeast-based screening method [59], whose effectiveness has already been widely proven [10,11,12]. This two-step assay started with the screening of crude extracts against the [*PSI*+] yeast prion (primary screening). Extracts able to cure the [*PSI*+] prion were then assayed against a second yeast prion [URE3] (secondary screening). Extracts displaying an anti-prion activity against both [*PSI*+] and [URE3] yeast prions were then selected for further analysis. Briefly, [*PSI*+] or [URE3] yeast cells were spread on Petri dishes containing a solid agar-based rich medium. Extracts were loaded onto individual filters placed on the agar surface and the dishes were further incubated for seven to ten days at 25 °C. The yeast strains form white colonies when they contain [*PSI*+] or [URE3] prions, whereas they form red colonies when the [*PSI*+] or [URE3] prions have been eliminated. Upon the addition of an extract to a filter, its anti-prion activity was thus detected when a halo of red colonies appeared around the filter. Screening scores were attributed as shown in Table 1.

**Figure 1:**
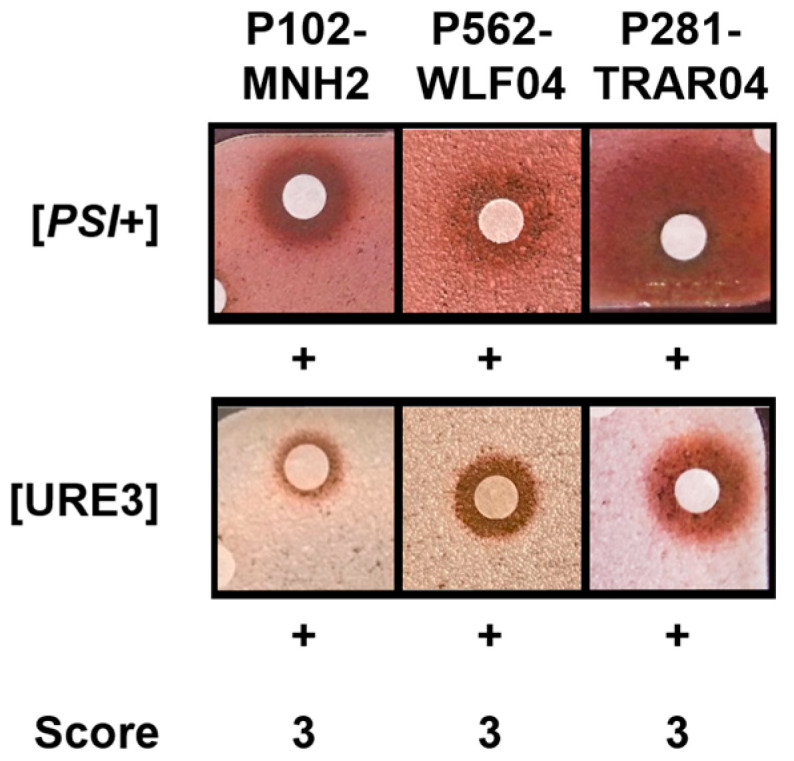
Sponge extracts displaying activity against [*PSI*+] and [URE3] yeast prions. Yeast cells were spread on a rich agar medium and small sterile filters were then placed on the agar surface, and 2 µL of each 10 mg/mL sponge extract was applied to the individual filters. A red halo around the filter on which an extract was loaded indicates that the extract is active against [*PSI*+] or [URE3] prions, whereas colonies that remain white indicate inactive extracts. Screening scores were attributed according to Table 1.

The 169 samples were first assayed for their capacity to cure the [*PSI*+] yeast prion (Table S1). None of the algae, tunicates, or microorganisms tested displayed activity against [*PSI*+]. From the sponge extracts displaying anti-prion activity against [*PSI*+], two extracts from *Aplysinellidae* sponges and one extract from a *Pseudoceratinidae* sponge were active against both [*PSI*+] and [URE3] yeast prions (screenings scored 3, Table 2, Figure 1 and Table S1).

### 2.2. Bioguided Isolation and Identification of Bioactive Compounds from Suberea laboutei

**Figure 2:**
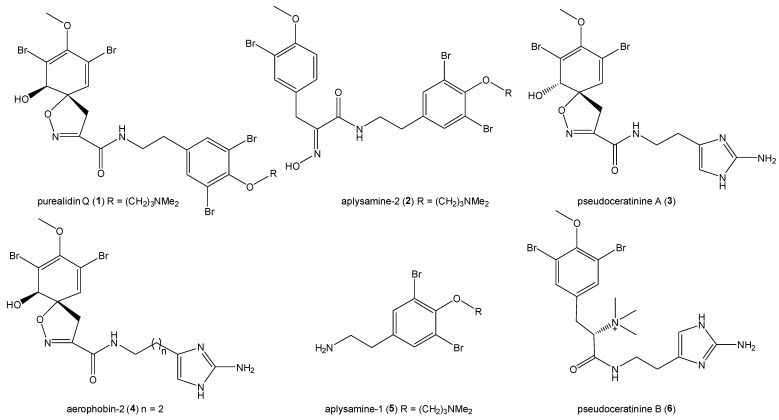
Structures of the isolated compounds **1**–**6** from *Suberea laboutei*.

The freeze-dried sponge *Suberea laboutei* was extracted with a mixture of CH_2_Cl_2_/MeOH (1/1). The resulting crude extract was partitioned between CH_2_Cl_2_ and water to give an organic extract C, a precipitate P, and an aqueous layer. The aqueous layer was further partitioned against n-butanol to furnish a crude butanolic extract B. Bioguided fractionation of the extracts C and B, using silica gel normal-phase and/or reversed-phase chromatography led to the isolation of the known compounds: purealidin Q (**1**) [46] and aplysamine-2 (**2**) [60] from extract C, and pseudoceratinine A (**3**) [61], aerophobin-2 (**4**) [62], aplysamine-1(**5**) [60], and pseudoceratinine B (**6**) [61] from extract B (Figure 2), all of which were identified after extensive spectroscopic analyses and comparison with data from the literature (see Supporting information).

To the best of our knowledge, this is the first report of the presence of metabolites **1**–**6** in *Suberea laboutei* species. The genus *Suberea*, with 78 known compounds [63], is particularly prolific in bromophenol and bromotyrosine derivatives, with 69 metabolites registered to date. Purealidin Q (**1**), aplysamine-1 (**5**), and aplysamine-2 (**2**) have also been identified in *Suberea ianthelliformis* [15,64], and compounds **1**–**6** were previously isolated from various Verongiida sponges of the genera *Pseudoceratina* (ex-*Psammaplysilla)* [46,61,65,66,67,68], *Aplysina* (ex-*Verongia*) [60], *Aiolochroia* [62], and *Aplysinella* [69].

**Figure 3:**
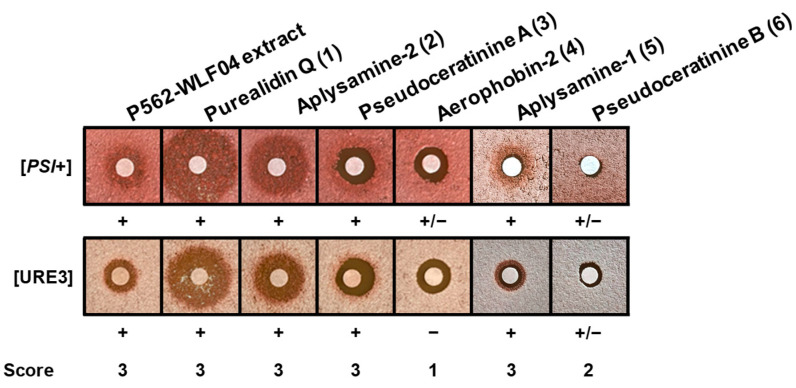
Anti-prion activity of compounds **1**–**6** against [*PSI*+] and [URE3] yeast prions as described in Figure 1. Screening scores were attributed according to Table 1. Molecules **1**, **2**, **3**, **5**, and **6** are active against both yeast prions.

When these six molecules (**1**–**6**) were first tested against [*PSI*+] and [URE3] yeast prions, purealidin Q (**1**), aplysamine-2 (**2**), pseudoceratinine A (**3**), aplysamine-1 (**5**), and pseudoceratinine B (**6**) were able to cure both [*PSI*+] and [URE3] prions (scored 2 or 3, Figure 3), whereas aerophobin-2 (**4**) only cured [*PSI*+] (scored 1, Figure 3).

**Figure 4:**
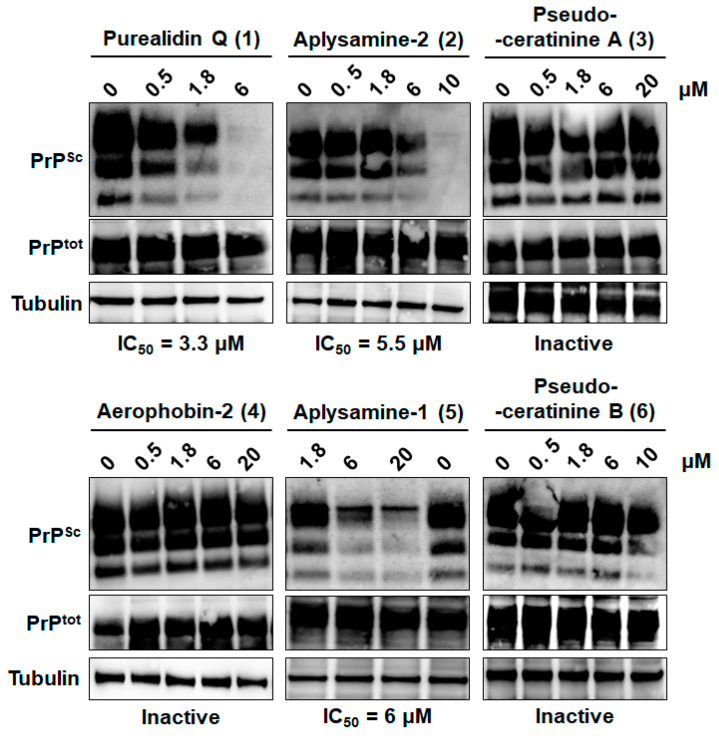
Anti-PrP^Sc^ activity of compounds **1**–**6** on prion-infected MovS6 cells. MovS6 cells were treated with the indicated ranges of concentrations of the molecules, and absolute ethanol was used as a negative control. After 6 days of culture, cell lysates were digested by proteinase K to reveal PrP^Sc^ (top panels) or untreated to reveal PrP^C^ (middle panels) or the loading control Tubulin (bottom panels). Proteins were separated in 10% Bis-Tris polyacrylamide gels and revealed using anti-PrP (Sha31) or anti-tubulin-specific antibodies. The blots shown are representative of two to three independent experiments that all produced similar results. Purealidin Q (**1**), aplysamine-2 (**2**) and aplysamine-1 (**5**) were able to reduce PrP^Sc^ propagation, whereas pseudoceratinine A (**3**), aerophobin-2 (**4**), and pseudoceratinine-B (**6**) were not.

Based on these results, molecules **1**, **2**, **3**, **5**, and **6**, which were shown to be active against both yeast prions, were further tested for their ability to decrease the propagation of the pathogenic conformation of the mammalian prion protein PrP^Sc^ in a cell-based assay using chronically prion-infected MovS6 cells [70]. MovS6 is a murine peripheral neuroglial cell line expressing the ovine PrP protein under the control of its endogenous gene promoter. These cells are chronically infected by the 127S sheep scrapie prion [70,71] and allow the rapid testing of molecules against mammalian prions. After 6 days of treatment of prion-infected MovS6 cells with bromotyrosine derivatives **1**–**6**, PrP^Sc^ was detected on the basis of its proteinase K (PK) resistance. For this purpose, a fraction of cell lysates was treated by PK to discriminate PrP^Sc^ proteins, which are partially resistant to PK treatment, from total PrP proteins (PrP^tot^). PK-treated and -untreated cell lysates were separated by SDS-PAGE and immunostained using specific anti-PrP antibodies. Purealidin Q (**1**), aplysamine-2 (**2**), and aplysamine-1 (**5**) were able to reduce PrP^Sc^ propagation with IC_50_ values of 3.3, 5.5, and 6 µM, respectively (Figure 4).

Pseudoceratinine A (**3**) showed no activity against PrP^Sc^ up to 20 µM. Pseudoceratinine B (**6**) was also inactive up to 10 µM and was cytotoxic at concentrations above 10 µM. Aerophobin-2 (**4**), which was used as a negative control as it was inactive against both [*PSI*+] and [URE3] yeast prions (Figure 3), was not active up to 20 µM either (Figure 4). Purealidin Q (**1**), aplysamine-2 (**2**), and aplysamine-1 (**5**) have previously been described as active against both [*PSI*+] and [URE3] yeast prions [15]. Here, we confirm these three bromotyrosine derivatives are potent anti-prion compounds as we found that they are also able to reduce the propagation of the mammalian prion PrP^Sc^.

### 2.3. Anti-Prion Activity of Bromotyrosine Derivatives from Sponges of the Order Verongiida

**Figure 5:**
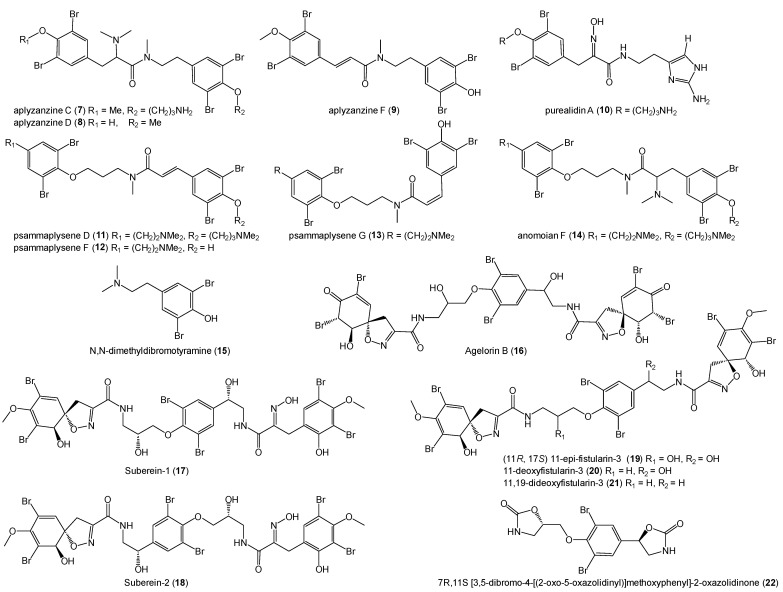
Structures of the tested bromotyrosine and bromophenol derivatives **7**–**22**.

We identified two bromotyrosine derivatives with anti-prion activity among the six molecules purified from the *Suberea laboutei* extract (P562-WLF04). Concerning the samples of *Suberea ianthelliformis* (P102-MNH2) and *Pseudoceratina* sp. (2081) (P281-TRAR04), which also showed anti-prion activity (Table 2), their previously isolated bromotyrosine and bromophenol derivatives were directly selected as candidate molecules for screening. To diversify the panel of structures, compounds extracted from *Suberea clavata* collected in the Solomon Islands were added to the study. The molecules tested included the aplyzanzines C (**7**), D (**8**), and F (**9**) and purealidin A (**10**) from *Pseudoceratina* sp. (2081) [28]; the psammaplysenes D (**11**), F (**12**), and G (**13**), anomoian F (**14**), and N,N-dimethyldibromotyramine (**15**) from *Suberea ianthelliformis* [26]; agelorin B (**16**), subereins-1 and subereins-2 (**17**, **18**), (11*R*, 17*S*) 11-*epi*-fistularin-3 (**19**), 11-deoxyfistularin-3 (**20**), 11,19-dideoxyfistularin-3 (**21**), and 7*R*,11*S* [3,5-dibromo-4-[(2-oxo-5-oxazolidinyl)]methoxyphenyl]-2-oxazolidinone (**22**) from *Suberea clavata* [27,72] (Figure 5).

**Figure 6:**
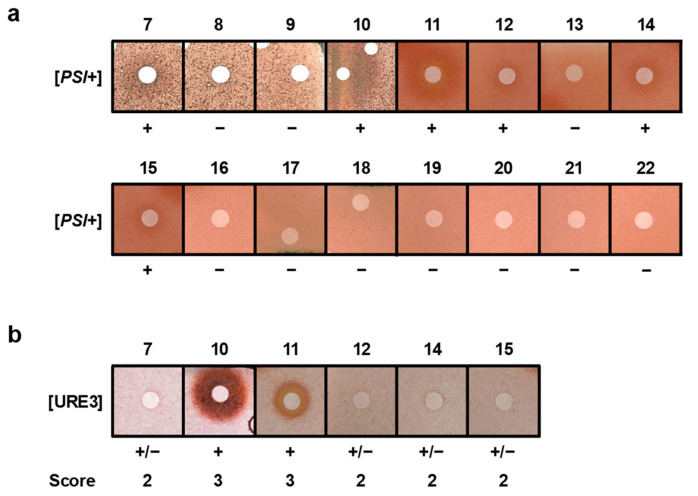
Activity of bromotyrosine derivatives **7**–**22** against [*PSI*+] (**a**) and [URE3] (**b**) yeast prions, as described in Figure 1. Screening scores were attributed according to Table 1. Compounds **7**, **10**, **11**, **12**, **14**, and **15** were active against both [*PSI*+] and [URE3] yeast prions.

We tested the capacity of this library of 16 additional bromotyrosine metabolites (Figure 5) against [*PSI*+] and found that six of them (**7**, **10**, **11**, **12**, **14**, and **15**, Figure 6a) were active. Moreover, these metabolites were also active against [URE3] (Figure 6b). Compounds **8**, **9**, **13**, and **16**–**22**, however, were not active against [*PSI*+] and were therefore not tested against [URE3] (Figure 6a).

**Figure 7:**
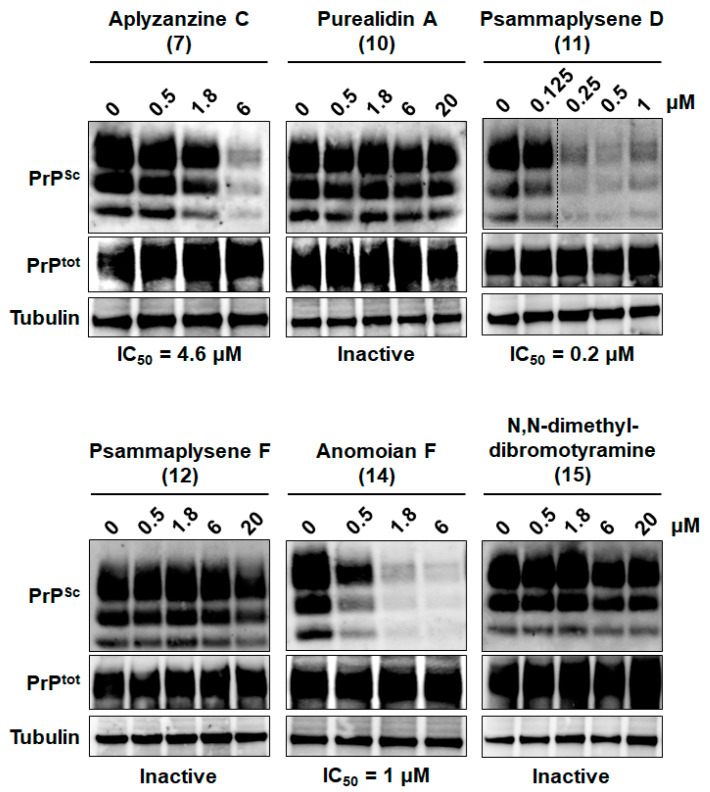
Activity of bromotyrosine derivatives **7**, **10**, **11**, **12**, **14**, and **15** against the PrP^Sc^ prion and on prion-infected MovS6 cells, as described in Figure 4. The blots shown are representative of two to three independent experiments that all produced similar results. Aplyzanzine C (**7**), psammaplysene D (**11**), and anomoian F (**14**) were able to reduce PrP^Sc^ propagation, whereas purealidin A (**10**), psammaplysene F (**12**) and N,N-dimethyl-dibromotyramine (**15**) were not.

When tested against PrP^Sc^, aplyzanzine C (**7**), psammaplysene D (**11**), and anomoian F (**14**) were able to reduce PrP^Sc^ propagation in a dose-dependent manner, with IC_50_ scores of 4.6, 0.2, and 1 µM, respectively (Figure 7). Purealidin A (**10**), psammaplysene F (**12**), and N,N-dimethyl-dibromotyramine (**15**) were not able to reduce PrP^Sc^ propagation below 20 µM (Figure 7). To our knowledge, this is the first report of the anti-prion activity of aplyzanzine C, psammaplysene D, and anomoian F. Recently, the anti-prion activity of psammaplysene A, a close analog of psammaplysene D, was found indirectly through an arrayed genome-wide RNA interference (RNAi) screening aimed at identifying cellular host factors that can modify prion propagation [50]. This screening identified the heterogeneous nuclear ribonucleoprotein Hnrnpk as a prominent limiter of prion propagation. Psammaplysene A, which binds Hnrnpk [73], has been further shown to reduce PrP^Sc^ prion levels in prion-infected cells and cerebellar organotypic slices and to alleviate locomotor deficits in prion-infected *Drosophila* expressing ovine PrP^C^ [50]. Like psammaplysene D, psammaplysene A was also active against [*PSI*+] and [URE3] yeast prions (Figure S1a) and displayed similar activity against the PrP^Sc^ prion (IC_50_ = 0.3 µM, Figure S1b).

### 2.4. Endoplasmic Reticulum Stress Reduction Activity of Bromotyrosine Derivatives

**Figure 8:**
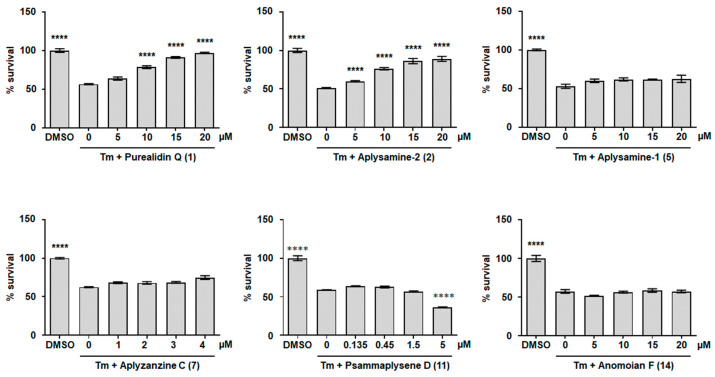
Cytoprotective activity of bromotyrosine derivatives purealidin Q (**1**), aplysamine-2 (**2**), aplysamine-1 (**5**), aplyzanzine C (**7**), psammaplysene D (**11**), and anomoian F (**14**) against ER stress. CHO-K1 cells were ER-stressed using 0.45 µg/mL tunicamycin (Tm) in the presence of the indicated concentrations of the test molecules or DMSO as a negative control. After 24 h of treatment, cell viability was measured by the quantification of the number of live cells using the WST-8 tetrazolium salt. Values are shown relative to the DMSO-treated cells, which was set at a value of 100%. A representative assay including three technical repeats is shown with SD error bars, and each experiment was performed at least three times with similar results. Bar height represents the mean relative to DMSO-treated cells. **** *p* < 0.0001 one-way ANOVA compared with Tm-treated cells, followed by Dunnett’s test.

Prion diseases belong to a group of diseases called protein misfolding diseases, whose common molecular feature is the pathologic aggregation of specific proteins. Protein aggregation leads to stress on the endoplasmic reticulum (ER), which, when too high or sustained, triggers the unfolded protein response (UPR), an adaptive response whose purpose is to reduce ER stress. In case of failure to reduce ER stress, the cell then becomes engaged in an apoptotic pathway [74,75]. Guanabenz, a drug used to treat hypertension, has been shown to exhibit both anti-prion activity [9,11] and an ability to reduce UPR-induced cell death [76,77]. We thus sought to determine whether bromotyrosine derivatives displaying anti-prion activity (molecules **1**, **2**, **5**, **6**, **7**, **11**, and **14**) were also able to reduce ER stress. For this purpose, we used the reference cell line CHO-K1 to study the capacity of these bromotyrosine derivatives to reduce UPR-induced cell death [78]. CHO-K1 cells were treated with tunicamycin (Tm), an antibiotic that induces ER stress by inhibiting protein N-glycosylation [79], which triggers UPR and cell death. Using 0.45 µg/mL of Tm, the cell viability level falls to 50 to 60% compared with untreated cells. In the presence of purealidin Q (**1**) and aplysamine-2 (**2**), we found a dose-dependent improvement in cell survival, indicating that these two molecules protect CHO-K1 cells from the ER stress induced by Tm (Figure 8). Aplysamine-1 (**5**), aplyzanzine C (**7**), psammaplysene D (**11**), and anomoian F (**14**) had no cytoprotective effect on ER-stressed CHO-K1 cells (Figure 8). Aplyzanzine C (**7**) and psammaplysene D (**11**) were tested at concentrations below 5 µM since they were cytotoxic at concentrations higher than or equal to 5 µM (Figure S2). The anti-prion molecule psammaplysene A [50] was not able to protect CHO-K1 cells from ER stress (Figure S1c) at concentrations below 2 µM since it was toxic at concentrations higher than 2 µM (Figure S1d).

**Figure 9:**
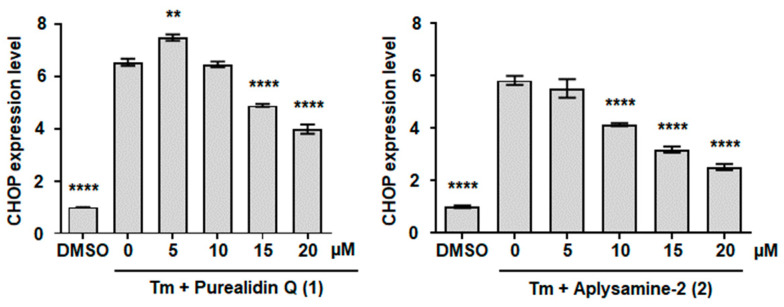
The effect of purealidin Q (**1**) and aplysamine-2 (**2**) on CHOP expression level. After 24 h treatment of CHO-K1 cells with Tm and test molecules as described in Figure 8, luciferase activity was measured. A representative assay including three technical repeats is shown with SD error bars, and each experiment was performed at least three times with similar results. Bar height represents the mean relative to DMSO-treated cells. ** *p* < 0.01, **** *p* < 0.0001 one-way ANOVA compared with Tm-treated cells, followed by Dunnett’s test.

The pro-apoptotic factor CHOP is a marker of UPR induction [74,75]. The CHO-K1 cell line contains the *luciferase* reporter gene placed under the control of the *CHOP* promotor (p*CHOP*::*luciferase*, [78]). Thus, the level of luciferase expressed in these cells is proportional to the level of CHOP induction. In order to determine whether the cytoprotective molecules **1**, **2**, and **5** act upstream or downstream of the induction of CHOP-mediated apoptosis, the luciferase expression level was monitored in cells treated with Tm and the bromotyrosine derivatives. Tm treatment induces UPR, resulting in a strong increase in p*CHOP*::*luciferase* expression level. Purealidine Q (**1**) and aplysamine-2 (**2**) were able to reduce p*CHOP*::*luciferase* expression (Figure 9) with no toxic effect (Figure S2), indicating that these molecules act upstream of the pro-apoptotic factor CHOP. To our knowledge, this is the first report of the capacity of bromotyrosine derivatives to protect cells from ER stress and to inhibit UPR.

## 3. Materials and Methods

### 3.1. General Experimental Procedure

The NMR spectra were recorded on a Bruker 500 MHz instrument Avance III HD500, equipped with a TCI 5 mm inverse cryoprobe. The chemical shifts are reported in ppm relative to the residual signal solvent (MeOH-*d*_4_: *δ*_H_ 3.31; *δ*_C_ 49.15). High-resolution mass spectra were obtained with a Q-TOF mass spectrometer (Synapt XS, Waters Corporation, Milford, NY, USA) at the UBO platform or on a Q-Exactive spectrometer (Thermo Fisher Scientific, Waltham, MA, USA) at the CRMPO platform, both using a direct electrospray infusion (ESI) of the purified compounds diluted in AcN. MPLC chromatography was performed using a glass column filled with silica (Silica 60, 40–60 µm, Macherey-Nagel, Dueren, Germany) or C18 (60 C18 ZEOprep, 40–63 µm, Zeochem AG, Rüti, Switzerland). Analytical and semi-preparative HPLC purifications were performed on an HPLC system [UltiMate 3000 pump, sampler, column oven, photodiode array detector, fraction collector (Thermo Fisher Scientific) and a Sedex 85 evaporative light scattering detector (Sedere, Alfortville, France)], equipped with Kinetex EVO C18 (2.1 × 150 mm, 2.6 µm), (4.6 × 150, 5 µm), (10 × 150 mm, 5 μm) columns (Phenomenex, Torrance, CA, USA). All chemicals and solvents were purchased from Sigma-Aldrich (Saint-Louis, MO, USA), or Honeywell (Charlotte, NC, USA).

### 3.2. Biological Material

Tropical invertebrates were collected by hand using SCUBA between 6 and 50 m depth in French Polynesia and Wallis and Futuna, during the sampling cruises BSMPF-1 [51], Tuam’2011 [52], Tuhaa Pae 2013 [54], Tahiti iti [53,80,81], and Wallis 2018 [55] aboard the R/V Alis (Table S1). Samples were deep-frozen on board until work-up. They were then freeze-dried, ground, and extracted, as previously described [82]. The sponge *Suberea laboutei* (P562-WLF04) was collected in the Wallis Island lagoon (13°20.308′ S, 176°10.392′ W) at a depth of 15 meters in July 2018. A voucher sample was deposited at the Queensland Museum (Brisbane, Australia) under the access number G339007 and was identified by Dr Merrick Ekins. Macroalgae from French Polynesia were collected by hand while snorkeling at depths of 1 to 3 meters in Tahiti, Moorea, and Tubuai (Table S1) [56]. Alterins were purified and characterized from the cell-free supernatant of *Pseudoaltromonas rhizosphaerae* h*Cg*-6 as previously described [58].

### 3.3. Extraction and Isolation

The freeze-dried sponge sample of *Suberea laboutei* (185.7 g) was extracted by maceration at room temperature with 3 × 500 mL of a mixture of CH_2_Cl_2_/MeOH (1:1) for 18 h and 2 × 2 h, respectively, and then by ultrasound-assisted extraction (UAE) with 2 × 250 mL of the same solvent for 2 × 30 min. The filtered extracts were combined and dried under reduced pressure, which produced a brown residue (13.43 g) that was partitioned between CH_2_Cl_2_ and H_2_O. A dry organic extract C (1.9 g) and a precipitate noted P (5.2 g) were obtained after evaporation. The aqueous layer was partitioned with n-BuOH to give, after drying, the crude extract B (2.1 g).

The organic extract C (1.89 g) was submitted to normal-phase silica-gel flash chromatography with a gradient from 1:0 to 0:1 of a mixture of cyclohexane:AcOEt, then a gradient from 1:0 to 1:1 of a mixture of CH_2_Cl_2_:MeOH to give fractions C-F1 to F33. Only C-F32 (15.3 mg, 20% MeOH) and C-F33 (292.4 mg, 50% MeOH) were assayed against yeast prions and showed anti-prion activities against [*PSI+*] and [URE3]. Fraction C-F32 (11.25 mg) was purified by semi-preparative reversed-phase HPLC (column: Phenomenex Kinetex EVO C18, 10 × 150 mm, 5 μm, H_2_O + 0.1% formic acid (FA)/CH_3_CN + 0.1% FA) to give purealidin Q (1.9 mg, **1**) and aplysamine-2 (1.1 mg, **2**).

The butanolic extract B (1.61 g) was fractionated by reversed-phase C18 flash chromatography with a gradient from 1:0 to 0:1 of a mixture of H_2_O/MeOH then EtOH, CH_3_CN, AcOEt, and finally CH_2_Cl_2_ to give 40 fractions B-F1 to F40. Numerous fractions showed anti-prion activities against [*PSI*+], but only B-F1 (57 mg, 5%MeOH), B-F3 (50.7 mg, 20% MeOH), B-F21 (21 mg), B-F22 (18.4 mg), and B-F23 (15.3 mg) with 100% MeOH, were active against both [*PSI*+] and [URE3] yeast prions. B-F1 (57 mg) and B-F3 (20 mg) were purified by semi-preparative reversed-phase HPLC (column: Phenomenex Kinetex EVO C18, 10 × 150 mm, 5 μm, H_2_O + 0.1% FA/CH_3_CN + 0.1% FA) to give pseudoceratinine A (4.2 mg, **3**) and aerophobin-2 (3.3 mg, **4**) from B-F3, aplysamine-1 (1.9 mg, **5**), and pseudoceratinine B (2.6 mg, **6**) from B-F1.

### 3.4. Yeast-Based Anti-Prion Screening Assay

The yeast-based screening assay was performed as previously described [59]. The *Saccharomyces cerevisiae* yeast strains used in this study were STRg6 (74-D694, Mata, *erg6*::*TRP1*, *ade1−14*, *trp1−289*, *his3*Δ200, *ura3−52*, *leu2−3,112*), containing the strong [*PSI*+] prion strain, and SB34 (Mata, *erg6*::*TRP1*, *dal5*::*ADE2*, *ade2−1*, *trp1−1*, *leu2−3*,*112*, *his3−11, 15*, *ura2*::*HIS3*), containing the [URE3] prion [59]. Yeasts containing the [*PSI+*] or [URE3] prions form white colonies, while yeasts without [*PSI+*] or [URE3] prions form red colonies [83]. The screening assay for the identification of compounds active against yeast [*PSI+*] and [URE3] prions takes advantage of this colorimetric reporter system. Aliquots of 360 μL (STRg6 strain) or 170 μL (SB34 strain) of 0.55 OD_600_ exponentially growing cultures were spread using sterile glass beads on square (12 cm × 12 cm) Petri plates containing YPD-rich medium supplemented with guanidine hydrochloride (GuHCl, Sigma Aldrich, Saint-Louis, MO, USA): 200 µM for [*PSI+*] and 800 μM for [URE3]. Small sterile filters (Thermo Fisher Scientific) were then placed on the agar surface, and 2 µL of each extract, fraction, or purified molecule was applied individually to each filter. For negative and positive controls, 5 µL DMSO (PAN Biotech, Aidenbach, Germany) and 2 µL GuHCl 1 M were used, respectively. The screening plates were incubated for seven to ten days at 25 °C until the appearance of the red coloration around the filter on which the positive control had been loaded. Colonies formed a red halo if the product spotted on a filter had anti-prion activity, but remained white around the filter if the molecule was inactive against yeast prions.

### 3.5. PrP^Sc^ Clearance Assay

PrP^Sc^ clearance assays in MovS6 cells were performed as previously described [10,11,12]. Briefly, MovS6 cells were seeded in 6-well plates (Starlab, Orsay, France) at a density of 5.5 × 10^5^ cells per well in 3 mL DMEM F12 medium (Gibco, Thermo Fisher Scientific, Waltham, MA, USA, #31331-028) supplemented with 10% fetal calf serum (Eurobio, Ulis, France, #SVF001-01). Cells were treated with a range of concentrations of molecules and absolute ethanol was used as a negative control. MovS6 cells were then maintained at 37 °C in a humidified atmosphere containing 5% CO_2_. After 6 days of culture, the cells were washed with PBS 1X (Gibco, #CS1PBS501-01) and lysed with 1 mL of TL1 lysis buffer/well (0.5% sodium deoxycholate, 0.5% Triton-X100, 5 mM Tris-HCl pH 7.5). Cell lysates were collected and the protein concentration was quantified using a BCA Protein Assay kit (Thermo Fisher Scientific, Waltham, MA, USA, #23235) according to the supplier’s instructions.

### 3.6. Immunoblots

In order to determine whether the amount of PrP^Sc^ was modified by the treatment with the molecules, cell lysates (250 μg) were digested by 20 μg/mL of Proteinase K (PK, Thermo Fisher Scientific, #EO0491). PK digestion was stopped after 30 min by the addition of 4 mM of the anti-protease Pefabloc (Sigma Aldrich, #30827-99-7). PK-treated lysates were then centrifuged at 16,000× *g* for 30 min and protein pellets were solubilized in 15 μL of SB2X buffer (4% SDS, 33% glycerol, 0.1% bromophenol blue, 1 × Tris-glycine pH 8.3, 130 mM DTT). The amount of basal PrP^C^ was also quantified using 25 μg of untreated proteins in CB1X buffer (5 mM Tris-HCl pH 6.8, 10% glycerol, 2% SDS, 0.005% bromophenol blue, 1.25% β-mercapto-ethanol). PK-treated and untreated cell lysates were denatured for 10 min at 95 °C. Proteins were separated in NUPAGE 10% Bis-Tris gels (Thermo Fisher Scientific, #NW00100BOX). The migration was carried out for 1 h at 140 V in 1X MES migration buffer (Invitrogen, Thermo Fisher Scientific, Waltham, MA, USA, #NP0002-02). Proteins were then transferred to a 0.45 μm nitrocellulose membrane (GE Healthcare, Chicago, IL, USA, #10600002) for 1 h at 0.5 A in transfer buffer (25 mM Tris-HCl pH 8.3, 192 mM glycine, 20% ethanol, 0.1% SDS). Membranes were then incubated overnight at 4 °C with 1/40,000 Sha31 primary antibody (Bertin Pharma, Montigny le Bretonneux, France, #A03213) in TBS-T (10 mM Tris-HCl pH 7.5, 150 mM NaCl, 0.05% Tween 20). Membranes were washed 5 times with TBS-T, then incubated with 1/3000 HRP-conjugated anti-mouse antibodies (Abcam, Cambridge, UK, #ab6789) for 1 h at room temperature. Membranes corresponding to proteins not treated with PK were also incubated with 1/40,000 Sha31 primary antibody and then with 1/3000 anti-tubulin antibody (Abcam, #ab6161) as a loading control, followed by an incubation with 1/3000 HRP-conjugated anti-rat antibodies (Calbiochem, Merck, Kenilworth, NJ, USA, #401416). After 5 more washes with TBS-T, the immunoreactivity of PK-treated and PK-untreated proteins was revealed by chemiluminescence (SuperSignalTM West Pico Plus Chemiluminescent Substrate #34577, Thermo Fisher Scientific, Waltham, MA, USA), imaged via the Vilbert Lourmat imaging system and quantified using Image J software (1.53t version).

### 3.7. Cytoprotection Assay

To carry out the cytoprotection assay, 10,000 CHO-K1 cells [78] were plated in 96-well plates (Starlab, Orsay, France, #CC7682-7596) in Ham’s F-12 medium (Thermo Fisher Scientific, #10404972) supplemented with 10% fetal clone II serum (Hyclone, Thermo Fisher Scientific, Waltham, MA, USA, #SH3006603) at 37 °C with 5% CO_2_. After 24 h, the medium was replaced with a culture medium containing 0.45 µg/mL tunicamycin (Tm, Sigma Aldrich, Saint-Louis, MO, USA, #SML1287) and concentrations of molecules indicated on each figure or DMSO as a negative control. After 24 h of treatment, cell viability was measured by the quantification of the number of live cells using the WST-8 tetrazolium salt reduction into formazan (Abcam, Cambridge, UK, #ab228554). Then, 10 µL of WST-8 was added to each well and OD_450_ was measured after 4 h incubation at 37 °C with 5% CO_2_ using a Varioskan plate reader (Thermo Fisher Scientific, Waltham, MA, USA). Values are shown relative to the DMSO-treated cells, which is set at a value of 100% in each graph. Each treatment was performed in triplicate and each experiment was performed at least three times.

### 3.8. CHOP Expression Level

After 24 h treatment of CHO-K1 cells, luciferase activity was measured following the addition of 50 µL of Steady Glo lysis buffer (Promega, Madison, WI, USA, #E2510), using a Varioskan microplate reader (Thermo Fisher Scientific, Waltham, MA, USA).

### 3.9. Statistics and Data Analysis

Data analysis and statistics were performed with GraphPad Prism (GraphPad 8 Software, Dotmatics, Boston, MA, USA). The statistical significance of differences between experimental groups was calculated by one-way analysis of variance test followed by Dunnett’s post hoc test.

## 4. Conclusions

Here, we confirmed the anti-prion activity of purealidin Q (**1**), aplysamine-2 (**2**), and aplysamine-1 (**5**) against [*PSI*+] and [URE3] yeast prions and showed that these three bromotyrosine derivatives are potent anti-PrP^Sc^ compounds. We also described the capacity of aplyzanzine C (**7**), psammaplysene D (**11**), and anomoian F (**14**) to inhibit the propagation of [*PSI*+] and [URE3] yeast prions as well as the PrP^Sc^ mammalian prion. Finally, as endoplasmic reticulum (ER) stress due to misfolded protein aggregation is a hallmark of prion diseases [84,85], we evaluated the capacity of those bromotyrosine derivatives able to reduce PrP^Sc^ propagation to protect cells from ER stress. We showed that purealidin Q (**1**) and aplysamine-2 (**2**) had cytoprotective capacities on ER-stressed cells. Altogether, these data indicate that several metabolites from marine Verongiida sponges are potent anti-prion and ER-stress modulators, highlighting the importance of marine sponges as precious sources of bioactive molecules. The molecules identified here could also be beneficial for a broader range of diseases, as protein aggregation and ER stress are also characteristics of protein-misfolding diseases such as Parkinson’s and Alzheimer’s diseases.

## Figures and Tables

**Table 1:** Score of tested samples relative to their activities against [*PSI+*] and [URE3] yeast prions. Extracts or compounds were considered positive when scored 2 or 3.

Score	[*PSI+*] ^1^	[URE3] ^1^
0	−	nt
1	+ or +/−	−
2	+ or +/−	+/−
3	+ or +/−	+

^1^ +: active compound; +/−: weak activity; −: no activity; nt: not tested.

**Table 2:** Description of screened sponge extracts displaying anti-prion activity.

Family	Genus	Species	ID Sample	Location
*Aplysinellidae*	*Suberea*	*ianthelliformis*	P102-MNH2	PYF ^1^
*Aplysinellidae*	*Suberea*	*laboutei*	P562-WLF04	WLF ^2^
*Pseudoceratinidae*	*Pseudoceratina*	sp. (2081) ^3^	P281-TRAR04	PYF

^1^ French Polynesia; ^2^ Wallis & Futuna; ^3^ Queensland Museum’s operational taxonomic unit.

## Data Availability

All data generated are included in this article and its Supplementary Information. Additional data are available from the corresponding authors upon request.

## Supplementary Materials

The following supporting information can be downloaded at: https://www.mdpi.com/article/10.3390/md22100456/s1: Table S1. Activities of organic extracts of marine organisms tested in primary screening against [*PSI*+] and [URE3] yeast prions using 2 µL of each extract solubilized in DMSO at 10 mg/mL; Figure S1. Anti-prion activity of psammaplysene A; Figure S2. The toxic effect of purealidin Q (**1**), aplysamine-2 (**2**), aplysamine-1 (**5**), aplyzanzine C (**7**), psammaplysene D (**11**), and anomoian F (**14**) on CHO-K1 cells; Table S2. Comparison of the spectroscopic data of the compound **1** with the data from the literature concerning purealidin Q; Figure S3. Formula for compound **1** corresponding to purealidin Q; Figure S4. HR ESI MS spectrum in ACN compound **1**; Figure S5. NMR ^1^H in CD_3_OD of compound **1**; Figure S6. NMR COSY ^1^H-^1^H in CD_3_OD of compound **1**; Figure S7. NMR JMOD ^13^C in CD_3_OD of compound **1**; Figure S8. NMR HMQC ^1^H-^13^C in CD_3_OD of compound **1**; Figure S9. NMR HMBC ^1^H-^13^C in CD_3_OD of compound **1**; Table S3. Comparison of the spectroscopic data of compound **2** with data from the literature concerning aplysamine-2; Figure S10. Formula for compound **2** corresponding to aplysamine-2; Figure S11. HR ESI MS spectrum in ACN compound **2**; Figure S12. NMR ^1^H in CD_3_OD of compound **2**; Figure S13. NMR COSY ^1^H-^1^H in CD_3_OD of compound **2**; Figure S14. NMR JMOD ^13^C in CD_3_OD of compound **2**; Figure S15. NMR HMQC ^1^H-^13^C in CD_3_OD of compound **2**; Figure S16. NMR HMBC ^1^H-^13^C in CD_3_OD of compound **2**; Table S4. Comparison of spectroscopic data on compound 3 with data from the literature concerning pseudoceratinine A; Figure S17. Formula for compound **3** corresponding to pseudoceratinine A; Figure S18. HR ESI MS spectrum of compound **3**; Figure S19. NMR ^1^H in CD_3_OD of compound **3**; Figure S20. NMR COSY ^1^H-^1^H in CD_3_OD of compound **3**; Figure S21. NMR JMOD ^13^C in CD_3_OD of compound **3**; Figure S22. NMR HMQC ^1^H-^13^C in CD_3_OD of compound **3**; Figure S23. NMR HMBC ^1^H-^13^C in CD_3_OD of compound **3**; Table S5. Comparison of the spectroscopic data of compound **4** with data from the literature concerning aero-phobin-2; Figure S24. Formula for compound **4** corresponding to aerophobin-2; Figure S25. HR ESI MS spectrum of compound **4**; Figure S26. NMR ^1^H in CD_3_OD of compound **4**; Figure S27. NMR COSY ^1^H-^1^H in CD_3_OD of compound **4**; Figure S28. NMR JMOD ^13^C in CD_3_OD of compound **4**; Figure S29. NMR HMQC ^1^H-^13^C in CD_3_OD of compound **4**; Figure S30. NMR HMBC ^1^H-^13^C in CD_3_OD of compound **4**; Table S6. Comparison of the spectroscopic data of compound **5** with data from the literature concerning aplysamine-1; Figure S31. Formula for compound **5** corresponding to aplysamine-1; Figure S32. HRESIMS of compound **5**; Figure S33. NMR ^1^H in CD_3_OD of compound **5**; Figure S34. NMR COSY ^1^H-^1^H in CD_3_OD of compound **5**; Figure S35. NMR JMOD ^13^C in CD_3_OD of compound **5**; Figure S36. NMR HSQC ^1^H-^13^C in CD_3_OD of compound **5**; Figure S37. NMR HMBC ^1^H-^13^C in CD_3_OD of compound **5**; Table S7. Comparison of the spectroscopic data of compound 6 with data from the literature concerning pseudoceratinine B; Figure S38. Formula for compound 6 corresponding to pseudoceratinine B; Figure S39. HRESIMS of compound **6**; Figure S40. NMR ^1^H in CD_3_OD of compound **6**; Figure S41. NMR COSY ^1^H-^1^H in CD_3_OD of compound **6**; Figure S42. NMR HSQC ^1^H-^13^C in CD_3_OD of compound **6**; Figure S43. NMR HMBC ^1^H-^13^C in CD_3_OD of compound **6**. Figure S44. Original blots related to Figure S1; Figure S45. Original blots related to Figure 4—part 1/2; Figure S46. Original blots related to Figure 4—part 2/2; Figure S47. Original blots related to Figure 7—part 1/2; Figure S48. Original blots related to Figure 7—part 2/2.
