# Supplementary material for: Potential of Marine Sponge Metabolites against Prions: Bromotyrosine Derivatives, a Family of Interest
Source: Mar Drugs. 2024 Oct 4;22(10):456. doi: 10.3390/md22100456 (PMC11509309; doi:10.3390/md22100456)
Supplement: Supplementary file 1 [file marinedrugs-22-00456-s001.zip › marinedrugs-3183438-supplementary.pdf]

Supporting information

Potential of marine sponge metabolites against prions: Bromotyrosine derivatives, a family of interest.

Maha Sinane [1,†], Colin Grunberger [2,†], Lucile Gentile [1,†], Céline Moriou [3,†], Victorien Chaker [1], Pierre Coutrot [1], Alain Guenneguez [2], Marie-Aude Poullaouec [2], Solène Connan [2], Valérie Stiger-Pouvreau [2], Mayalen Zubia [4], Yannick Fleury [5], Stéphane Cérantola [6], Nelly Kervarec [7], Ali Al-Mourabit [3,‡], Sylvain Petek [2,‡,*] and Cécile Voisset [1,8,‡,*]

1 Univ Brest, Inserm, EFS, UMR 1078, GGB, School of Medicine, F-29200 Brest, France; maha.sinane@univ-brest.fr (M.S.); lucile.gentile@univ-brest.fr (L.G.); vico.chaker@laposte.net (V.C.); pcoutrot@outlook.fr (P.C.)

2 IRD, CNRS, Ifremer, Univ Brest, LEMAR, IUEM, F-29280 Plouzane, France; colin.grunberger@ird.fr (C.G.); alain.guenneguez@ird.fr (A.G.); marie-aude.poullaouec@univ-brest.fr (M.-A.P.); solene.connan@univ-brest.fr (S.C.); valerie.stiger@univ-brest.fr (V.S.-P.)

3 CNRS, Institut de Chimie des Substances Naturelles, Université Paris-Saclay, F-91190 Gif-sur-Yvette, France; celine.moriou@cnrs.fr (C.M.); ali.almourabit@cnrs.fr (A.A.-M.)

4 UPF, Ifremer, ILM, IRD, UMR 241 SECOPOL, BP6570, 98702 Faa'a, Tahiti, French Polynesia; mayalen.zubia@upf.pf (M.Z.)

5 Univ Brest, Univ Bretagne Sud, CNRS, LBCM, EMR 6076, F-29000 Quimper, France; yannick.fleury@univ-brest.fr (Y.F.)

6 Univ Brest, Plateforme RMN-RPE, F-29238 Brest, France; stephane.cerantola@univ-brest.fr (S.C.)

7 Univ Brest, Plateforme Spectrométrie de masse, F-29238 Brest, France; nelly.kervarec@univ-brest.fr (N.K.)

8 Univ Brest, Inserm, UMR 1101, LaTIM, School of Medicine, F-29200 Brest, France

† These authors contributed equally to this work.

‡ These authors contributed equally to this work.

* Correspondence: cecile.voisset@inserm.fr (C.V.); sylvain.petek@ird.fr (S.P.)

Table of Contents

Table S1. Activities of organic extracts of marine organisms tested in primary screening against [*PSI*+] and [URE3] yeast prions using 2 μL of each extract solubilized in DMSO at 10 mg/mL.
Figure S1. Anti-prion activity of psammaplysene A.
Figure S2. The toxic effect of purealidine Q (**1**), aplysamine-2 (**2**), aplysamine-1 (**5**), aplyzanzine C (**7**), psammaplysene D (**11**) and anomoian F (**14**) on CHO-K1 cells.
Table S2. Comparison of the spectroscopic data of the compound **1** with the data from the literature concerning purealidin Q
Figure S3. Formula for compound **1** corresponding to purealidin Q
Figure S4. HR ESI MS spectrum in ACN compound **1**
Figure S5. NMR ^{1}H in CD_3OD of compound **1**
Figure S6. NMR COSY ^{1}H-^{1}H in CD_3OD of compound **1**
Figure S7. NMR JMOD ^{13}C in CD_3OD of compound **1**
Figure S8. NMR HSQC ^{1}H-^{13}C in CD_3OD of compound **1**
Figure S9. NMR HMBC ^{1}H-^{13}C in CD_3OD of compound **1**
Table S3. Comparison of the spectroscopic data of compound **2** with data from the literature concerning aplysamine-2
Figure S10. Formula for compound **2** corresponding to aplysamine-2
Figure S11. HR ESI MS spectrum in ACN compound **2**
Figure S12. NMR ^{1}H in CD_3OD of compound **2**
Figure S13. NMR COSY ^{1}H-^{1}H in CD_3OD of compound **2**
Figure S14. NMR JMOD ^{13}C in CD_3OD of compound **2**
Figure S15. NMR HSQC ^{1}H-^{13}C in CD_3OD of compound **2**
Figure S16. NMR HMBC ^{1}H-^{13}C in CD_3OD of compound **2**
Table S4. Comparison of the spectroscopic data of compound 3 with data from the literature concerning pseudoceratinine A
Figure S17. Formula for compound **3** corresponding to pseudoceratinine A
Figure S18. HR ESI MS spectrum of compound **3**
Figure S19. NMR ^{1}H in CD_3OD of compound **3**
Figure S20. NMR COSY ^{1}H-^{1}H in CD_3OD of compound **3**
Figure S21. NMR JMOD ^{13}C in CD_3OD of compound **3**
Figure S22. NMR HSQC ^{1}H-^{13}C in CD_3OD of compound **3**
Figure S23. NMR HMBC ^{1}H-^{13}C in CD_3OD of compound **3**
Table S5. Comparison of the spectroscopic data of compound **4** with data from the literature concerning aerophobin-2
Figure S24. Formula for compound **4** corresponding to aerophobin-2
Figure S25. HR ESI MS spectrum of compound **4**
Figure S26. NMR ^{1}H in CD_3OD of compound **4**
Figure S27. NMR COSY ^{1}H-^{1}H in CD_3OD of compound **4**
Figure S28. NMR JMOD ^{13}C in CD_3OD of compound **4**
Figure S29. NMR HSQC ^{1}H-^{13}C in CD_3OD of compound **4**
Figure S30. NMR HMBC ^{1}H-^{13}C in CD_3OD of compound **4**
Table S6. Comparison of the spectroscopic data of compound **5** with data from the literature concerning aplysamine-1
Figure S31. Formula for compound **5** corresponding to aplysamine-1
Figure S32. HRESIMS of compound **5**
Figure S33. NMR ^{1}H in CD_3OD of compound **5**
Figure S34. NMR COSY ^{1}H-^{1}H in CD_3OD of compound **5**
Figure S35. NMR JMOD ^{13}C in CD_3OD of compound **5**
Figure S36. NMR HSQC ^{1}H-^{13}C in CD_3OD of compound **5**
Figure S37. NMR HMBC ^{1}H-^{13}C in CD_3OD of compound **5**
Table S7. Comparison of the spectroscopic data of compound **6** with data from the literature concerning pseudoceratinine B
Figure S38. Formula for compound **6** corresponding to pseudoceratinine B
Figure S39. HRESIMS of compound **6**
Figure S40. NMR ^{1}H in CD_3OD of compound **6**
Figure S41. NMR COSY ^{1}H-^{1}H in CD_3OD of compound **6**
Figure S42. NMR HSQC ^{1}H-^{13}C in CD_3OD of compound **6**
Figure S43. NMR HMBC 1H-13C in CD3OD of compound 6
Figure S44. Original blots related to Figure S1
Figure S45. Original blots related to Figure 4 – part 1/2
Figure S46. Original blots related to Figure 4 – part 2/2
Figure S47. Original blots related to Figure 7 – part 1/2
Figure S48. Original blots related to Figure 7 – part 2/2

Table S1. Activities of organic extracts of marine organisms tested in primary screening against [*PSI*+] and [URE3] yeast prions using 2 μL of each extract solubilized in DMSO at 10 mg/mL. Screening scores were attributed according to Table 1.

Family	Genus	Species	Score	ID	Extr.	Count.	Location	
**Algae**								
Caulerpaceae	*Caulerpa*	*chemnitzia*	0	A055	FACN	PYF[1]	Tubuai	[56]
Caulerpaceae	*Caulerpa*	*sertularioides*	0	A058	FACN	PYF	Tubuai	[56]
Caulerpaceae	*Caulerpa*	*taxifolia*	0	A057	FACN	PYF	Tubuai	[56]
Corallinaceae	*Jania*	*acutiloba*	0	A036	FM, FD	PYF	Tahiti	[56]
Cystocloniaceae	*Hypnea*	sp.	0	A053	FM, FD	PYF	Moorea	[56]
Dasyaceae	*Dasya*	*iyengarii*	0	A042	FM, FD	PYF	Moorea	[56]
Dictyotaceae	*Dictyota*	*bartayresiana*	0	A003	FM, FD	PYF	Tahiti	[56]
Dictyotaceae	*Dictyota*	*hamifera*	0	A034	FM	PYF	Tahiti	[56]
Dictyotaceae	*Padina*	*boryana*	0	A006	FM, FD	PYF	Tahiti	[56]
Dictyotaceae	*Spatoglossum*	*asperum*	0	A038	FM, FD	PYF	Tahiti	[56]
Galaxauraceae	*Galaxaura*	*rugosa*	0	A001	FM, FD	PYF	Tahiti	[56]
Gelidiellaceae	*Gelidiella*	*acerosa*	0	A035	FM, FD	PYF	Tahiti	[56]
Halimedaceae	*Halimeda*	*macroloba*	0	A010	FM, FD	PYF	Tahiti	[56]
Halimedaceae	*Halimeda*	*minima*	0	A025	FM, FD	PYF	Tahiti	[56]
Halimedaceae	*Halimeda*	*opuntia*	0	A011	FM, FD	PYF	Tahiti	[56]
Rhodomelaceae	*Acanthophora*	*spicifera*	0	A023	FM, FD	PYF	Tahiti	[56]
Rhodomelaceae	*Amansia*	*rhodantha*	0	A005	FM, FD	PYF	Tahiti	[56]
Sargassaceae	*Sargassum*	*pacificum*	0	A004	FM, FD	PYF	Tahiti	[56]
Sargassaceae	*Turbinaria*	*ornata*	0	A007	FM, FD	PYF	Tahiti	[56]
Scytosiphonaceae	*Chnoospora*	*implexa*	0	A002	FM, FD	PYF	Tahiti	[56]
Scytosiphonaceae	*Chnoospora*	*minima*	0	A032	FM, FD	PYF	Tahiti	[56]
Scytosiphonaceae	*Colpomenia*	*sinuosa*	0	A029	FM	PYF	Tahiti	[56]
Scytosiphonaceae	*Hydroclathrus*	*clathratus*	0	A049	FM, FD	PYF	Moorea	[56]
Scytosiphonaceae	*Rosenvingea*	*intricata*	0	A050	FM	PYF	Moorea	[56]
Udoteaceae	*Chlorodesmis*	*fastigiata*	0	A037	FM, FD	PYF	Tahiti	[56]
**Sponges**								
Ancorinidae	*Jaspis*	sp. (4187)[2]	0	P600-WLF26	XDM	WLF[3]	Wallis	[55]
Ancorinidae	*Rhabdastrella*	sp. (4891)	0	P510-ST30	XDM	PYF	Tahiti	[53]
Aplysinellidae	*Aplysinella*	*rhax*	0	P164-MHO6	XDM	PYF	Hiva Oa	[51]
**Aplysinellidae**	***Suberea***	***ianthelliformis***	**3**	P102-MNH2	XDM	PYF	Nuku Hiva	[51]
**Aplysinellidae**	***Suberea***	***laboutei***	**3**	P562-WLF04	XDM	WLF	Wallis	[55]
Aplysinellidae	*Suberea*	sp. (2093)	0	P110-MNH4	XDM	PYF	Nuku Hiva	[51]
Aplysinellidae	*Suberea*	sp. (2121)	0	P76-STA1	XDM	PYF	Tahaa	[51]
Aplysinidae	*Aplysina*	sp. (2370)	0	P460-AT04	XDM	PYF	Tubuai	[54]
Astroscleridae	*Astrosclera*	*willeyana*	0	P616-WLF16	XDM	WLF	Wallis	[55]
Axinellidae	*Phycopsis*	sp. (1640)	0	P195-MFH1	XDM	PYF	Fatu Hiva	[51]
Chalinidae	*Chalinula*	sp. (4223)	0	P435-AM04	XDM	PYF	Marotiri	[54]
Chalinidae	*Haliclona*	sp. (4386)	0	P259-TFAK01	XDM	PYF	Fakarava	[52]
Chalinidae	*Haliclona*	sp. (4857)	0	P274-TRAR01	XDM	PYF	Rangiroa	[52]

Chalinidae	*Haliclona*	sp. (4858)	0	P276-TRAR02	XDM	PYF	Rangiroa	[52]
Chalinidae	*Haliclona*	sp. (1971)	0	P602-WLF26	XDM	WLF	Wallis	[55]
Chalinidae	*Haliclona*	sp. (4468)	0	P296-TFAK03	XDM	PYF	Fakarava	[52]
Chalinidae	*Haliclona*	sp. (4499)	0	P575-WLF04	XDM	WLF	Wallis	[55]
Chalinidae	*Haliclona*	sp. (4999)	0	P574-WLF11	XDM	WLF	Wallis	[55]
Chalinidae	*Haliclona*	sp. (854)	0	P73-STA1	XDM	PYF	Tahaa	[51]
Chalinidae	*Haliclona (reniera)*	sp. (2555)	0	P272-TRAR04	XDM	PYF	Rangiroa	[52]
Chondropsidae	*Chondropsis*	sp. (4218)	0	P285-TTAK01	XDM	PYF	Takaroa	[52]
Chondropsidae	*Phoriospongia*	sp. nov. (3715)	0	P171-MHO8	XDM	PYF	Hiva Oa	[51]
Chondropsidae	*Psammoclema*	sp. (4798)	0	P243-THA04	XDM	PYF	Hao	[52]
Chondropsidae	*Strongylacidon*	sp. (2535)	0	P273-TRAR01	XDM	PYF	Rangiroa	[52]
Chondrosiidae	*Chondrosia*	*corticata*	0	P123-MNH11	XDM	PYF	Nuku Hiva	[51]
Chondrosiidae	*Chondrosia*	sp. (2212)	0	P494-ST30	XDM	PYF	Tahiti	[53]
Clionaidae	*Spheciospongia*	*potamophera*	0	P270-TFAK06_(+05)	XDM	PYF	Fakarava	[52]
Clionaidae	*Spheciospongia*	sp. nov. (1142)	0	P103-MNH4	XDM	PYF	Nuku Hiva	[51]
Coelosphaeridae	*Coelosphaera*	sp. nov. (4683)	0	P106-MNH1	XDM	PYF	Nuku Hiva	[51]
Coelosphaeridae	*Lissodendoryx*	sp. (1281)	0	P622-WLF13	XDM	WLF	Wallis	[55]
Crambeidae	*Monanchora*	*unguiculata*	1	P176-MT2	XDM	PYF	Taruata	[51]
Darwinellidae	*Aplysilla*	sp. (2034)	0	P573-WLF02	XDM	WLF	Wallis	[55]
Darwinellidae	*Chelonaplysilla*	*delicata*	0	P658-WLF23	XDM	WLF	Wallis	[55]
Darwinellidae	*Darwinella*	sp. (4848)	0	P236-TA02	XDM	PYF	Anuanuraro	[52]
Darwinellidae	*Dendrilla*	sp. (2575)	0	P571-WLF02	XDM	WLF	Wallis	[55]
Darwinellidae	*Dendrilla*	sp. (4907)	0	P328-TRAN10	XDM	PYF	Rangiroa	[52]
Desmacellidae	*Neofibularia*	*hartmani*	0	P489-ST28	XDM	PYF	Tahiti	[53]
Dictyodendrillidae	*Acanthodendrilla*	cf. sp. (1948)	0	P586-WLF23	XDM	WLF	Wallis	[55]
Dictyonellidae	*Acanthella*	*pulcherrima*	0	P258-TFAK01	XDM	PYF	Fakarava	[52]
Dictyonellidae	*Rhaphoxya*	*pallida*	0	P597-WLF18	XDM	WLF	Wallis	[55]
Dictyonellidae	*Rhaphoxya*	sp. (4868)	0	P518-ST33	XDM	PYF	Tahiti	[53]
Dictyonellidae	*Stylissa*	*flabelliformis*	0	P4-ST49	XDM	PYF	Tahiti	[53]
Dysideidae	*Dysidea*	*cf. arenaria*	0	P339-TTIK04	XDM	PYF	Tikehau	[52]
Dysideidae	*Dysidea*	*cf. pallescens*	0	P633-WLF13	XDM	WLF	Wallis	[55]
Dysideidae	*Dysidea*	*lizardensis*	0	P572-WLF02	XDM	WLF	Wallis	[55]
Dysideidae	*Dysidea*	sp. (103)	0	P464-AT11	XDM	PYF	Tubuai	[54]
Dysideidae	*Dysidea*	sp. (2328)	0	P549-ST52	XDM	PYF	Tahiti	[53]
Dysideidae	*Euryspongia*	*delicatula*	0	P248-THA05	XDM	PYF	Hao	[52]
Dysideidae	*Euryspongia*	sp. (4409)	1	P52-SR11	XDM	PYF	Raiatea	[51]
Dysideidae	*Lamellodysidea*	*herbacea*	0	P57-SR4	XDM	PYF	Raiatea	[51]
Dysideidae	*Lamellodysidea*	sp. (4895)	0	P331-TRAN10	XDM	PYF	Rangiroa	[52]
Grantiidae	*Leucandra*	sp. (4854)	0	P455-ARAI10	XDM	PYF	Raivavae	[52]
Halichondriidae	*Amorphinopsis*	sp. (4757)	1	P96-SH1	XDM	PYF	Huahiné	[51]
Halichondriidae	*Axinyssa*	*aculeata*	0	P280-TRAR04	XDM	PYF	Rangiroa	[52]
Halichondriidae	*Axinyssa*	sp. (3251)	0	P588-WLF05	XDM	WLF	Wallis	[55]
Halichondriidae	*Axinyssa*	sp. (4849)	0	P249-THA06	XDM	PYF	Hao	[52]
Halichondriidae	*Epipolasis*	sp. (452)	0	P111-MNH4	XDM	PYF	Nuku Hiva	[51]

Halichondriidae	*Halichondria*	sp. (4804)	0	P548-ST49	XDM	PYF	Tahiti	[53]
Halichondriidae	*Halichondrida*	sp. (1429)	0	P607-WLF08	XDM	WLF	Wallis	[55]
Halichondriidae	*Halichondrida*	sp. (1984)	0	P634-WLF13	XDM	WLF	Wallis	[55]
Halichondriidae	*Hymeniacidon*	sp. (4898)	1	P334-TRAN10	XDM	PYF	Rangiroa	[52]
Halichondriidae	*Topsentia*	sp. nov. (4695)	0	P202-MFH2	XDM	PYF	Fatu Hiva	[51]
Halisarcidae	*Halisarca*	sp. (1231)	1	P242-THA04	XDM	PYF	Hao	[52]
Heteroxyidae	*Myrmekioderma*	sp. (4997)	0	P564-WLF01	XDM	WLF	Wallis	[55]
Hymedesmiidae	*Hooperia*	*anfractuosa*	1	P149-MUP8	XDM	PYF	Ua Pou	[51]
Hymerhabdiidae	*Hymerhabdia*	sp. nov. (4743)	0	P117-MNH5	XDM	PYF	Nuku Hiva	[51]
Ianthellidae	*Ianthella*	*reticulata*	0	P284-TTAK01	XDM	PYF	Takaroa	[52]
Irciniidae	*Ircinia*	sp. (1244)	0	P608-WLF08	XDM	WLF	Wallis	[55]
Irciniidae	*Ircinia*	sp. (2707)	0	P640-WLF17	XDM	WLF	Wallis	[55]
Irciniidae	*Psammocinia*	sp. (1944)	1	P596-WLF16	XDM	WLF	Wallis	[55]
Irciniidae	*Psammocinia*	sp. (4758)	0	P49-SR1	XDM	PYF	Raiatea	[51]
Irciniidae	*Psammocinia*	sp. (704)	0	P495-ST30	XDM	PYF	Tahiti	[53]
Leucaltidae	*Leucaltis*	sp.	0	P177-MT1	XDM	PYF	Taruata	[51]
Leucettidae	*Leucetta*	*chagosensis*	1	P266-TRAN01	XDM	PYF	Rangiroa	[52]
Leucettidae	*Leucetta*	*chagosensis*	0	P605-WLF08	XDM	WLF	Wallis	[55]
Leucettidae	*Leucetta*	*microraphis*	0	P82-STA6	XDM	PYF	Tahaa	[51]
Leucettidae	*Pericharax*	*heteroraphis*	0	P582-WLF07	XDM	WLF	Wallis	[55]
Leucettidae	*Pericharax*	sp. (2065)	0	P609-WLF26	XDM	WLF	Wallis	[55]
Leucettidae	*Pericharax*	sp. (2116)	0	P618-WLF20	XDM	WLF	Wallis	[55]
Microcionidae	*Antho (antho)*	sp. nov. (4699)	0	P179-MT1	XDM	PYF	Taruata	[51]
Niphatidae	*Amphimedon*	sp. (2641)	0	P247-THA05	XDM	PYF	Hao	[52]
Niphatidae	*Cribrochalina*	sp. (4867)	0	P514-ST30	XDM	PYF	Tahiti	[53]
Oscarellidae	*Oscarella*	sp.	0	P589-WLF17	XDM	WLF	Wallis	[55]
Petrosiidae	*Neopetrosia*	*exigua*	1	P197-MFH1	XDM	PYF	Fatu Hiva	[51]
Petrosiidae	*Petrosia*	sp. (1895)	0	P660-WLF26	XDM	WLF	Wallis	[55]
Petrosiidae	*Petrosia*	sp. (2035)	0	P621-WLF10	XDM	WLF	Wallis	[55]
Petrosiidae	*Petrosia*	sp. (2721)	0	P559-WLF01	XDM	WLF	Wallis	[55]
Petrosiidae	*Petrosia*	sp. (4179)	0	P620-WLF12	XDM	WLF	Wallis	[55]
Petrosiidae	*Petrosia*	sp. (4893)	0	P482-ST27	XDM	PYF	Tahiti	[53]
Petrosiidae	*Xestospongia*	sp. nov (4688)	1	P107-MNH3	XDM	PYF	Nuku Hiva	[51]
Phloeodictyidae	*Siphonodictyon*	sp. (331)	0	P143-MUP3	XDM	PYF	Ua Pou	[51]
Phloeodictyidae	*Siphonodictyon*	sp. nov (4700)	0	P299-TTET02	XDM	PYF	Tetiaroa	[52]
Pseudoceratinidae	*Pseudoceratina*	sp. (1947)	0	P603-WLF27	XDM	WLF	Wallis	[55]
**Pseudoceratinidae**	***Pseudoceratina***	**sp. (2081)**	**3**	P281-TRAR04	XDM	PYF	Rangiroa	[52]
Raspailiidae	*Echinodictyum*	*asperum*	0	P561-WLF07	XDM	WLF	Wallis	[55]
Raspailiidae	*Echinodictyum*	sp. (4862)	0	P444-ARAP16	XDM	PYF	Rapa	[54]
Scleritodermidae	*Microscleroderma*	sp.	0	P109-MNH3	XDM	PYF	Nuku Hiva	[51]
Scopalinidae	*Stylissa*	*cf. carteri*	0	P563-WLF01	XDM	WLF	Wallis	[55]
Scopalinidae	*Stylissa*	*cf. carteri*	1	P601-WLF13	XDM	WLF	Wallis	[55]
Scopalinidae	*Stylissa*	*cf. carteri*	1	P628-WLF13	XDM	WLF	Wallis	[55]
Scopalinidae	*Stylissa*	*massa*	1	P579-WLF02	XDM	WLF	Wallis	[55]

Siphonidiidae	*Gastrophanella*	sp. nov	0	P516-ST30	XDM	PYF	Tahiti	[53]
Spirastrellidae	*Spirastrella*	sp. (3137)	0	P261-TMAK02+03	XDM	PYF	Makemo	[52]
Spirastrellidae	*Spirastrella*	sp. nov. (4689)	0	P157-MHO1+2	XDM	PYF	Hiva Oa	[51]
Spongiidae	*Coscinoderma*	sp. (4176)	0	P72-SR11	XDM	PYF	Raiatea	[51]
Spongiidae	*Leiosella*	sp. (6001)	0	P593-WLF06	XDM	WLF	Wallis	[55]
Spongiidae	*Rhopaloeides*	*odorabile*	0	P445-AT04	XDM	PYF	Tubuai	[54]
Spongiidae	*Spongia*	sp. (1983)	1	P265-TMAK05	XDM	PYF	Makemo	[52]
Spongiidae	*Spongia (heterofibria)*	sp. (4887)	0	P325-TRAN08	XDM	PYF	Rangiroa	[52]
Tetillidae	*Cinachyrella*	sp. nov. (4680)	1	P101-MNH1	XDM	PYF	Nuku Hiva	[51]
Tetillidae	*Cinachyrella*	sp. nov. (4680)	0	P560-WLF01	XDM	WLF	Wallis	[55]
Tetillidae	*Craniella*	*abracadabra*	0	P191-MT4	XDM	PYF	Taruata	[51]
Thorectidae	*Cacospongia*	sp. (2110)	0	P327-TRAN07	XDM	PYF	Rangiroa	[52]
Thorectidae	*Cacospongia*	sp. (2334)	0	P451-ARAI04	XDM	PYF	Raivavae	[54]
Thorectidae	*Cacospongia*	sp. (6009)	0	P642-WLF27	XDM	WLF	Wallis	[55]
Thorectidae	*Dactylospongia*	*elegans*	0	P120-MNH6	XDM	PYF	Nuku Hiva	[51]
Thorectidae	*Dactylospongia*	*metachromia*	0	P232-TFAK01	XDM	PYF	Fakarava	[52]
Thorectidae	*Dactylospongia*	sp. (4856)	0	P257-TFAK01	XDM	PYF	Fakarava	[52]
Thorectidae	*Dactylospongia*	sp. (4908)	0	P332-TRAN10	XDM	PYF	Rangiroa	[52]
Thorectidae	*Fascaplysinopsis*	*cf reticulata*	0	P319-TRAN06	XDM	PYF	Rangiroa	[52]
Thorectidae	*Fascaplysinopsis*	sp.	0	P646-WLF24	XDM	WLF	Wallis	[55]
Thorectidae	*Fascaplysinopsis*	sp. (1549)	2	P590-WLF06	XDM	WLF	Wallis	[55]
Thorectidae	*Fascaplysinopsis*	sp. (2071)	0	P287-TTAK03	XDM	PYF	Takaroa	[52]
Thorectidae	*Fascaplysinopsis*	sp. (4906)	0	P316-TRAN01	XDM	PYF	Rangiroa	[52]
Thorectidae	*Fascaplysinopsis*	sp. (6004)	0	P625-WLF19	XDM	WLF	Wallis	[55]
Thorectidae	*Hyrtios*	*cf. erectus*	0	P581-WLF05	XDM	WLF	Wallis	[55]
Thorectidae	*Hyrtios*	*erectus*	0	P587-WLF05	XDM	WLF	Wallis	[55]
Thorectidae	*Hyrtios*	sp. (3466)	0	P544-ST41	XDM	PYF	Tahiti	[53]
Thorectidae	*Luffariella*	sp. (1227)	0	P304-TTIK03	XDM	PYF	Tikehau	[52]
Thorectidae	*Smenospongia*	sp. (4760)	0	P62B-SR10	XDM	PYF	Raiatea	[51]
**Tunicates**								
Didemnidae	*Didemnum*	*granulatum*	0	T51-ST42	XDM	PYF	Tahiti	[53]
Didemnidae	*Didemnum*	*perlucidum*	0	T7-MNH13	XDM	PYF	Nuku Hiva	[51]
Didemnidae	*Didemnum*	sp.	0	T6-MNH11	XDM	PYF	Nuku Hiva	[51]
Didemnidae	*Lissoclinum*	*fragile*	0	T19-THA04	XDM	PYF	Hao	[52]
Polycitoridae	*Cystodytes*	*multipapillatus*	0	T31-TRAN05	XDM	PYF	Rangiroa	[52]
Pyuridae	*Herdmania*	*pallida*	0	T50-ST42	XDM	PYF	Tahiti	[53]
Pyuridae	*Herdmania*	*subpallida*	0	T41-ARAI11	XDM	PYF	Raivavae	[54]
Styelidae	*Polycarpa*	*pigmentata*	0	T2-MNH7	XDM	PYF	Nuku Hiva	[51]
**Microorganisms**				Pure compound			strain	
Pseudoalteromonadaceae	*Pseudoaltromonas*	*rhizosphaerae*	0	alt-954		FRA[4]	h*Cg*-6	[58]
Pseudoalteromonadaceae	*Pseudoaltromonas*	*rhizosphaerae*	0	alt-970		FRA	h*Cg*-6	[58]
Pseudoalteromonadaceae	*Pseudoaltromonas*	*rhizosphaerae*	0	alt-980		FRA	h*Cg*-6	[58]

[1] French Polynesia; [2] Queensland Museum's operational taxonomic unit; [3] Wallis & Futuna; [4] France

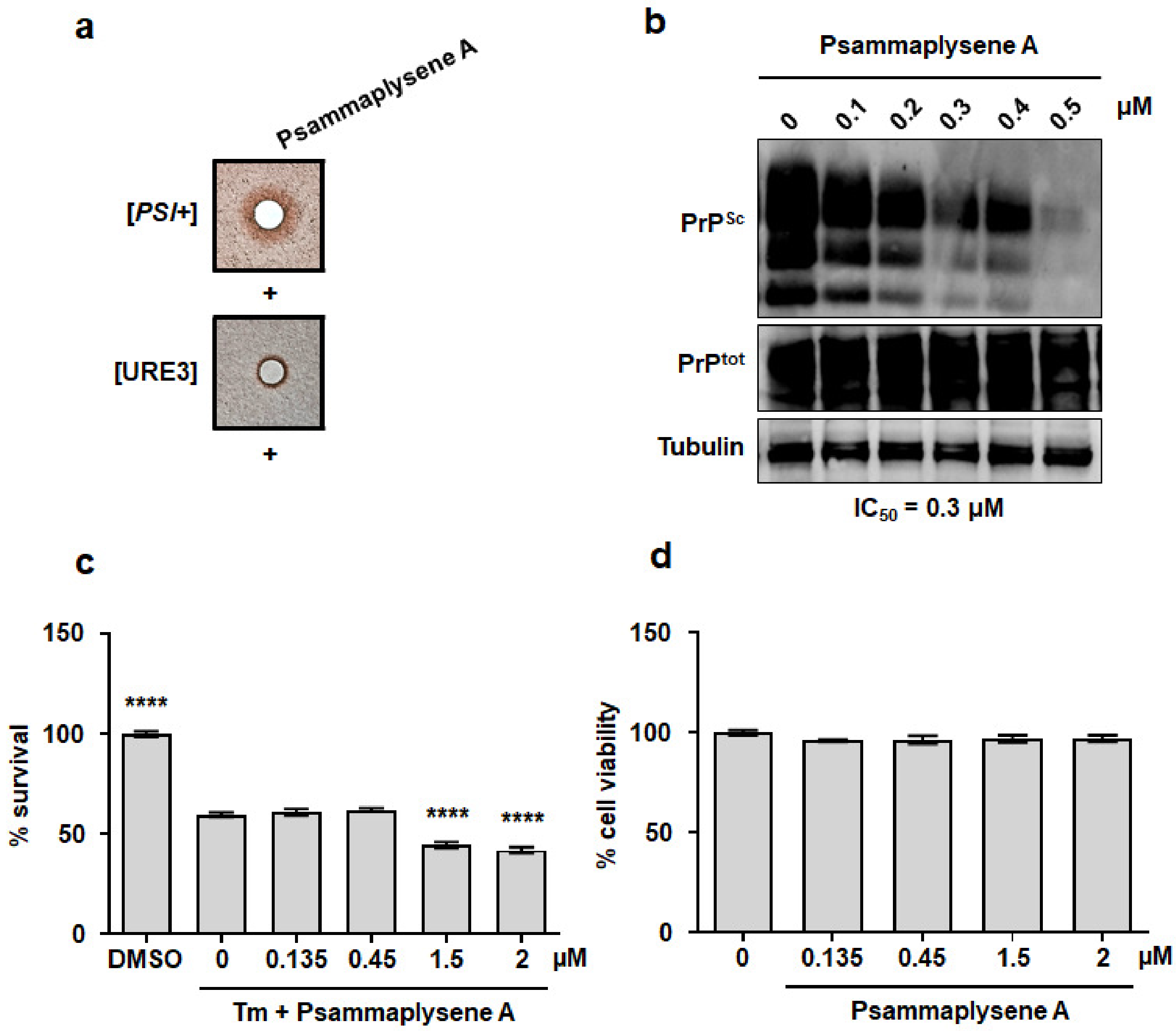

Figure S1. Anti-prion activity of psammaplysene A. **(a)** Psammaplysene A is active against [*PSI*+] and [URE3] yeast prions (load of 5 µL of 10 mM molecules, as described in Figure 1). **(b)** Psammaplysene A is also active against PrP^{Sc} prion as described in [35], with a toxicity from 1.8 µM on MovS6 cells. The blot shown is representative of three independent experiments which all produced similar results. **(c)** Psammaplysene A had no cytoprotective effect on ER-stressed CHO-K1 cells. A representative assay including three technical repeats is shown with SD error bars and each experiment was performed at least three times with similar results. **(d)** Psammaplysene A showed no toxicity at tested concentration but was toxic above 2 µM for CHO-K1 cells. Bar height represents the mean relative to Tm-treated cells. The values are shown as relative to the DMSO-treated cells, which is set to a value of 100%. A representative assay including three technical repeats is shown with SD error bars, and each experiment was performed at least three times with similar results. **** $p < 0.0001$ one-way ANOVA analysis followed by Dunnett's test.

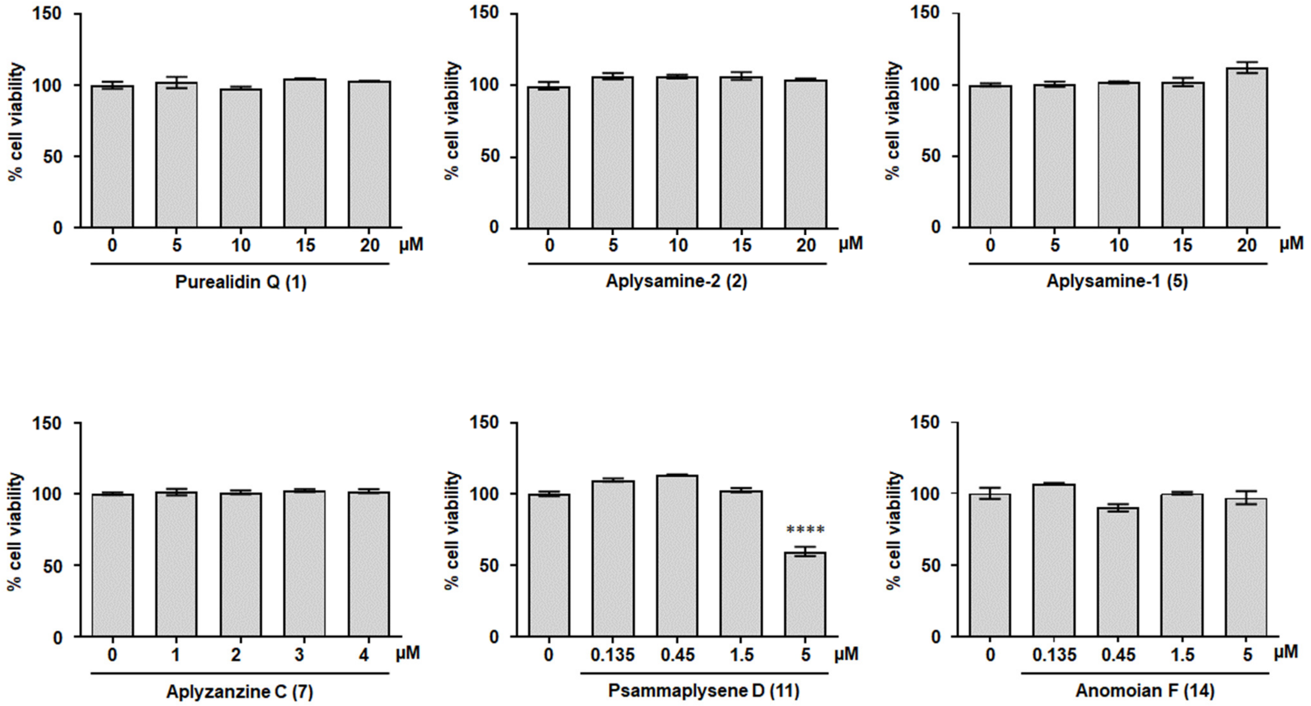

Figure S2. Toxic effect of purealidin Q (1), aplysamine-2 (2), aplysamine-1 (5), aplyzanzine C (7), psammaplysene D (11) and anomoian F (14) on CHO-K1 cells. CHO-K1 cells were treated for 24 h with increasing amounts of bromotyrosine derivatives. Cell survival was measured using the WST-8 tetrazolium salt. Purealidin Q (1), aplysamine-2 (2) and aplysamine-1 (5) were not toxic in the 5-20 µM range of concentrations used to monitor their cytoprotective effect (Figure 8). Aplyzanzine C (7) and psammaplysene D (11) were toxic from 5 µM and anomoian F (14) was toxic above 5 µM. The values are shown as relative to the DMSO-treated cells, which is set to a value of 100%. A representative assay including three technical repeats is shown with SD error bars and each experiment was performed at least three times with similar results. Bar height represents the mean relative to Tm-treated cells. **** $p<0.0001$ one-way ANOVA followed by Dunnett's test.

Table S2. Comparison of the spectroscopic data of compound **1** with data from the literature concerning purealidin Q.

	Compound **1**		Purealidin Q [46]	
		MS data		
	HRESIMS data showed the $[M+H]^+$ ion as a cluster at *m/z* 742, 744, 746, 748, 750, in a 1:4:6:4:1 ratio, indicating the presence of four bromine atoms in the molecule. Analysis of the pic $[M+H]^+$ at *m/z* 745,8722 (calc. for $C_{23}H_{28}N_3O_5{}^{79}Br_2{}^{81}Br_2$, 745.8729) allowed us to propose the molecular formula of **1** as $C_{23}H_{27}N_3O_5Br_4$.		FABMS $[M+H]^+$ *m/z* 742, 744, 746, 748, 750, in a 1:4:6:4:1 ratio. HR-FABMS $[M+H]^+$ calc. for $C_{23}H_{28}N_3O_5{}^{79}Br_2{}^{81}Br_2$, 745.8721, found 745.8729.	
		1D NMR data		
Position	in CD_3OD		^{13}C in CD_3OD	^1H in DMSO-*d6*
No	δ_C, type	δ_H mult, (*J* in Hz)	δ_C, type	δ_H mult, (*J* in Hz)
1	75.5, CH	4.08, s	75.5	3.92, d*, (8.2)
2	114.2, C		114.2	
3	149.3, C		149.3	
4	122.8, C		122.8	
5	132.2, CH	6.4, s	132.2	6.57, s
6	92.4, C		92.4	
7	40.1, CH_2	3.05, d (18.3)	41.4	3.18, d, (18.2)
7'	40.1, CH_2	3.78, d (18.3)	41.4	3.60, d, (18.2)
8	155.2, C		155.2	
9	161.6, C		161.6	
10	41.4, CH_2	3.48, td (7.2; 2.3)	40.1	3.36, m
11	35.1, CH_2	2.8, t (7.1)	35.1	2.76, t, (6.9)
12	140.0, C		140.0	
13	134.5, CH	7.49, s	134.5	7.54, s
14	118.8, C		118.8	
15	152.5, C		152.2	
16	71.8, CH_2	4.08, t (5.8)	71.2	3.99, t, (6.3)
17	27.4, CH_2	2.18, tt (8; 5.9)	26.4	2.15, m
18	57.3, CH_2	3.14, t (7.9)	57.2	3.4, m
O*Me*	60.4, CH_3	3.73, s	60.4	3.64, s
N*Me*2	44.4, CH_3	2.68, s	43.7	2.83, s
N*H*CO				8.58, t, (5.7)
O*H*				6.34, d*, (8.2)

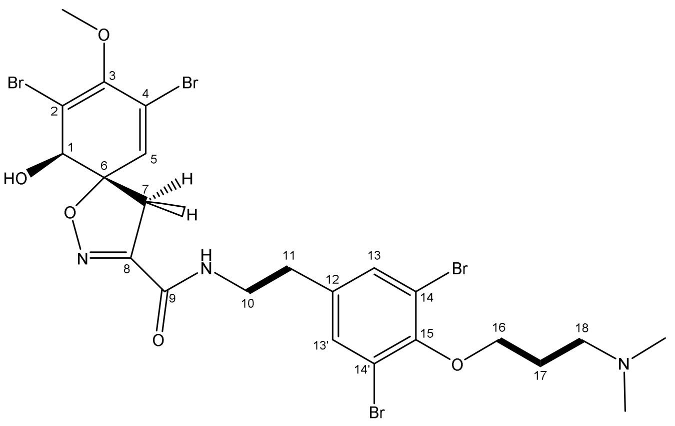

Figure S3. Formula for compound **1** corresponding to purealidin Q

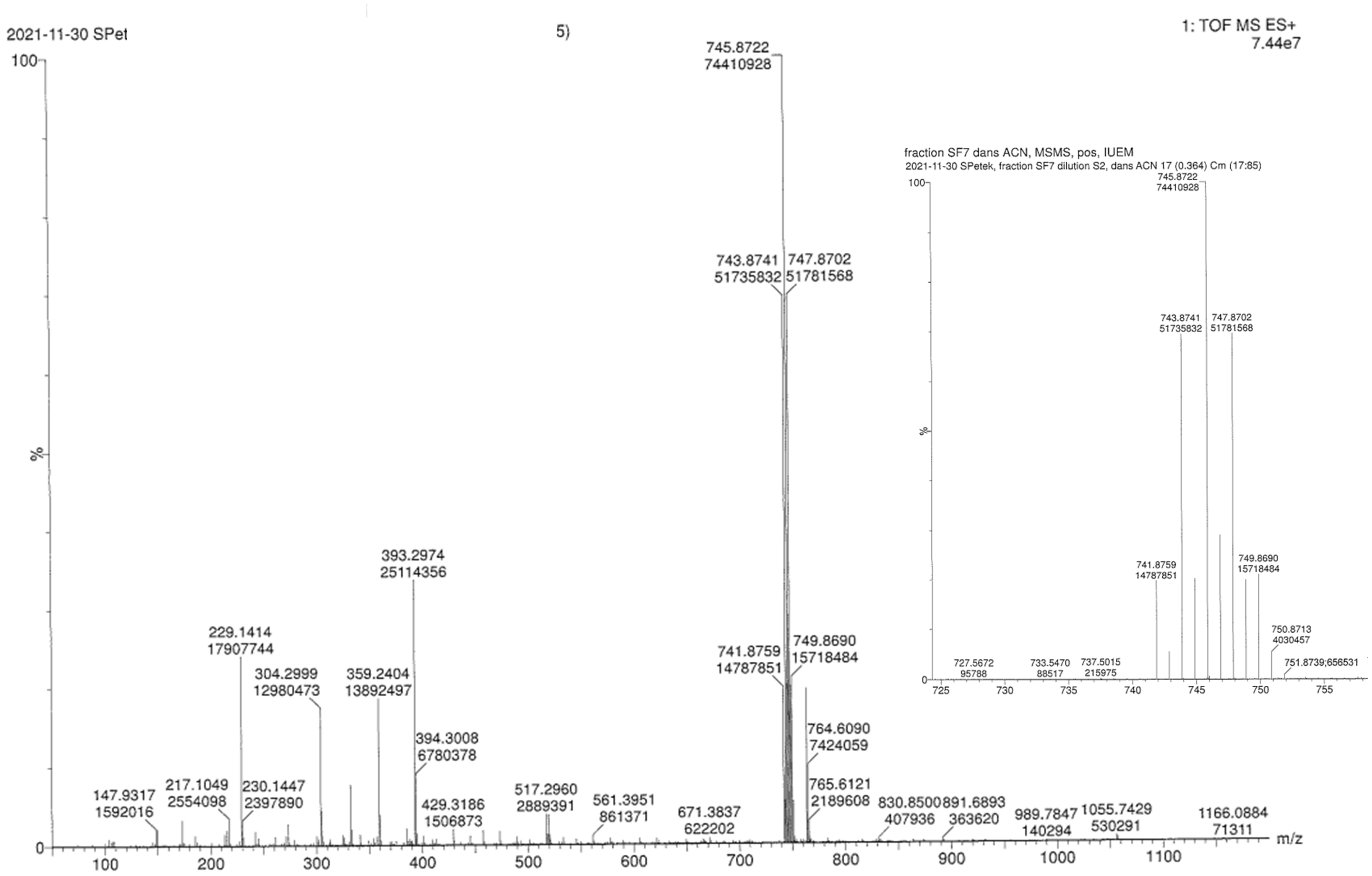

Figure S4. HR ESI spectrum in AcN compound **1**

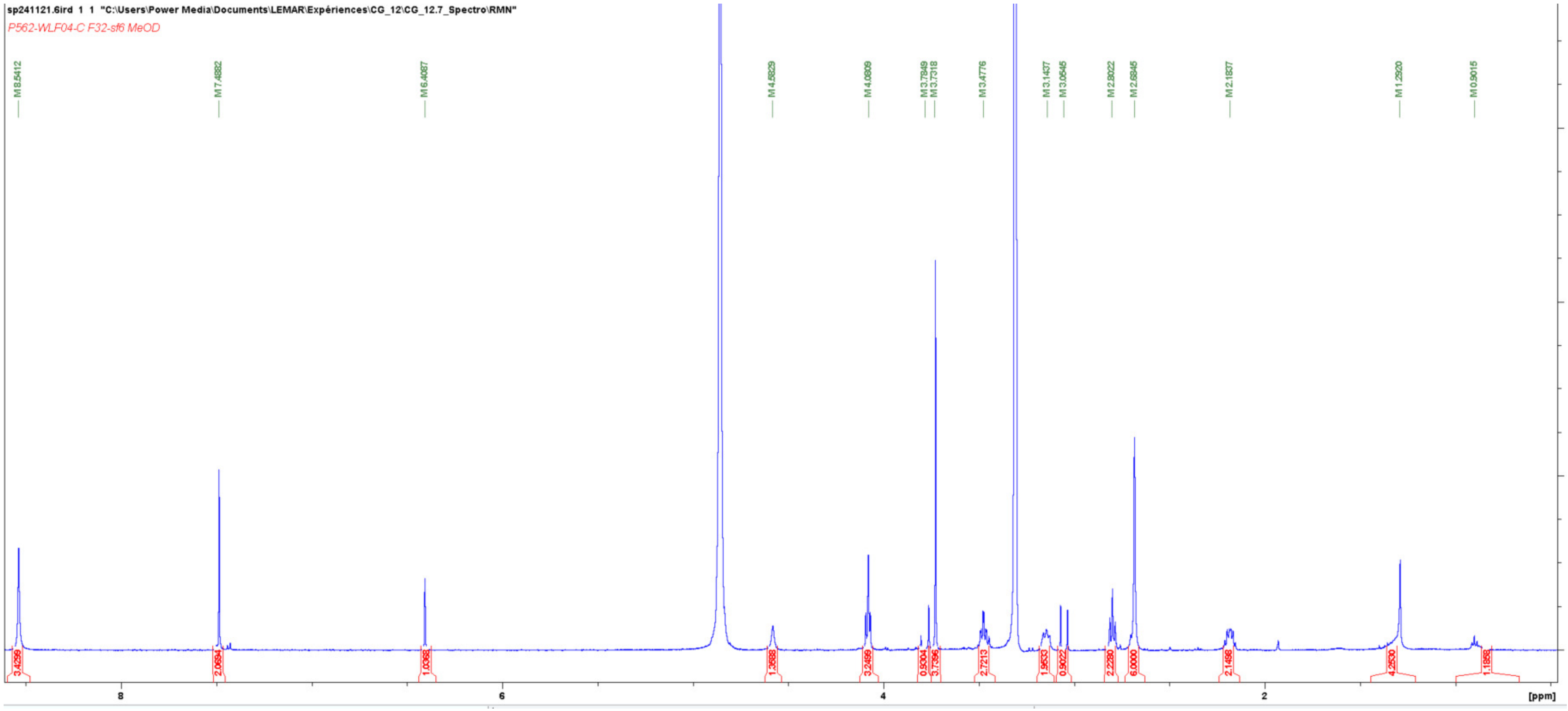

Figure S5. NMR ^{1}H in CD_3OD of compound **1**

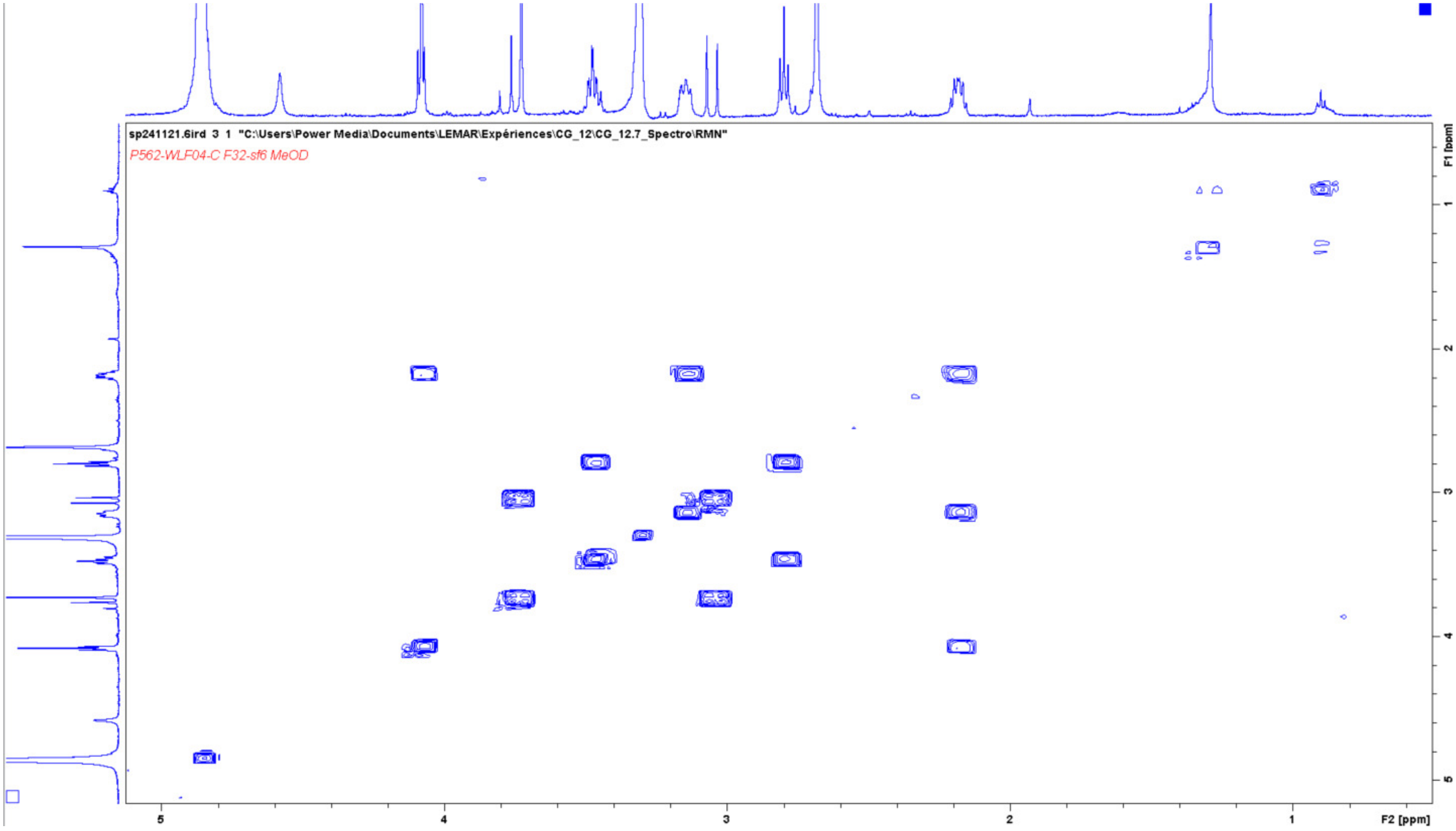

Figure S6. NMR COSY ^{1}H-^{1}H in CD_3OD of compound **1**

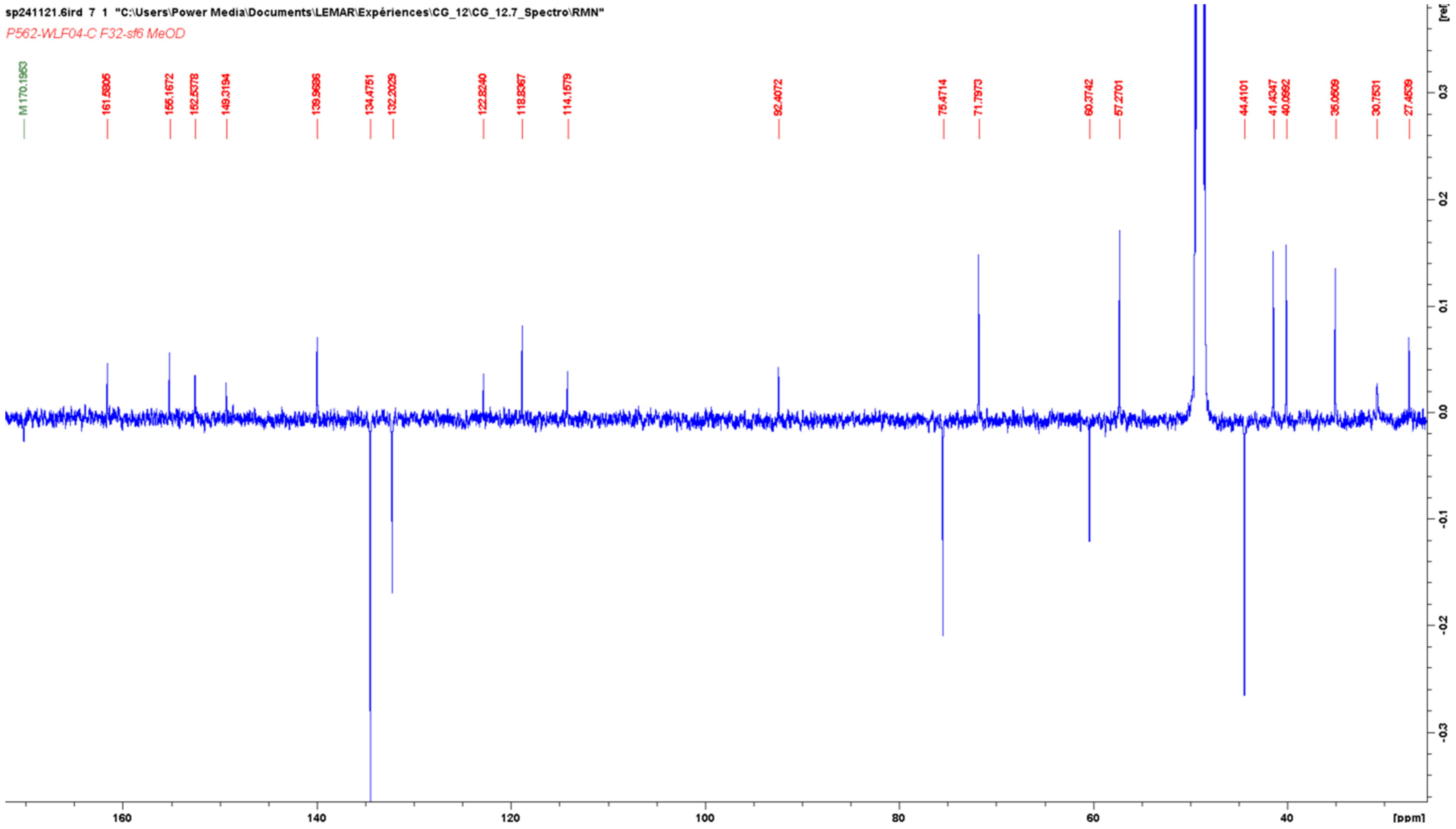

Figure S7. NMR JMOD ^{13}C in CD_3OD of compound **1**

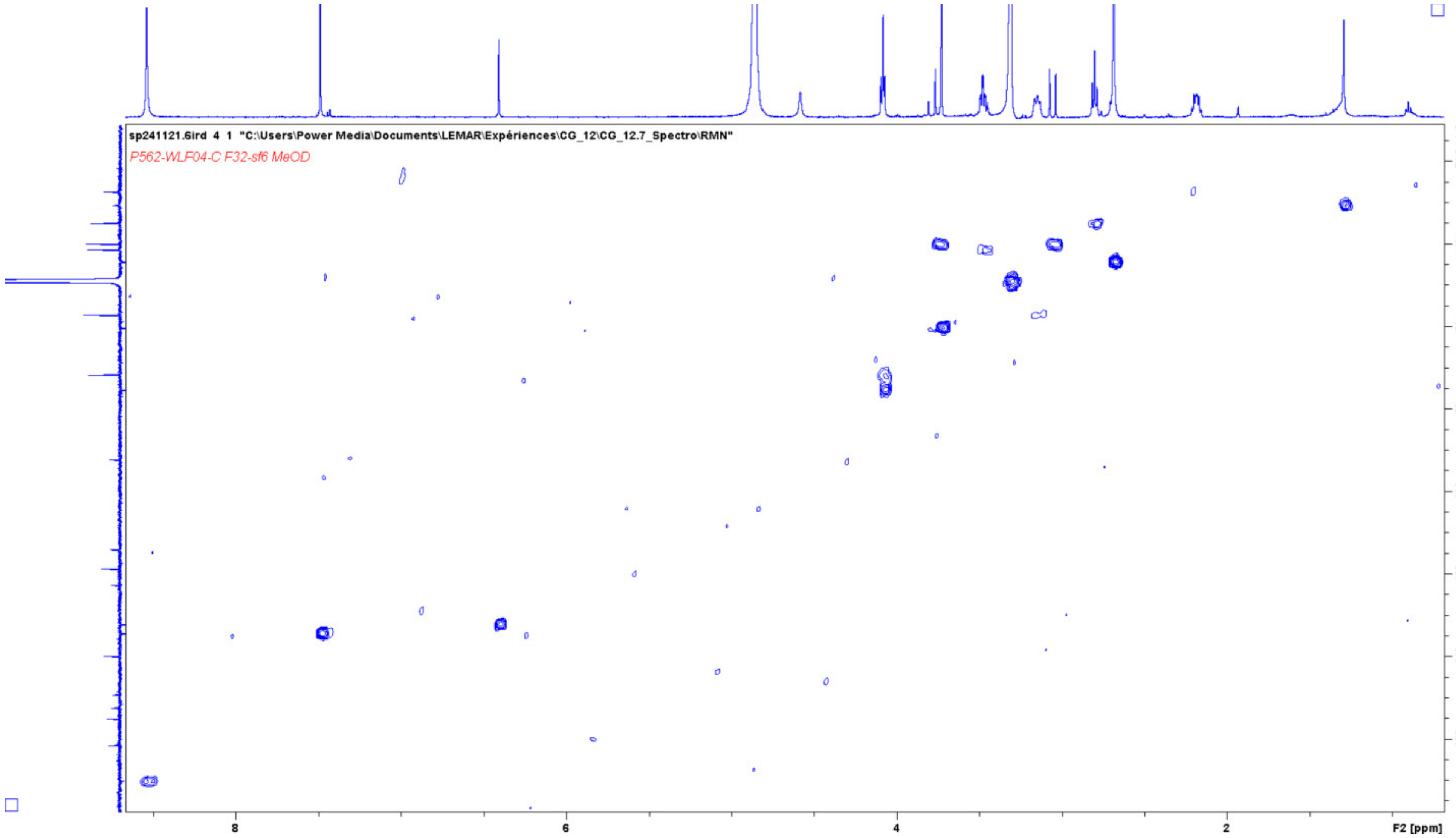

Figure S8. NMR HMQC ^{1}H-^{13}C in CD_3OD of compound **1**

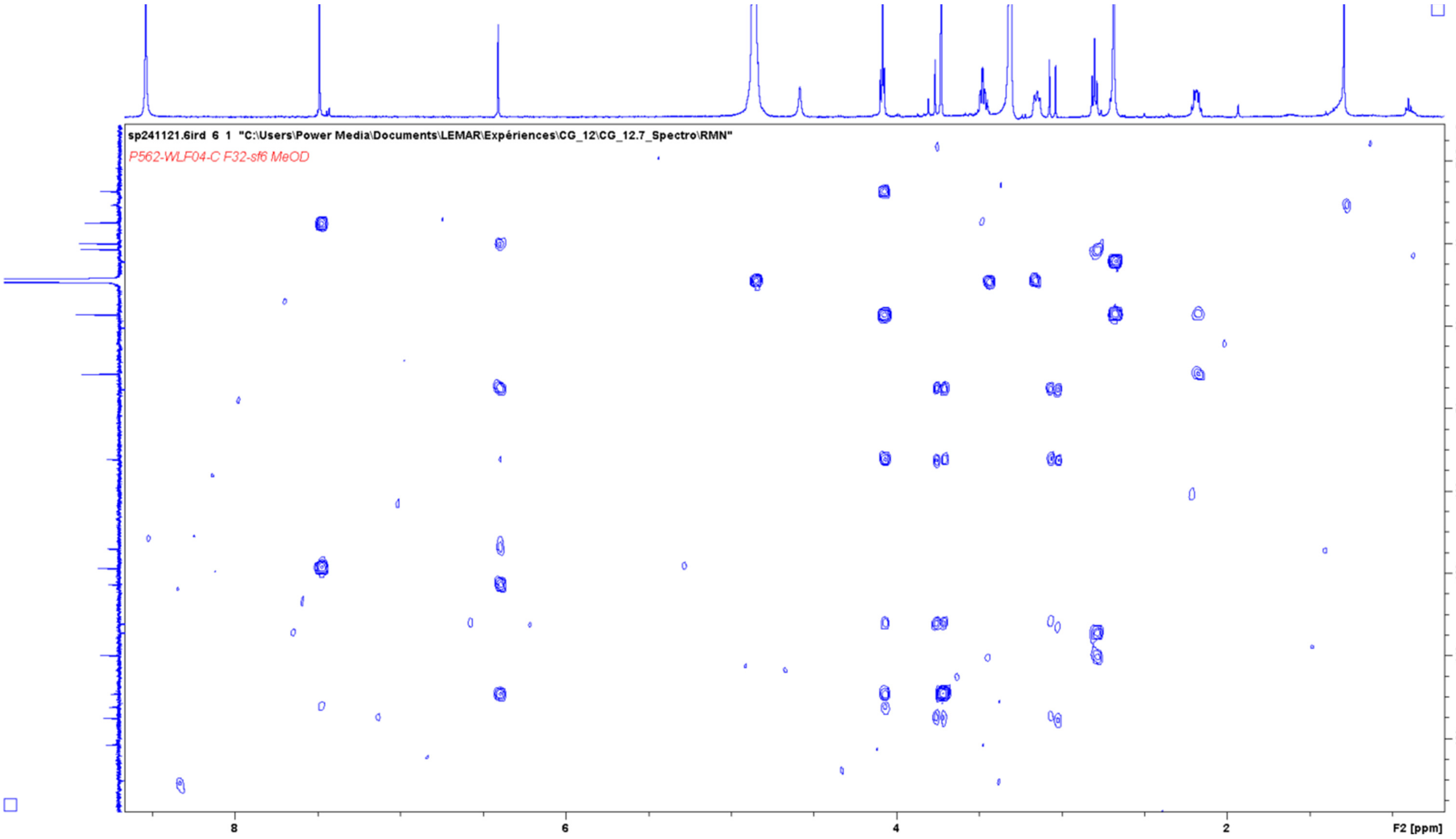

Figure S9. NMR HMBC ^1H-^{13}C in CD_3OD of compound **1**

Table S3. Comparison of the spectroscopic data of compound **2** with data from the literature concerning aplysamine-2.

	Compound **2**		Aplysamine-2 [60]	
		MS data		
	HRESIMS data showed the $[M+H]^+$ ion as a cluster at *m/z* 648, 650, 652, 654 in a 1:3:3:1 ratio, indicating the presence of three bromine atoms in the molecule. Analysis of the pic $[M+H]^+$ at *m/z* 647.9708 (calc. for $C_{23}H_{29}{}^{79}Br_3N_3O_4$, 647.9708) allowed us to propose the molecular formula of **2** as $C_{23}H_{28}N_3O_4Br_3$.		EIMS (15eV) $[M^+\text{-}HCl, 5\%]^+$ *m/z* 647, 649, 651, 653, calc. for $C_{23}H_{28}N_3O_4{}^{79}Br_3$, 646.9629, found 646.963.	
		1D NMR data		
Position	in CD_3OD		^{13}C in CD_3OD	
No	δ_C, type	δ_H mult, (*J* in Hz)	δ_C, type	δ_H mult, (*J* in Hz)
1	**131.8**, C		**113.1**	
2	134.8, CH	7.44, d (2.1)	134.7	7.43, d (2.2)
3	**112.2**, C		**130.3**	
4	155.9, C		155.8	
5	113.2, CH	6.89, d (8.5)	112.1	6.88, d (8.5)
6	130.3, CH	7.16, dd (8.5; 2.15)	131.7	7.17, dd (8.5; 2.2)
7	28.7, CH_2	3.81, s	28.7	3.79, s
8	153.0, C		152.9	
9	165.8, C		165.8	
10	41.3, CH_2	3.43, t (7.0)	41.3	3.41, t (7.0)
11	35.1, CH_2	2.74, t (7.0)	35.2	2.73, t (7.0)
12	139.8, C		140.3	
13	134.4, CH	7.41, s	134.4	7.42, s
14	118.9, C		118.7	
15	152.8, C		152.1	
16	72.5, CH_2	4.0, t (6.1)	71.7	4.05, t (5.5)
17	28.8, CH_2	2.04, tt (7.7; 6.1)	26.4	2.25, tt (7.6; 5.5)
18	57.5, CH_2	2.69 t (7.9)	56.9	3.46, t (7.6)
$N(Me)_2$	45.3, CH_3	2.33, s	43.7	2.93, s
OMe	56.7, CH_3	3.83, s	56.7	3.81, s

In-depth study of the HMBC spectrum raises questions about the allocation of the signals relating to the quaternary carbons C-1 and C-3 in the literature [15, 47]. The first signal at 112.2 ppm is paired with H-5, while the second at 131.8 ppm is paired with H-5 and H-7 (Figure S10). On this basis, we propose the assignment given in Table S3. This attribution is further supported by the chemical shifts of C-14 (δ = 118.9 ppm) and C-12 (δ = 139.3 ppm), potential "analogues" respectively of C-3, an aromatic carbon bonded to a bromine atom, and C-1, an aromatic carbon bonded to a secondary carbon, in ortho position of an oxygen.

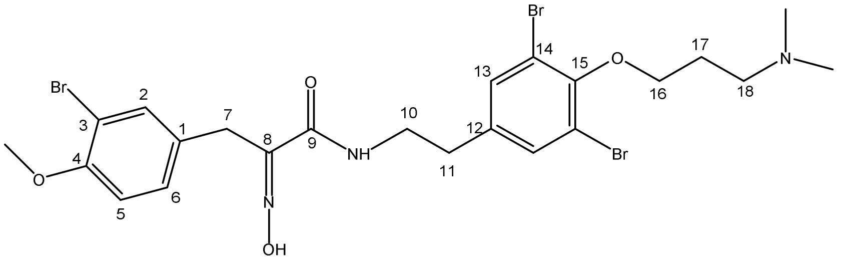

Figure S10. Formula for compound **2** corresponding to aplysamine-2

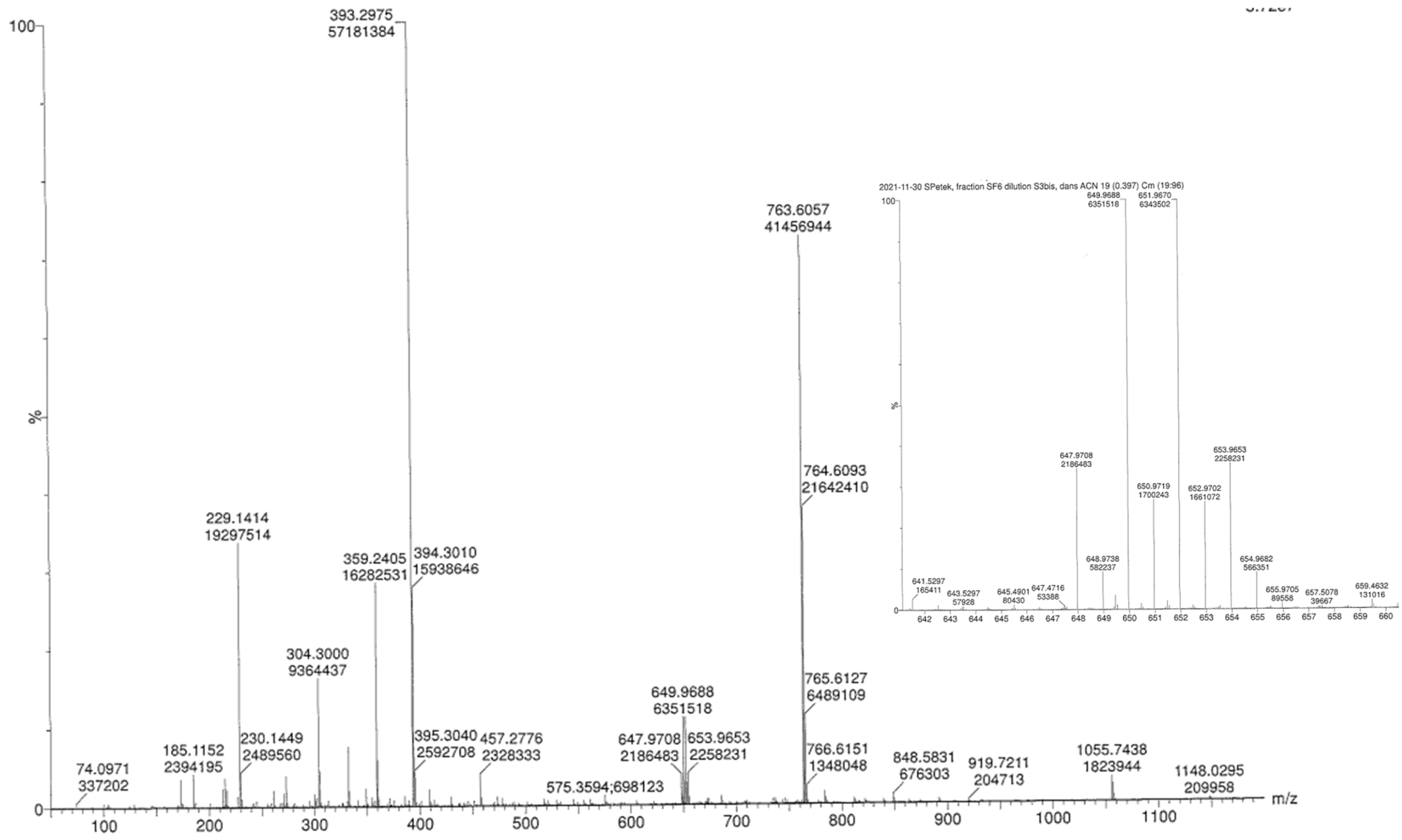

Figure S11. HR ESI spectrum in ACN compound 2

of compound **2**

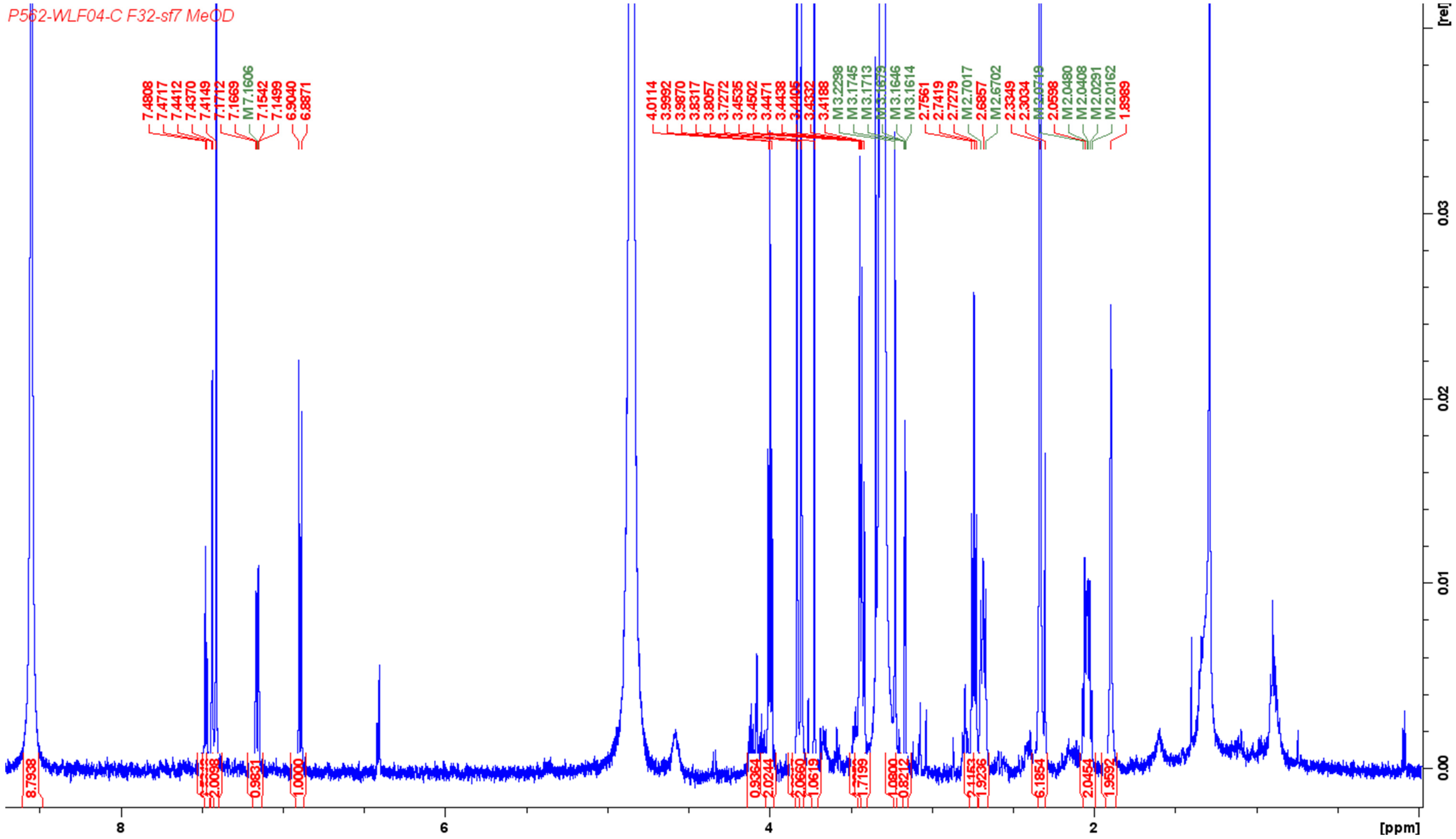

Figure S12. NMR ^{1}H in CD_3OD of compound **2**

16

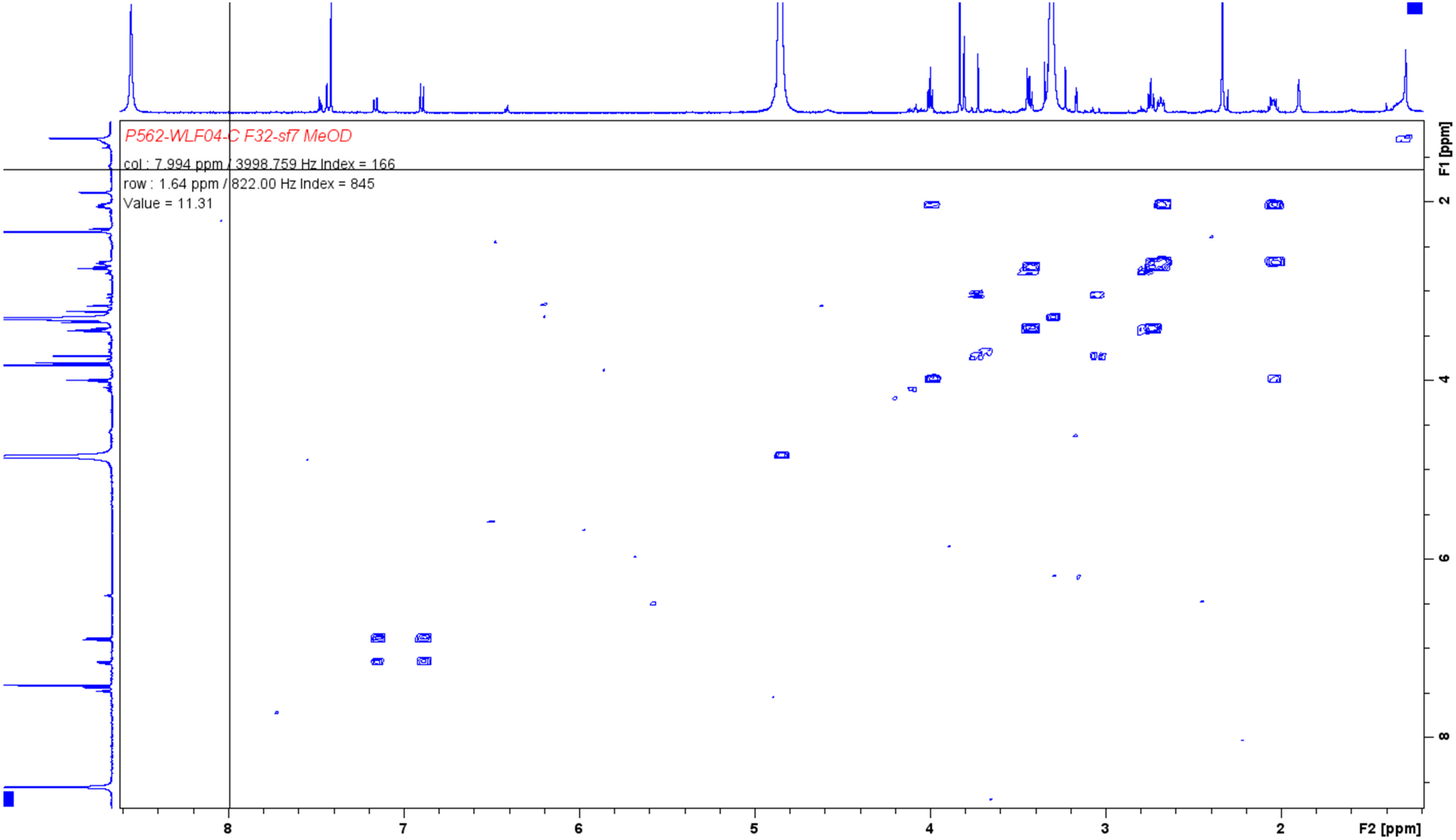

17

Figure S13. NMR COSY ^{1}H-^{1}H in CD_3OD of compound **2** 18

19

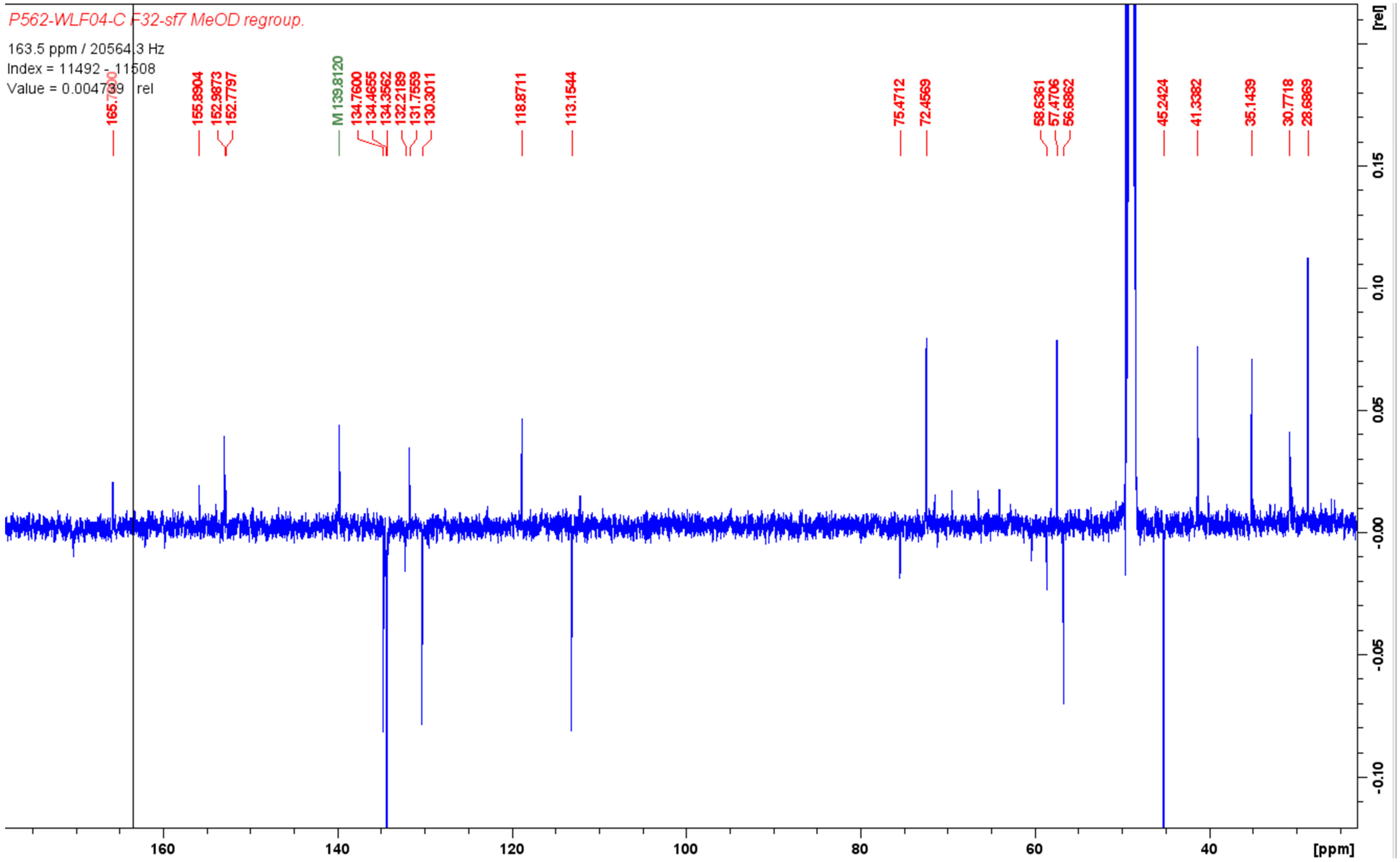

Figure S14. NMR JMOD ^{13}C in CD_3OD of compound **2**

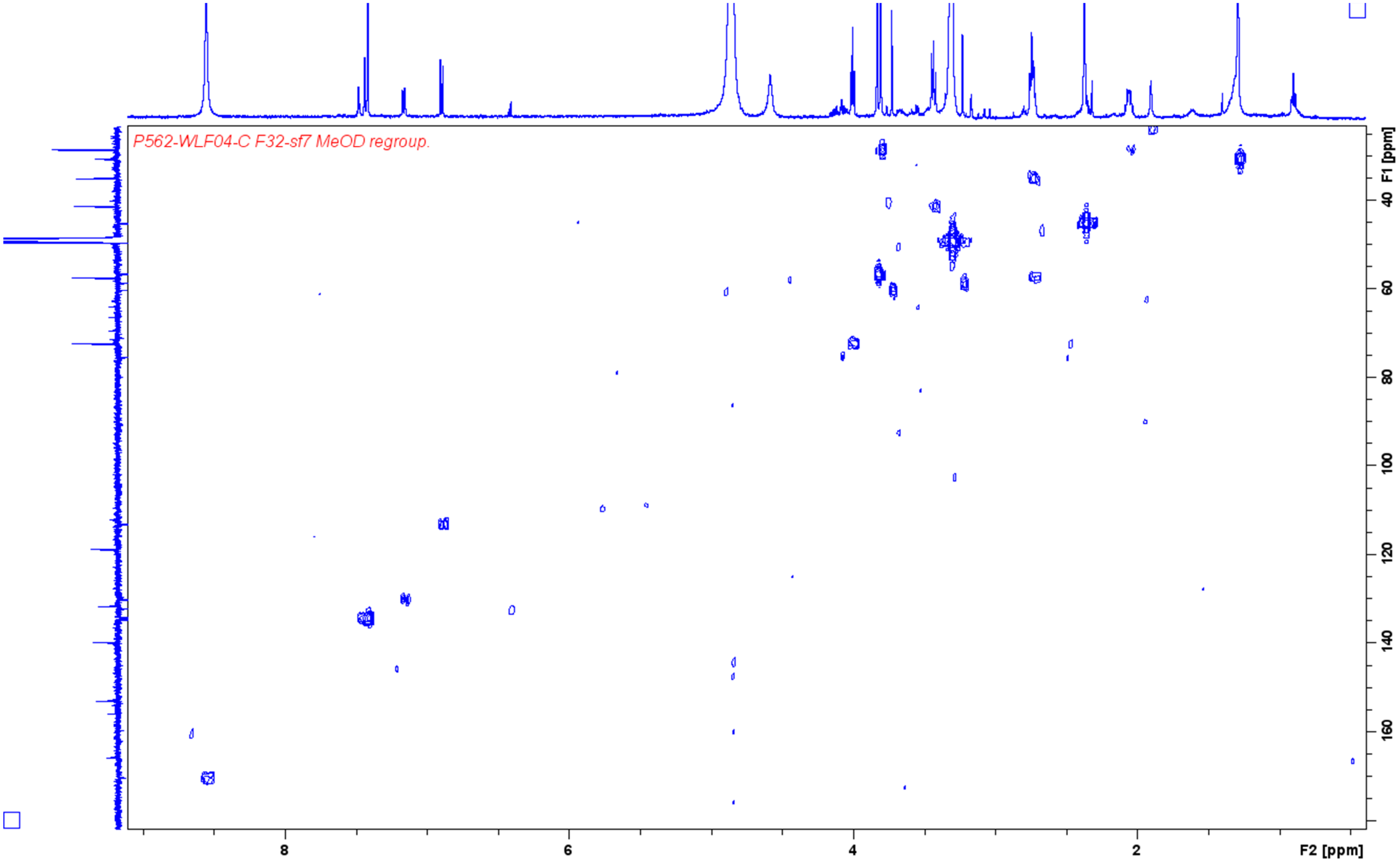

22

Figure S15. NMR HMQC ^{1}H-^{13}C in CD_3OD of compound **2** 23

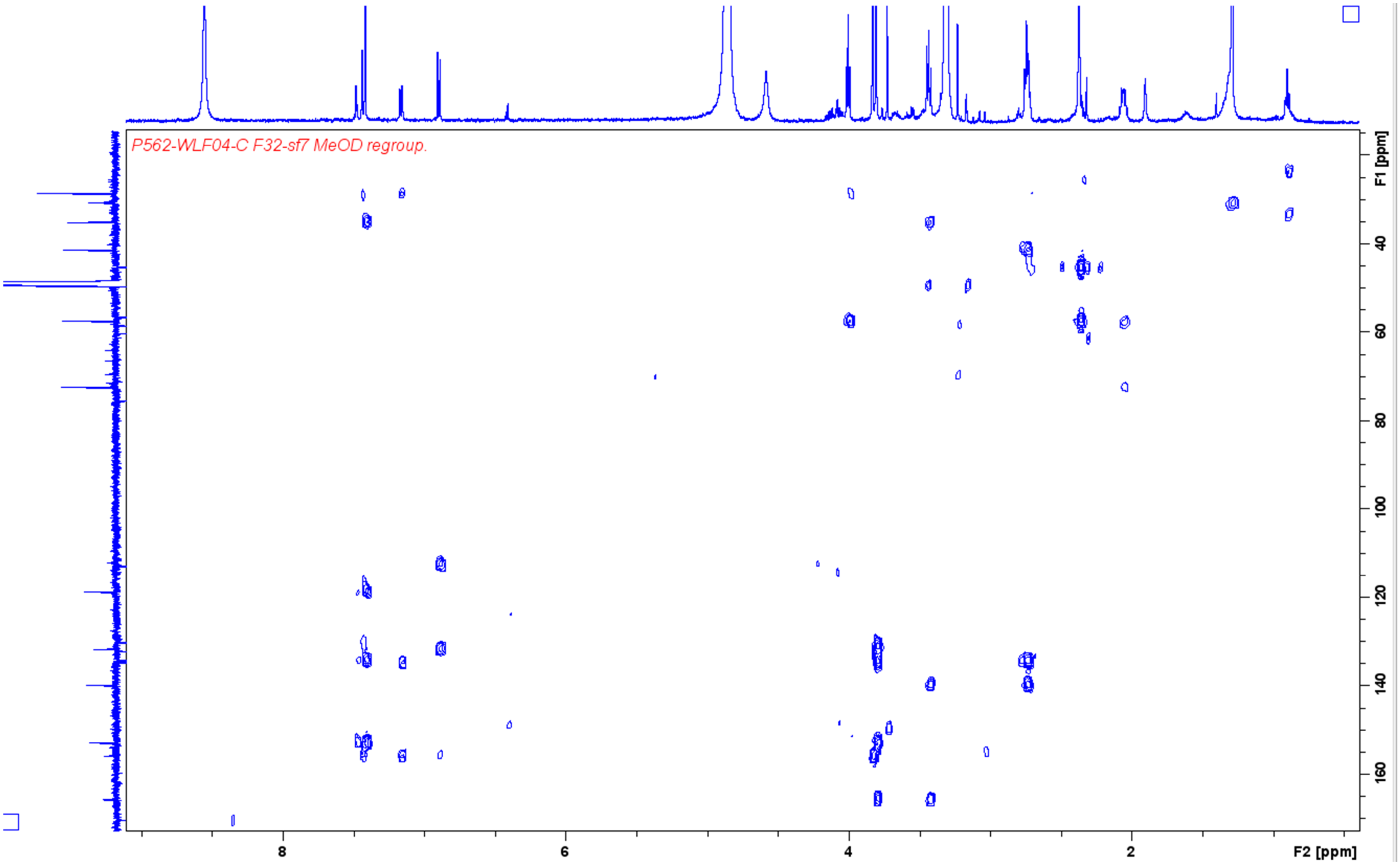

Figure S16. NMR HMBC ^{1}H-^{13}C in CD_3OD of compound **2**

Table S4. Comparison of the spectroscopic data of compound **3** with data from the literature concerning pseudoceratinine A.

	Compound **3**		Pseudoceratinine A [61]	
	**MS data**			
	HRESIMS data showed the $[M+H]^+$ ion as a cluster at *m/z* 490, 492, 494 in a 1:2:1 ratio, indicating the presence of two bromine atoms in the molecule. Analysis of the pic $[M+H]^+$ at *m/z* 489.9659 (calc. for $C_{15}H_{18}N_5O_4{}^{79}Br_2$, 489.9725) allowed us to propose the molecular formula of **3** as $C_{15}H_{17}N_5O_4Br_2$.		FABMS $[M+H]^+$ *m/z* 490, 492, 494.	
	**1D NMR data**			
Position	in CD_3OD		^{13}C in CD_3OD	
No	δ_C, type	δ_H mult, (*J* in Hz)	δ_C, type	δ_H mult, (*J* in Hz)
1	75.5 , CH	4.08, s	75.6	4.08, s
2	122.8, C		123.1	
3	149.3, C		149,0	
4	114.1, C		114.5	
5	132.2 , CH	6.41, s	132.6	6.40, s
6	92.4 , C		92.9	
7	40.1 , CH_2	3.09, d (18.3)	39.5	3.19, d (18.6)
7'		3.78, d (18.3)		3.61, d (18.6)
8	155.2 , C		155.6	
9	161.7 , C		162.1	
10	39.3 , CH_2	3.5, t (6.9)	40.5	3.51, t (7.0)
11	26.1 , CH_2	2.71, t (6.9)	26.1	2.76, t (7.0)
12	127.1 , C		126.3	
13	111.1 , CH	6.51, s	111.3	6.55, s
14	149.3 , C		149.7	
OMe	60.4 , CH_3	3.73, s	60.8	3.72, s

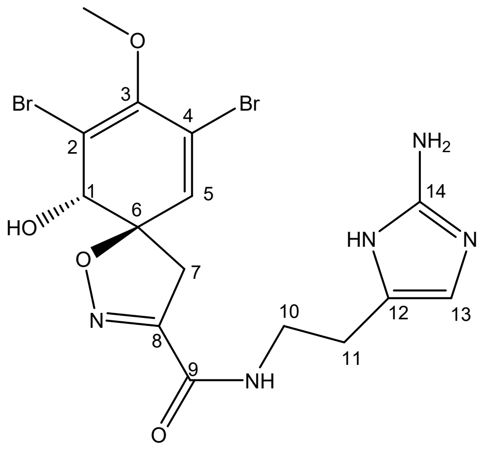

Figure S17. Formula for compound **3** corresponding to pseudoceratinine A

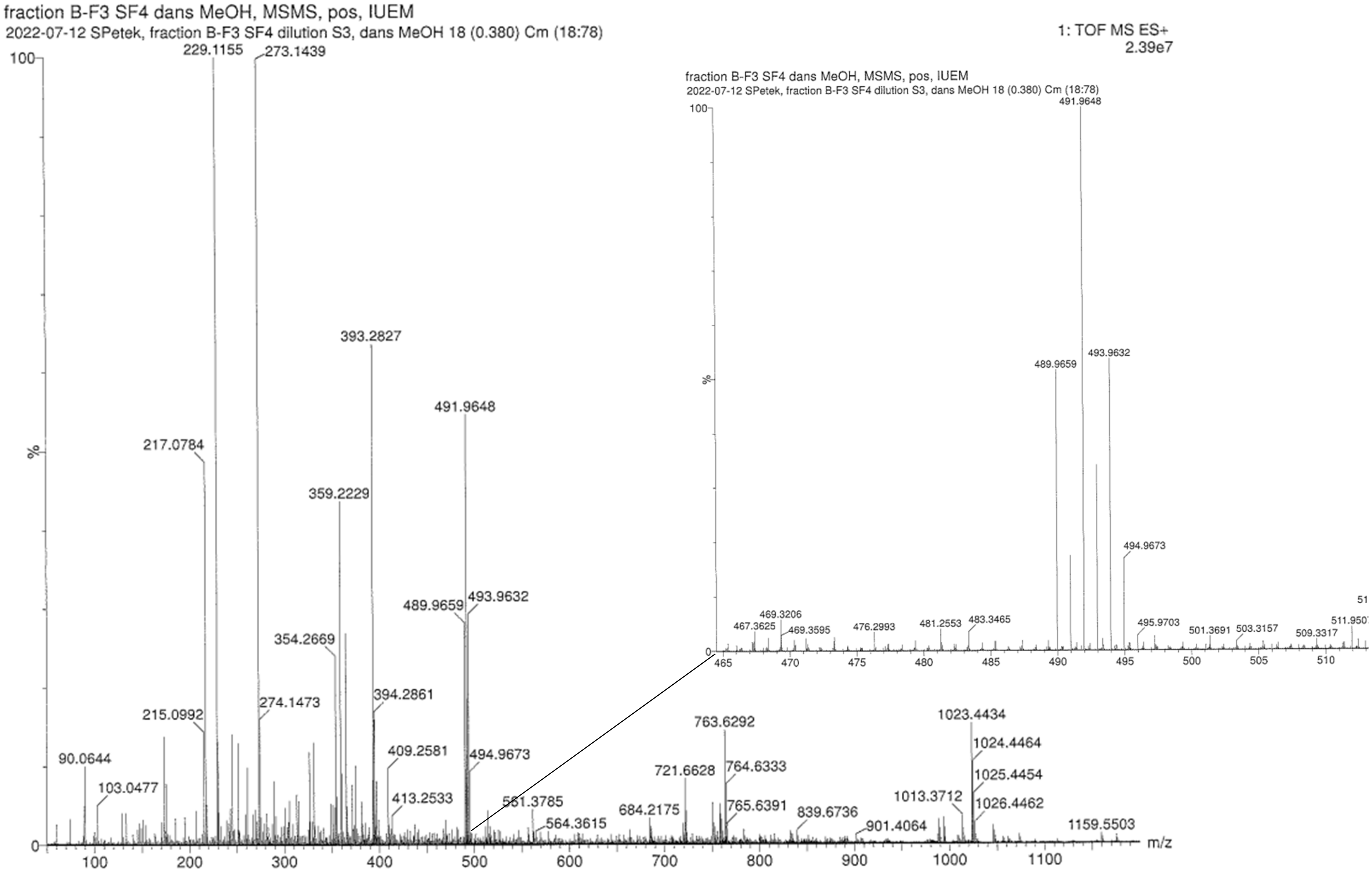

Figure S18. HRESIMS of compound **3**

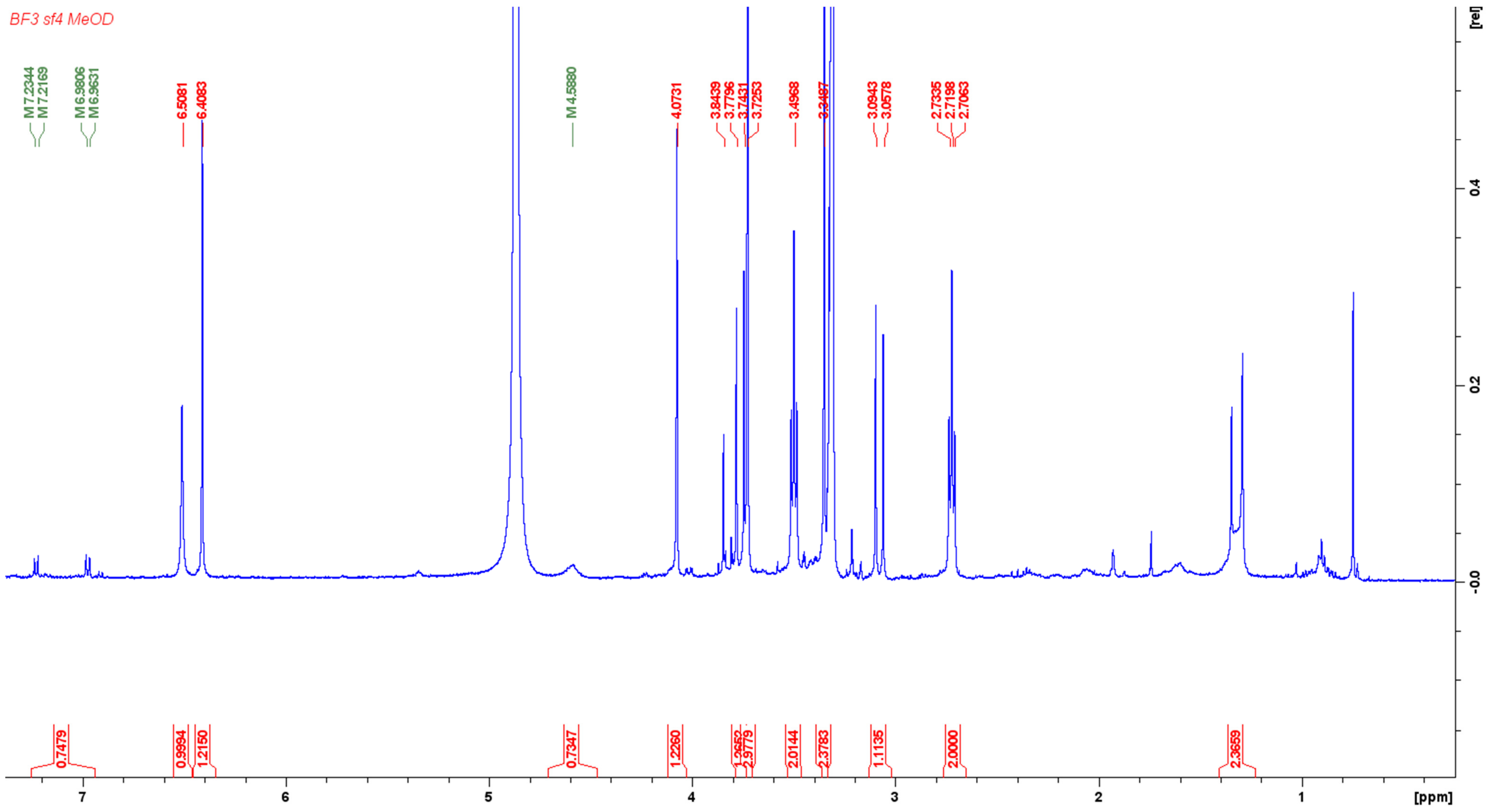

Figure S19. NMR ^{1}H in CD_3OD of compound **3**

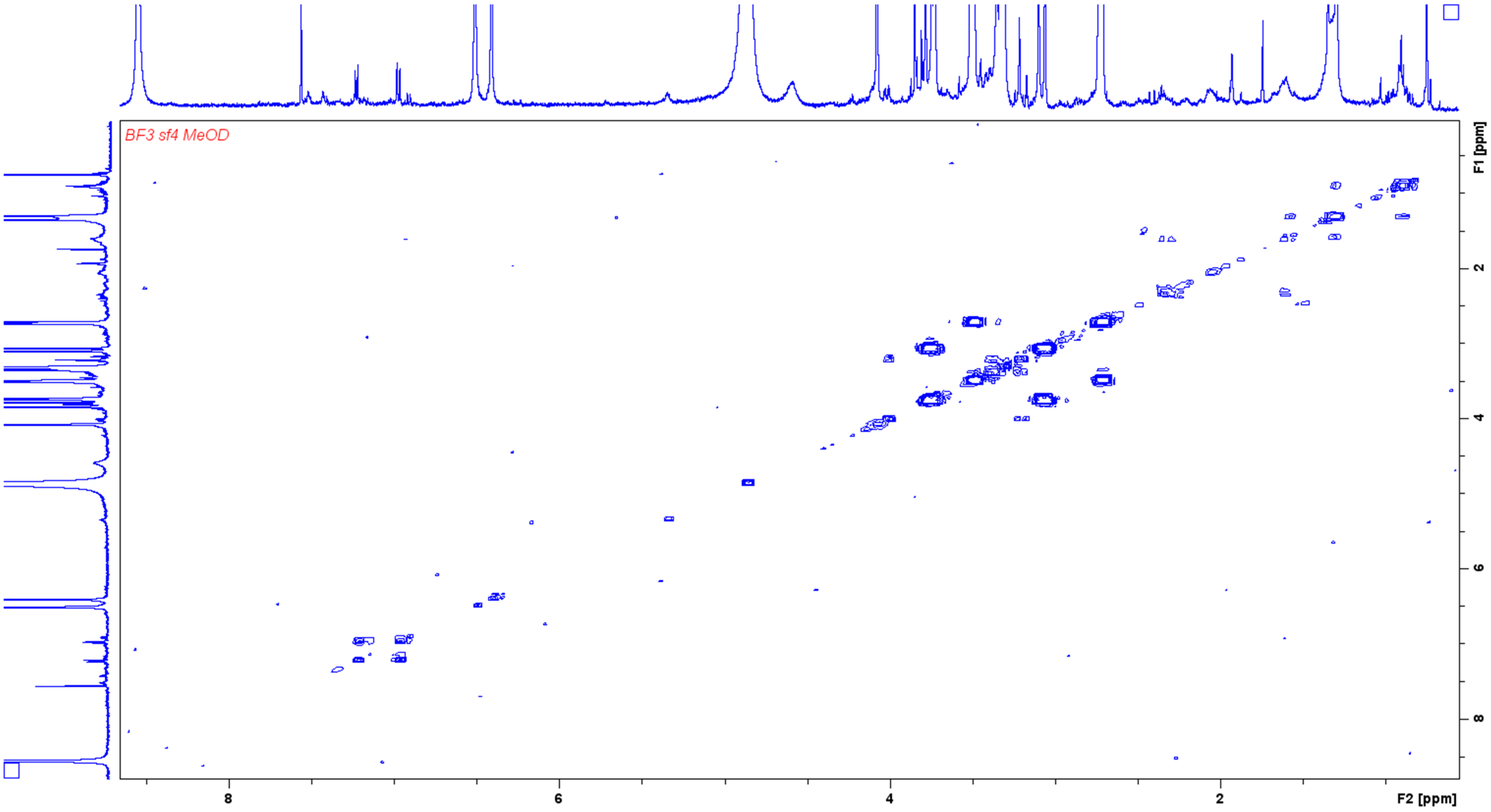

Figure S20. NMR COSY ^{1}H-^{1}H in CD_3OD of compound **3**

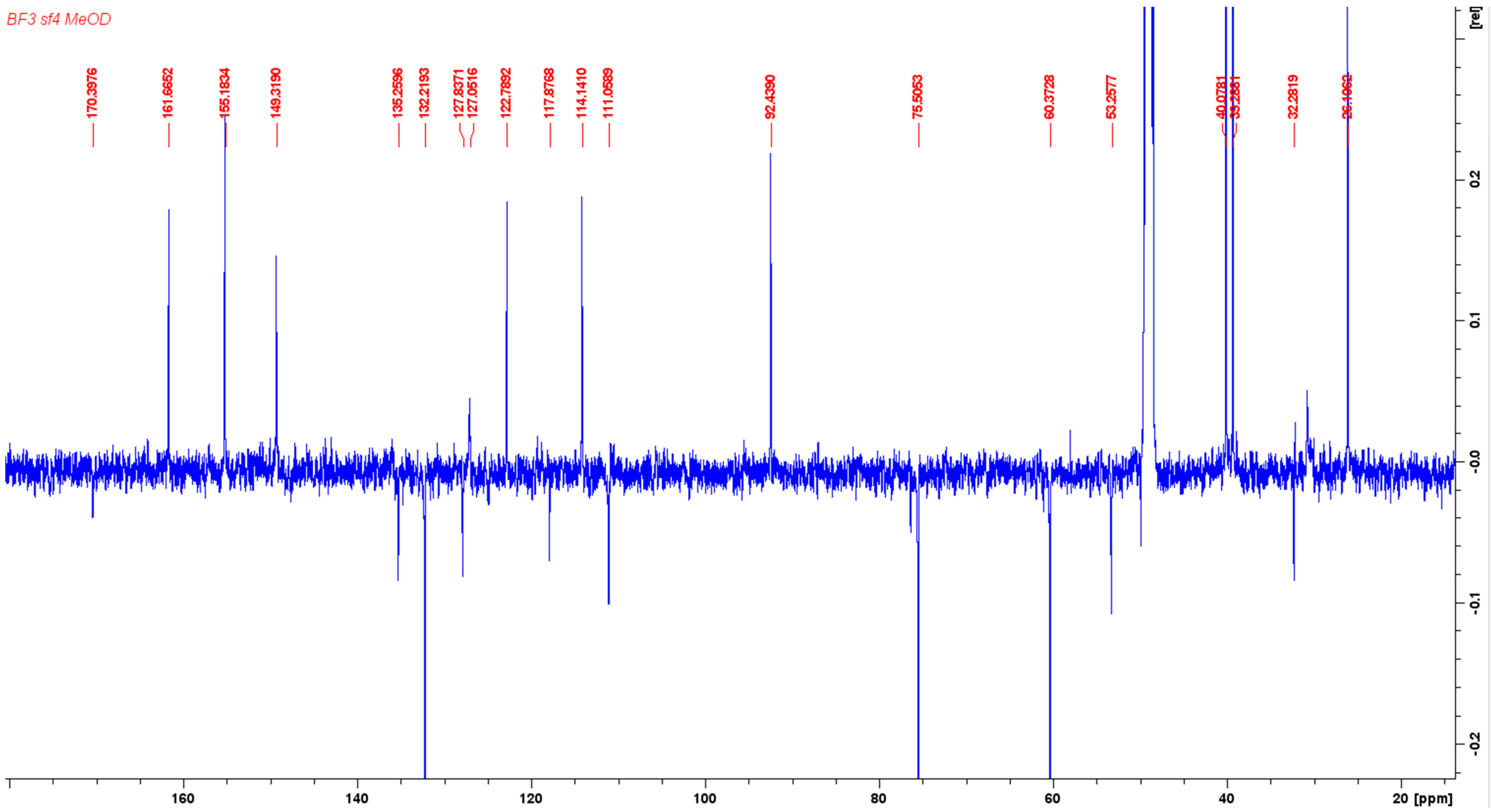

Figure S21. NMR JMOD ^{13}C in CD_3OD of compound **3**

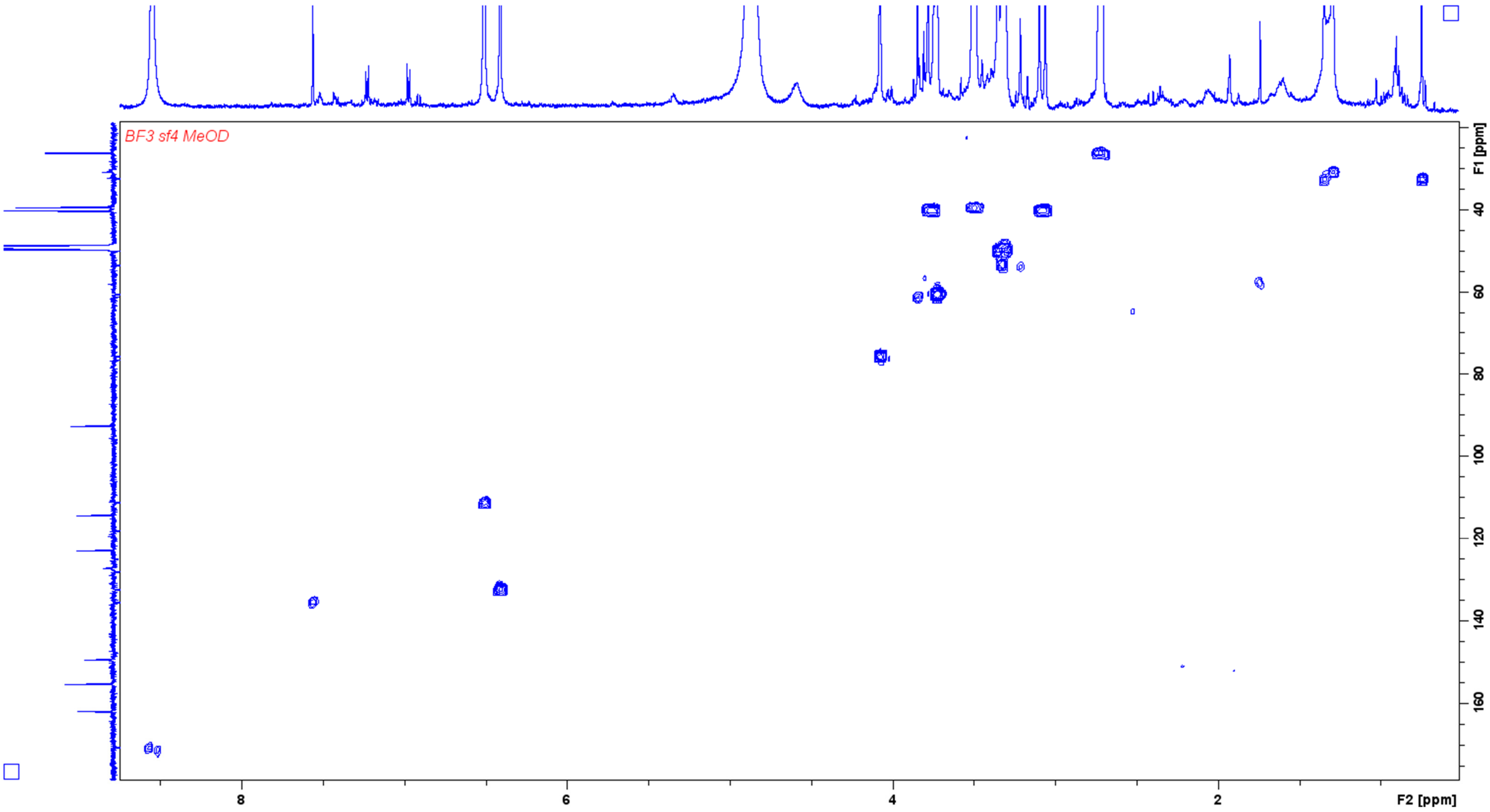

Figure S22. NMR HMQC ^{1}H-^{13}C in CD_3OD of compound **3**

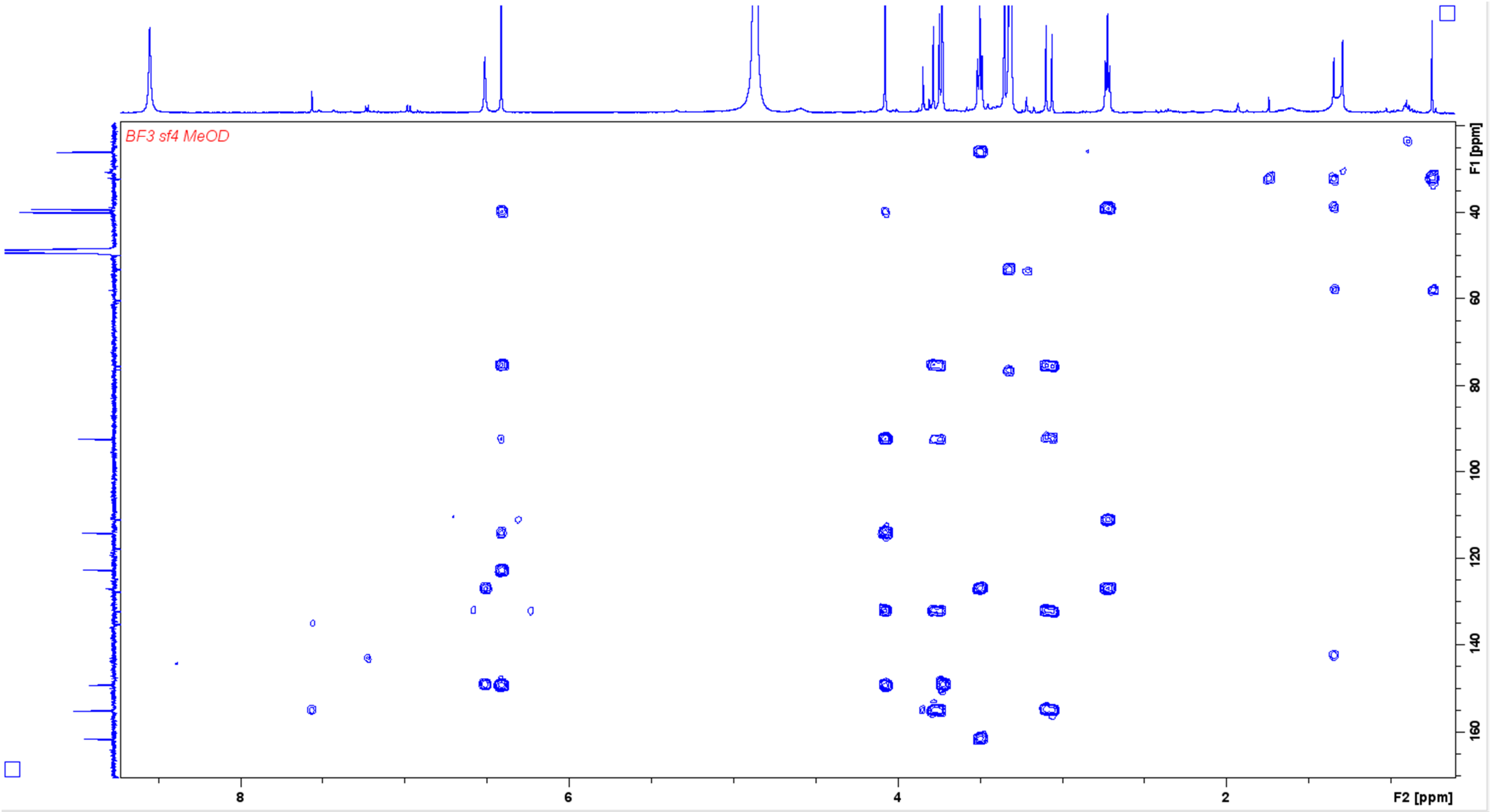

Figure S23. NMR HMBC ^{1}H-^{13}C in CD_3OD of compound **3**

Table S6. Comparison of the spectroscopic data of compound **4** with data from the literature concerning aerophobin-2.

	Compound **4**		Aerophobin-2 [62]	
	MS data			
	HRESIMS data showed the $[M+H]^+$ ion as a cluster at *m/z* 504, 506, 508 in a 1:2:1 ratio, indicating the presence of two bromine atoms in the molecule. Analysis of the pic $[M+H]^+$ at *m/z* 503.9829 (calc. for $C_{16}H_{20}N_5O_4{}^{79}Br_2$, 503.9882) allowed us to propose the molecular formula of **4** as $C_{16}H_{19}N_5O_4Br_2$.		ESIMS $[M+H]^+$ *m/z* 504, 506, 508 in a 1:2:1 ratio.	
	1D NMR data			
Position	in CD_3OD		in DMSO-d_6	
No	δ_C, type	δ_H mult, (*J* in Hz)	δ_C	δ_H mult, (*J* in Hz)
1	75.5, CH	4.08, s	74.1	3.90, s
2	122.8, C		121.3	
3	149.3, C		147.6	
4	114.2, C		113.6	
5	132.2, CH	6.42, s	131.7	6.56, s
6	92.4, C		90.7	
7	40.1, CH_2	3.09, d (18.3)	39.8	3.18, d (17.8)
7'		3.78, d (18.3)		3.60, d (17.8)
8	155.2, C		155.0	
9	161.7, C		159.5	
10	39.5, CH_2	3.32	38.5	3.16, q (6.6)
11	29.2, CH_2	1.84, m	27.8	1.69, m
12	23.3, CH_2	2.52, t (7.1)	22.0	2.39, t (7.4)
13	129.3, C		126.8	
14	110.3, CH	6.48, s	109.3	6.57, s
15	149.0, C		147.3	
OMe	60.4, CH_3	3.73, s	60.1	3.63, s

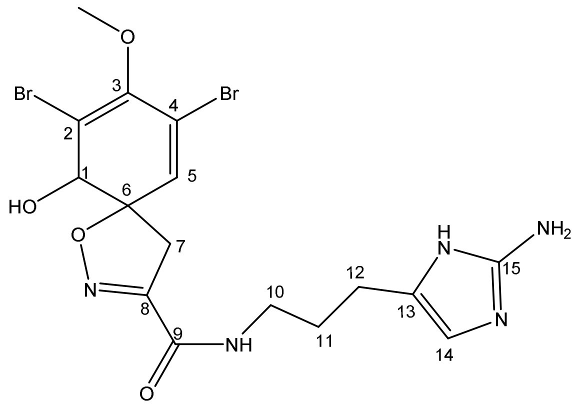

Figure S24. Formula for compound **4** corresponding to aerophobin-2

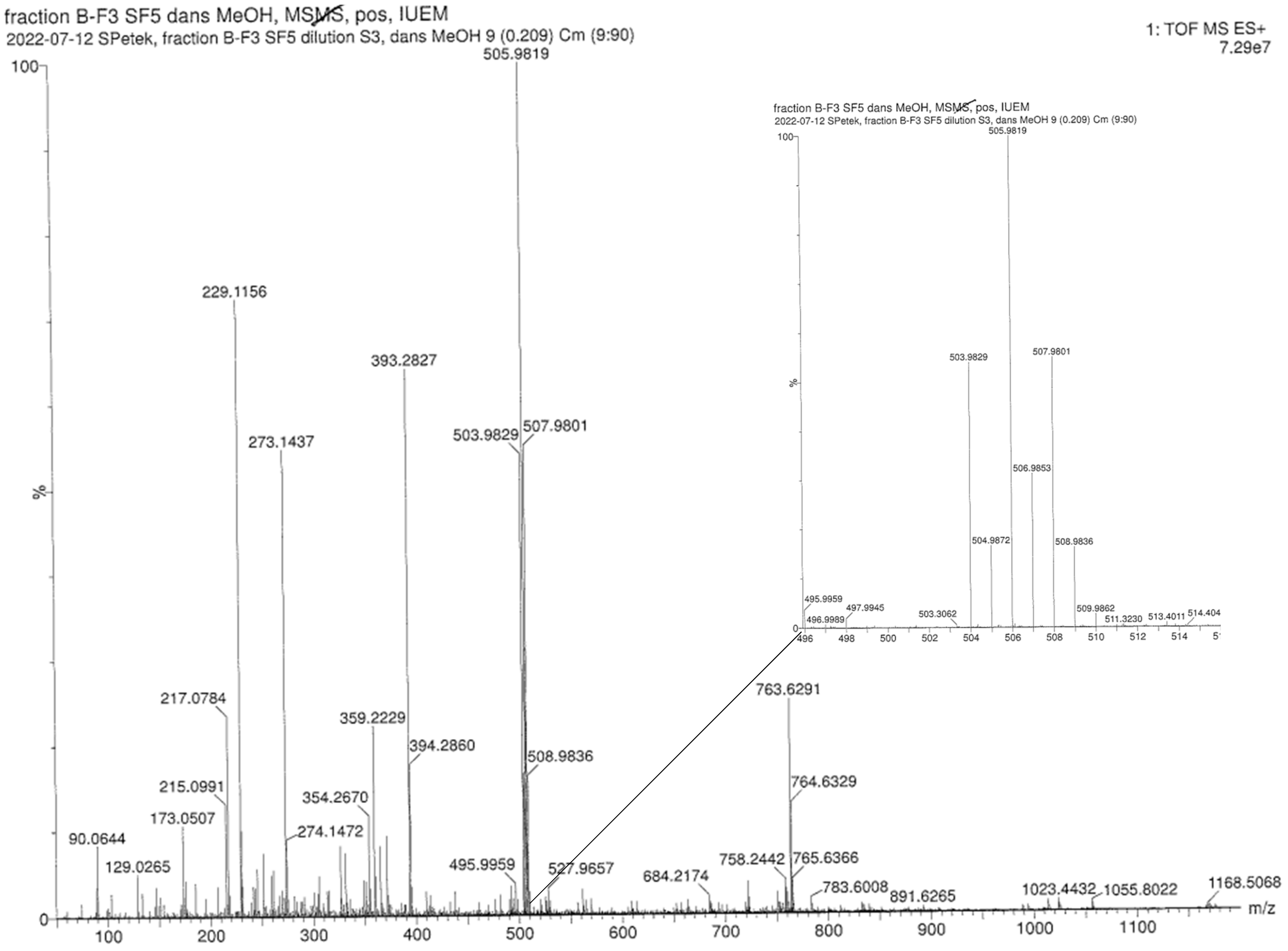

1

Figure S25. HRESIMS of compound **4**

2

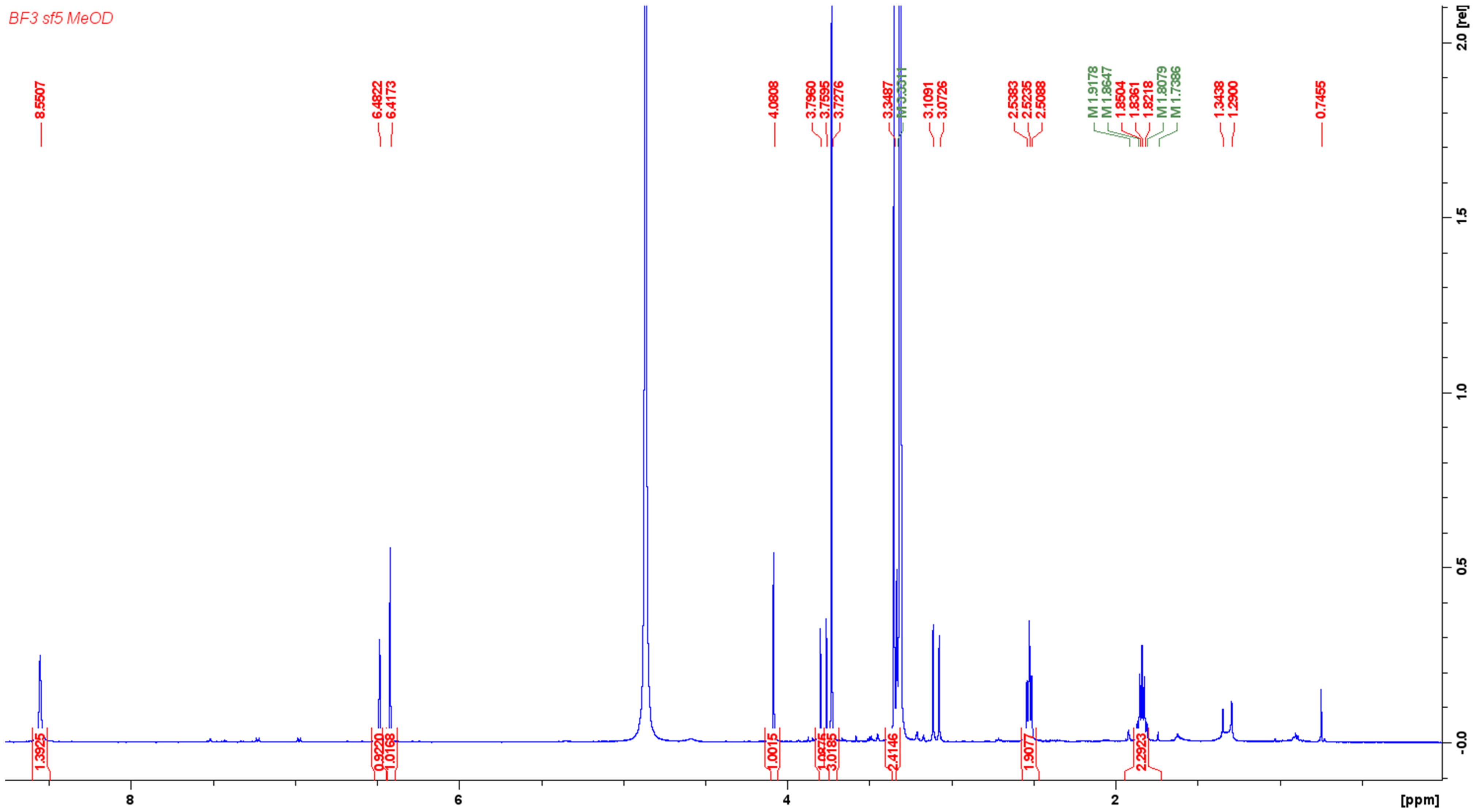

Figure S26. NMR ^1H in CD_3OD of compound **4**

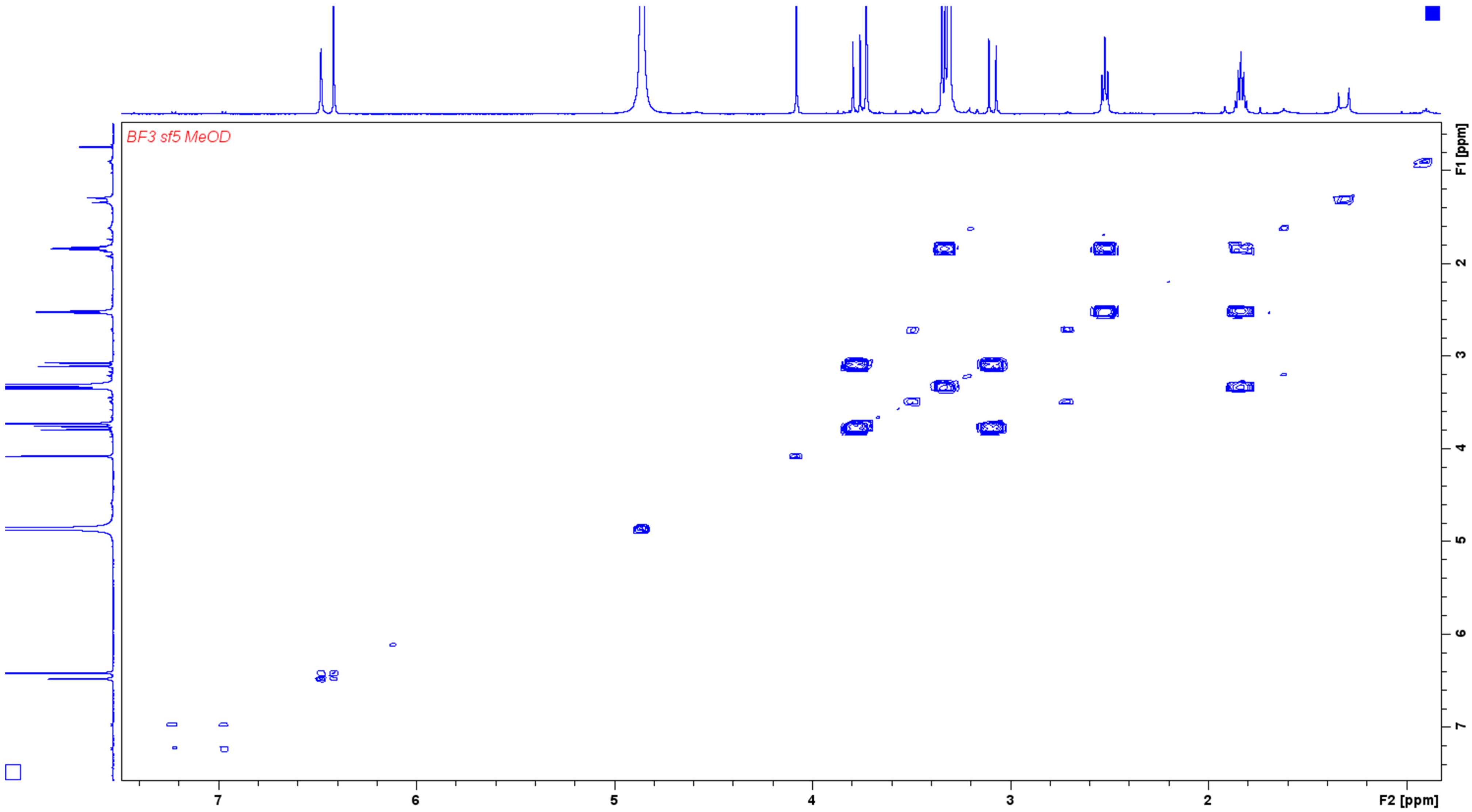

Figure S27. NMR COSY ^{1}H-^{1}H in CD_3OD of compound **4**

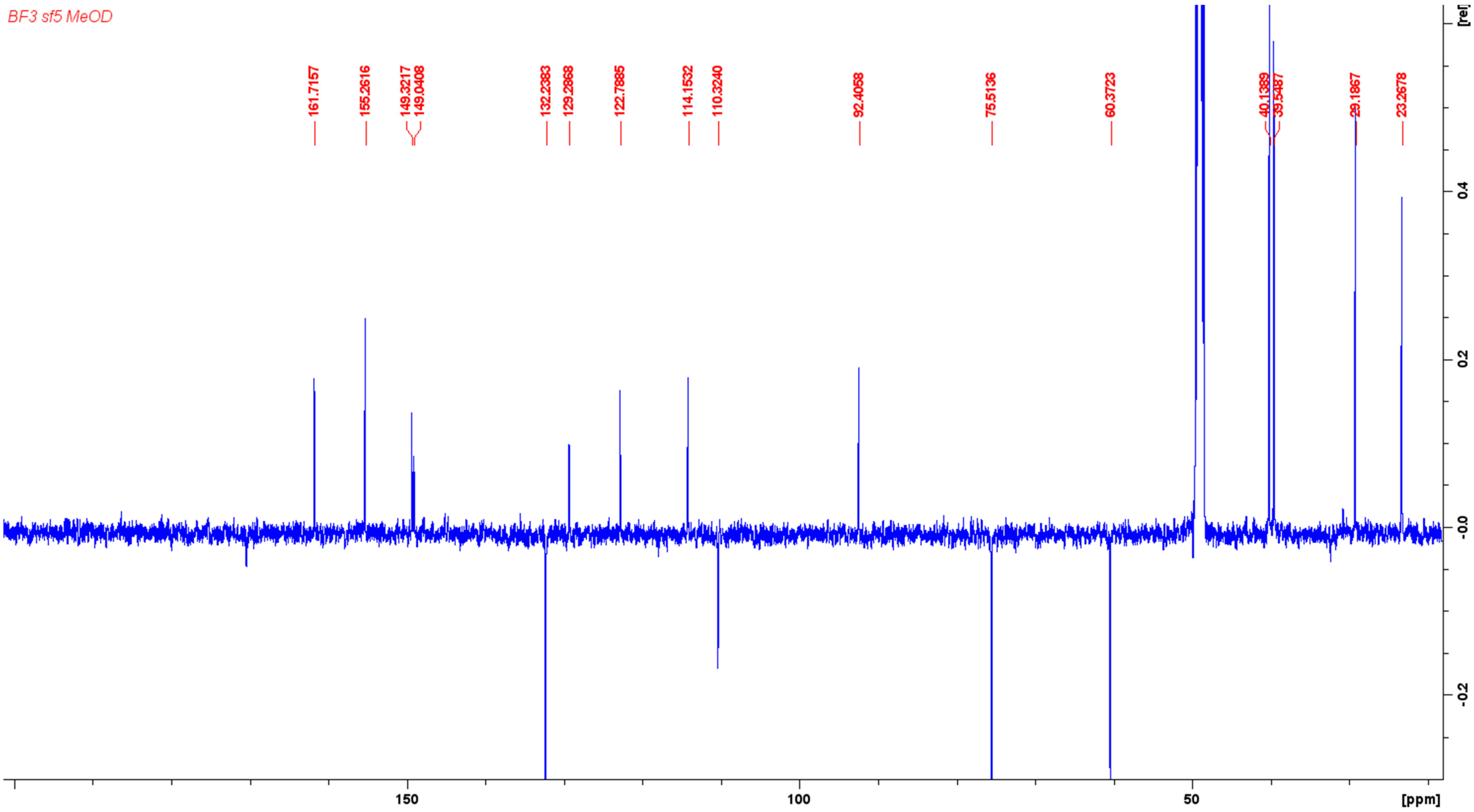

Figure S28. NMR JMOD ^{13}C in CD_3OD of compound **4**

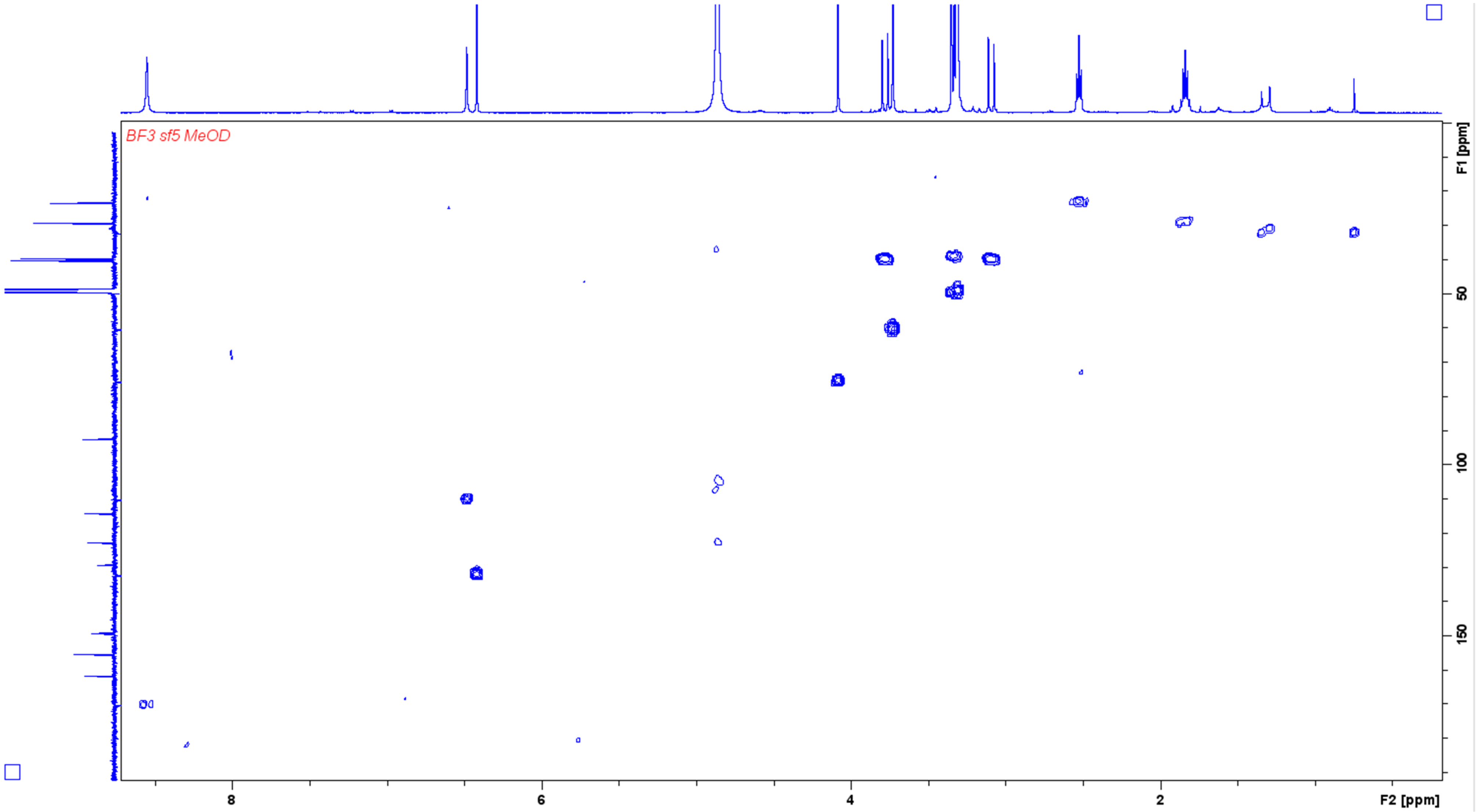

Figure S29. NMR HMQC ^{1}H-^{13}C in CD_3OD of compound **4**

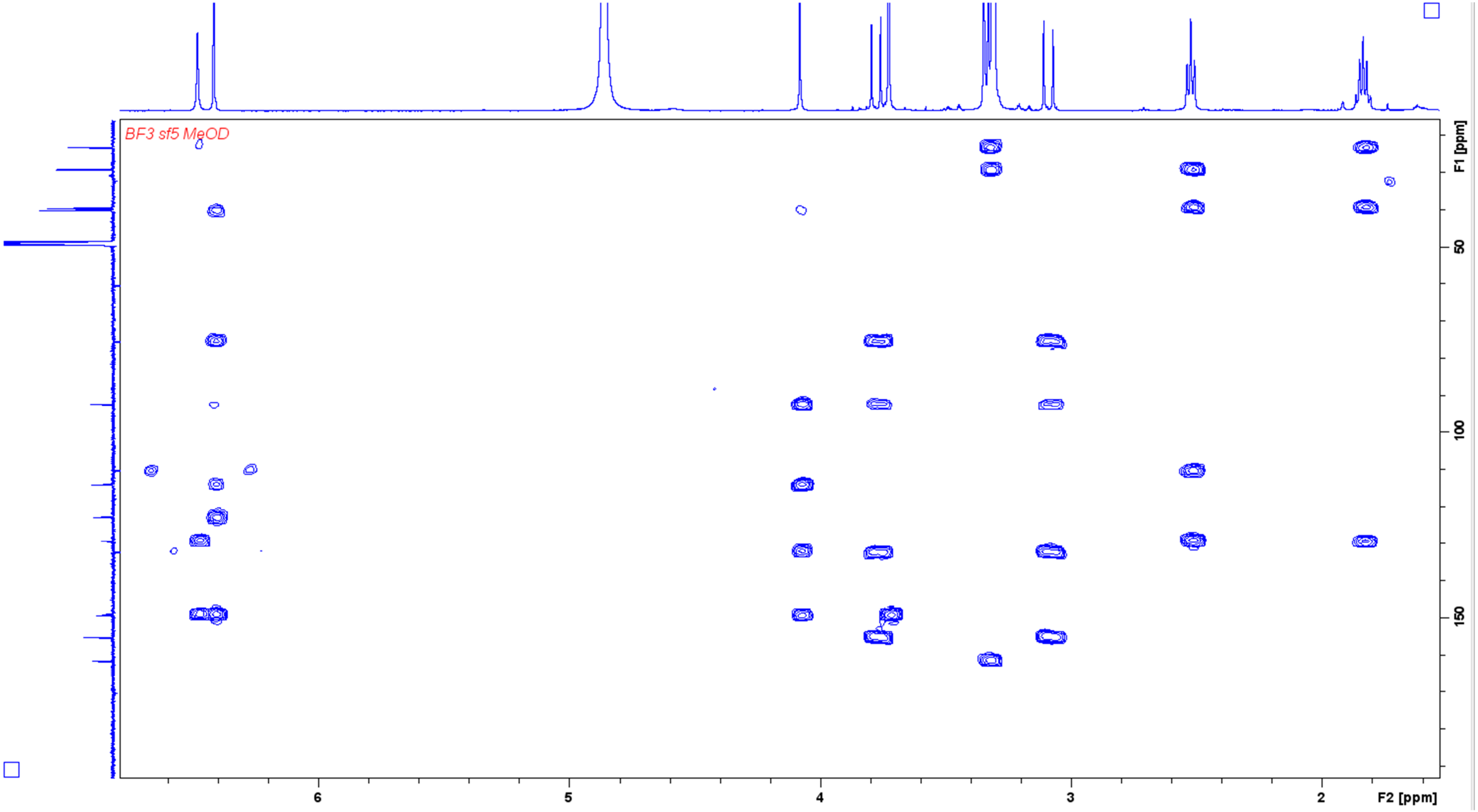

Figure S30. NMR HMBC ^{1}H-^{13}C in CD_3OD of compound **4**

Table S6. Comparison of the spectroscopic data of compound **5** with data from the literature concerning aplysamine-1.

Compound **5**			aplysamine-1 [60]	
MS data				
HRESIMS data showed the $[M+H]^+$ ion as a cluster at *m/z* 407, 409, 411 in a 1:2:1 ratio, indicating the presence of two bromine atoms in the molecule. Analysis of the pic $[M+H]^+$ at *m/z* 407.0328 (calc. for $C_{15}H_{25}N_2OBr_2$, 407.0334) allowed us to propose the molecular formula of **5** as $C_{15}H_{24}ON_2Br_2$.			ESIMS $[M+H]^+$ *m/z* 504, 506, 508 in a 1:2:1 ratio.	
1D NMR data				
Position	in CD_3OD		in CD_3OD	
No	δ_C, type	δ_H mult, (*J* in Hz)	δ_C	δ_H mult, (*J* in Hz)
1	152.8, C		152.1, C	
2	118.9, C		118.7, C	
3	134.1, CH	7.50, s	134.4, CH	7.62, s
4	140.5, C		140.3, C	
5	33.1, CH_2	2.71, t, (8.0)	35.2, CH_2	3.02, t, (8,0)
6	61.6, CH_2	2.56, t, (8.0)	41.3, CH_2	3.50, t, (8.0)
7, 7′	45.2, CH_3	2.30, s	43.7, CH_3	2.91, s
8	72.5, CH_2	4.02, t, (6.0)	71.7, CH_2	4.12, t, (5.5)
9	28.7, CH_2	2.05, tt, (6.0, 8.0)	26.4, CH_2	2.30, tt, (5.5, 5.5)
10	57.4, CH_2	2.73, t, (8.0)	56.9, CH_2	3.22, t, (5.5)
11, 11′	45.2, CH_3	2.34, s	43.6, CH_3	2.96, s

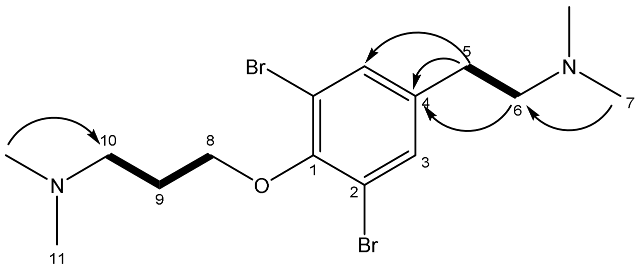

Figure S31. Formula for compound **5** corresponding to aplysamine-1

Depth study of the HMBC, COSY and HSQC spectrum raises questions about the allocation of the signal relating to H-5, H-6, and C-6 in the literature [60], and our analysis leads us to propose these new attributions.

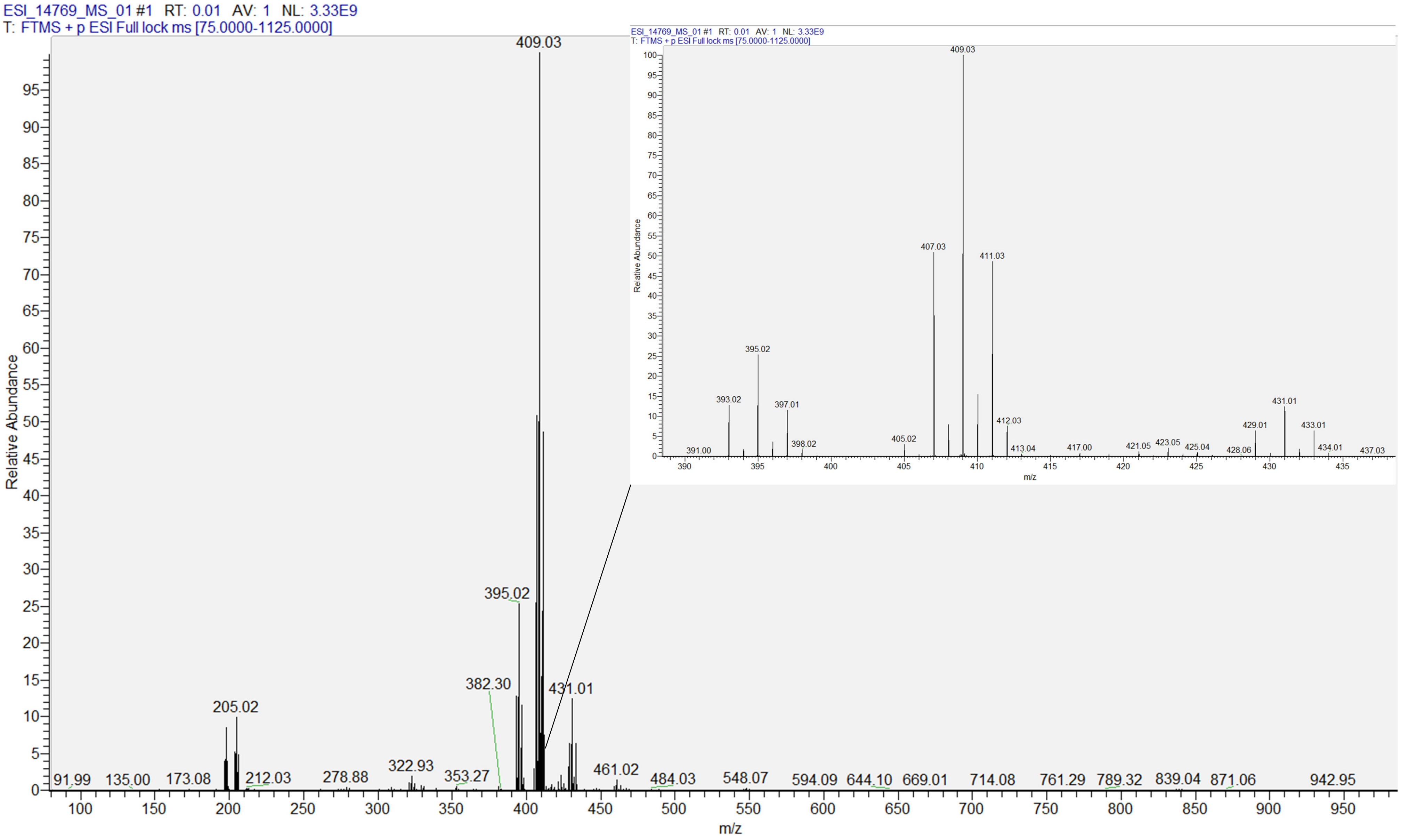

Figure S32. HRESIMS of compound **5**

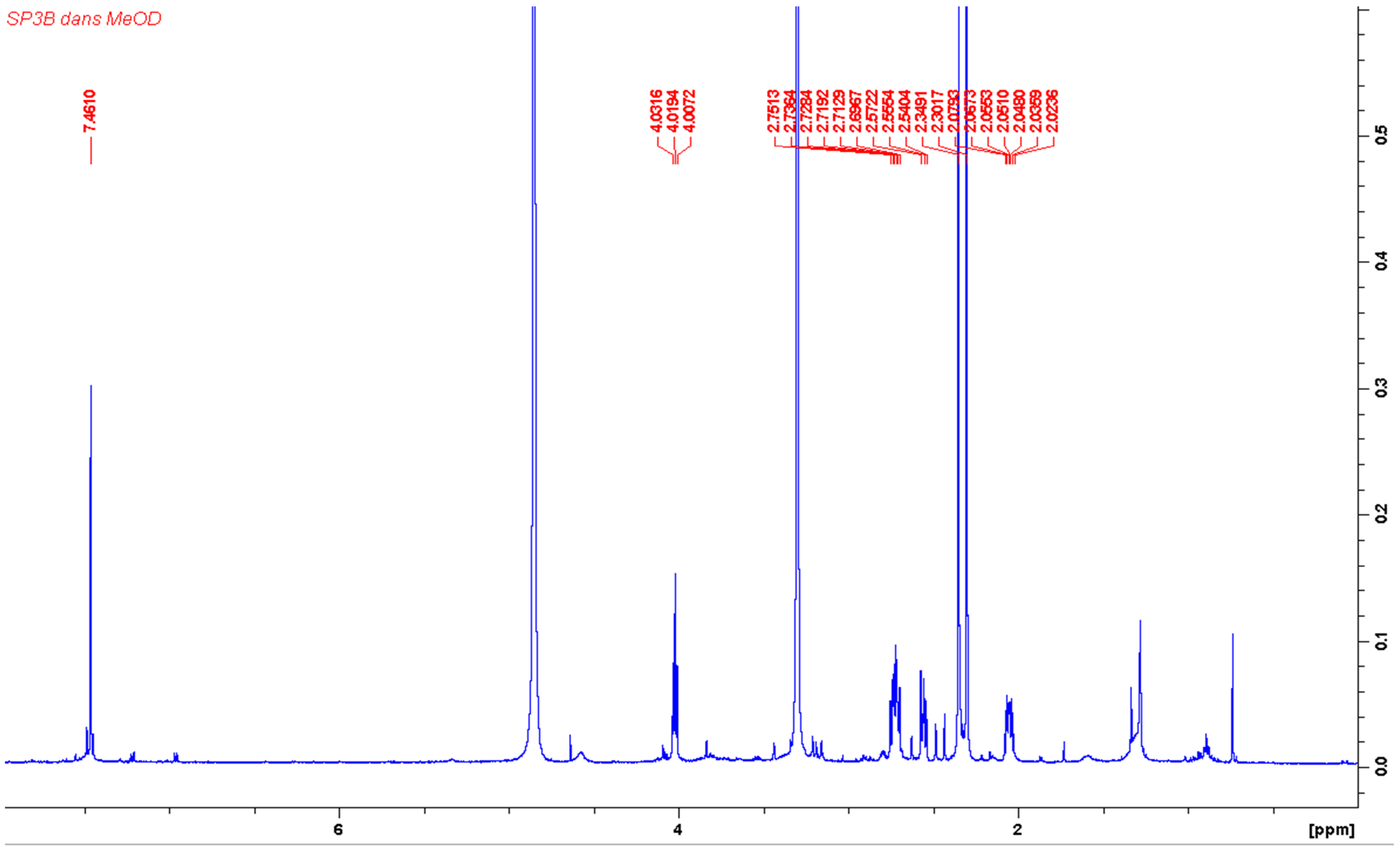

Figure S33. NMR ^{1}H in CD_3OD of compound **5**

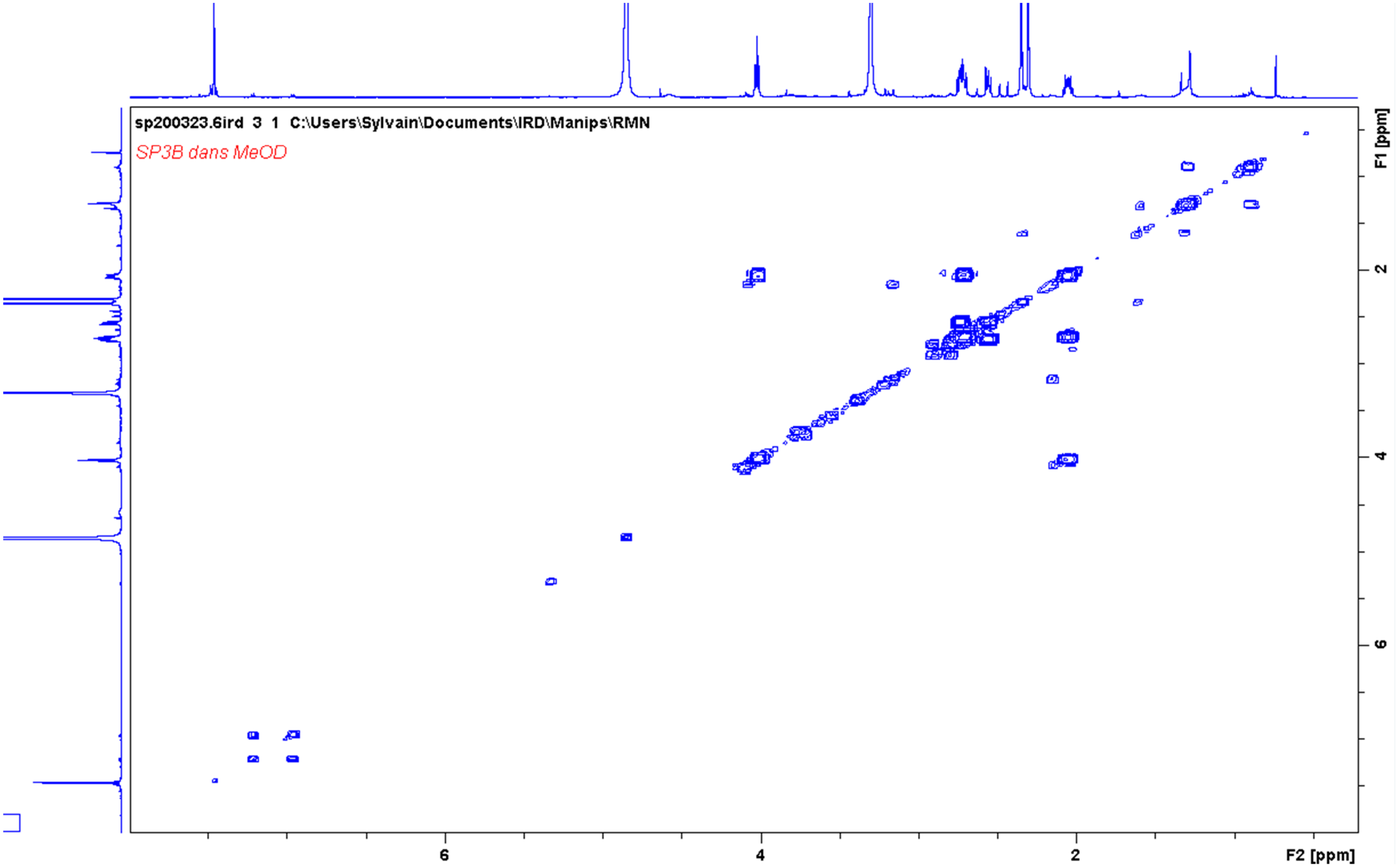

Figure S34. NMR COSY ^{1}H-^{1}H in CD_3OD of compound **5**

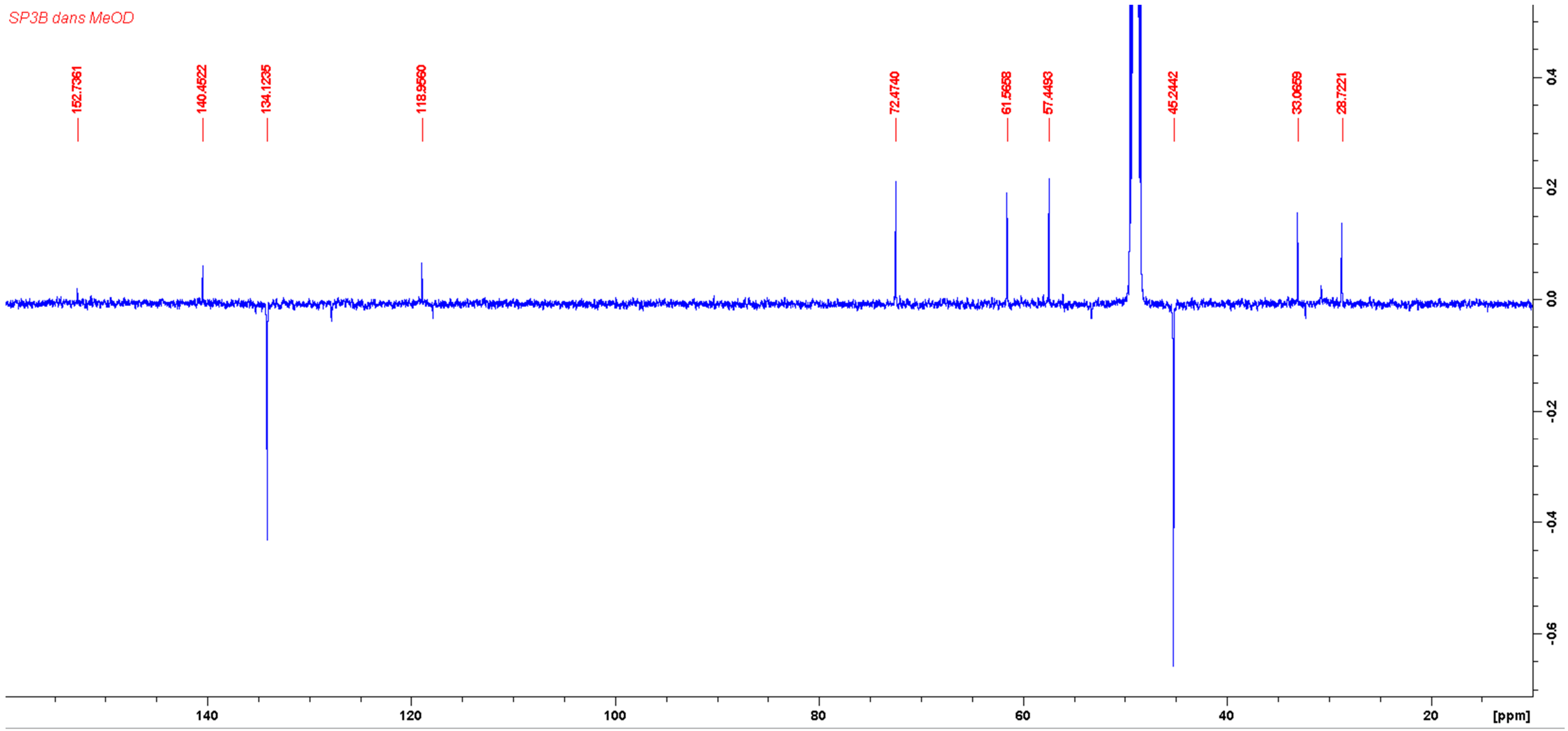

Figure S35. NMR JMOD ^{13}C in CD_3OD of compound **5**

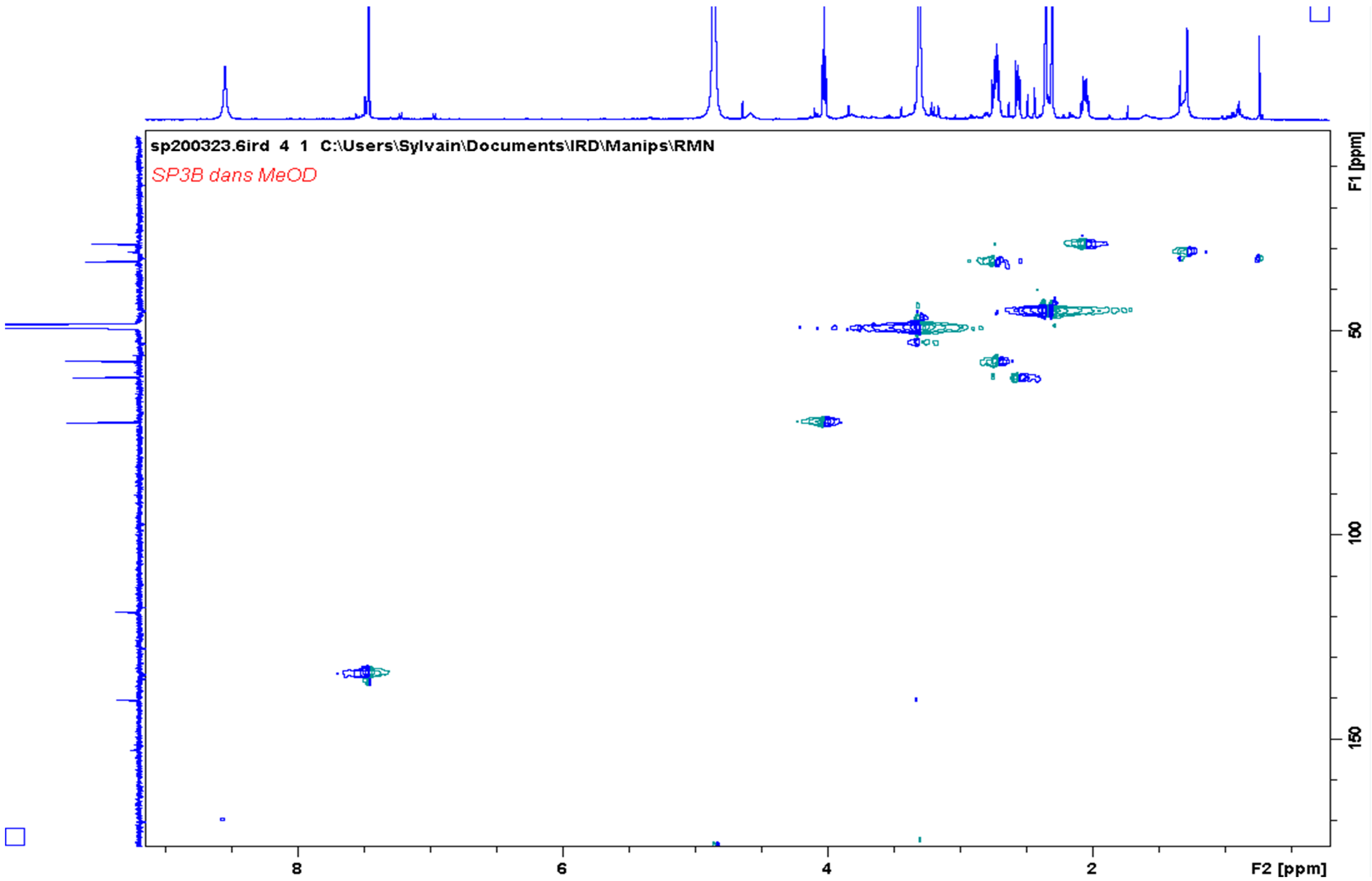

Figure S36. NMR HSQC ^{1}H-^{13}C in CD_3OD of compound **5**

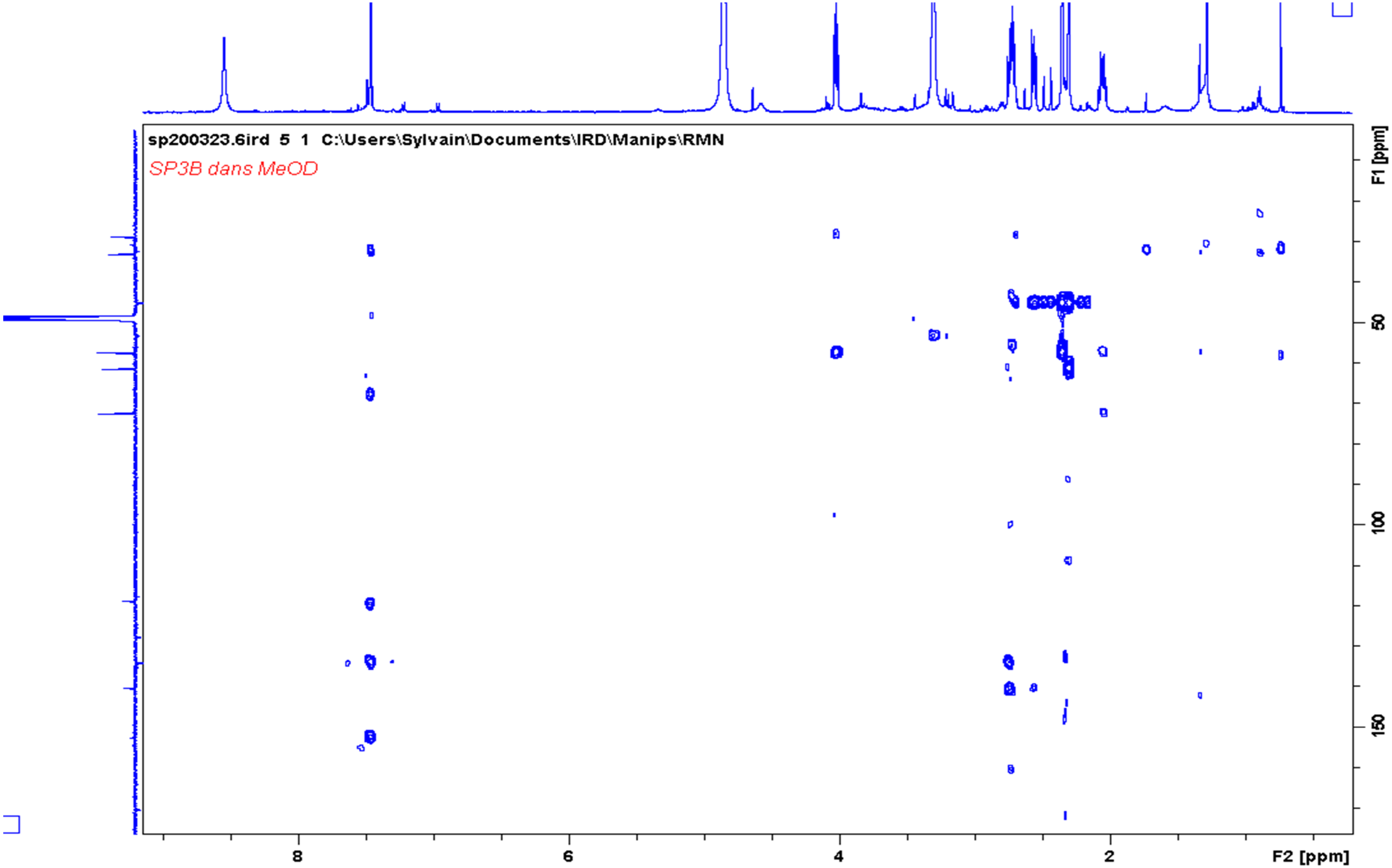

Figure S37. NMR HMBC ^1H-^{13}C in CD_3OD of compound **5**

Table S7. Comparison of the spectroscopic data of the compound **6** with the data from the literature concerning the pseudoceratinine B.

	Compound **6**		Pseudoceratinine B [61]	
MS data				
	HRESIMS data showed the $[M+H]^+$ ion as a cluster at *m/z* 502, 504, 506 in a 1:2:1 ratio, indicating the presence of two bromine atoms in the molecule. Analysis of the pic $[M+H]^+$ at *m/z* 504.0449 (504.0609 calc. for $C_{18}H_{28}N_5O_2Br_2$,) allowed us to propose the molecular formula of **8** as $C_{18}H_{27}N_5O_2Br_2$.		FABMS $[M+H]^+$ *m/z* 502, 504, 506.	
1D NMR data				
Position	in CD_3OD		in CD_3OD	
No	δ_C, type	δ_H mult, (*J* in Hz)	δ_C, type	δ_H mult, (*J* in Hz)
1	133.7		134.0	
2	133.5	7.55, s	132.5	7.55, s
3	118.0		118.9	
4	154.0		154.8	
5	118.0		118.9	
6	133.5	7.55, s	132.5	7.55, s
7	30.9	3.30, m	32.0	3.30, m
8	75.0	3.95, m	75.8	4.25, dd (12.0, 4.0)
9	164.2		166.2	
10	38.6	3.30, m	38.7	3.30, m
11	25.9	2.51, m	25.2	2.45, m
12	129.4		125.3	
13	110.0	6.35, s	110.5	6.42, s
14	149.5		148.5	
OMe	60.0	3.85, s	61.0	3.82, s
NMe_3	52.0	3.30, s	53.2	3.32, s

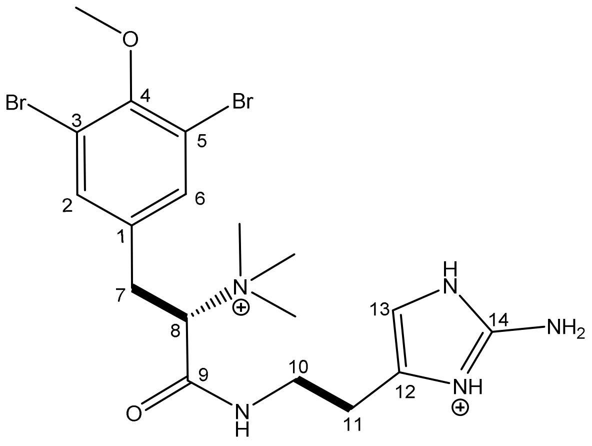

Figure S38. Formula for compound **6** corresponding to pseudoceratinine B

Compound **6** was isolated in minute quantities, with a few impurities. This identification was proposed after an in-depth study of the HMBC, COSY and HSQC spectra.

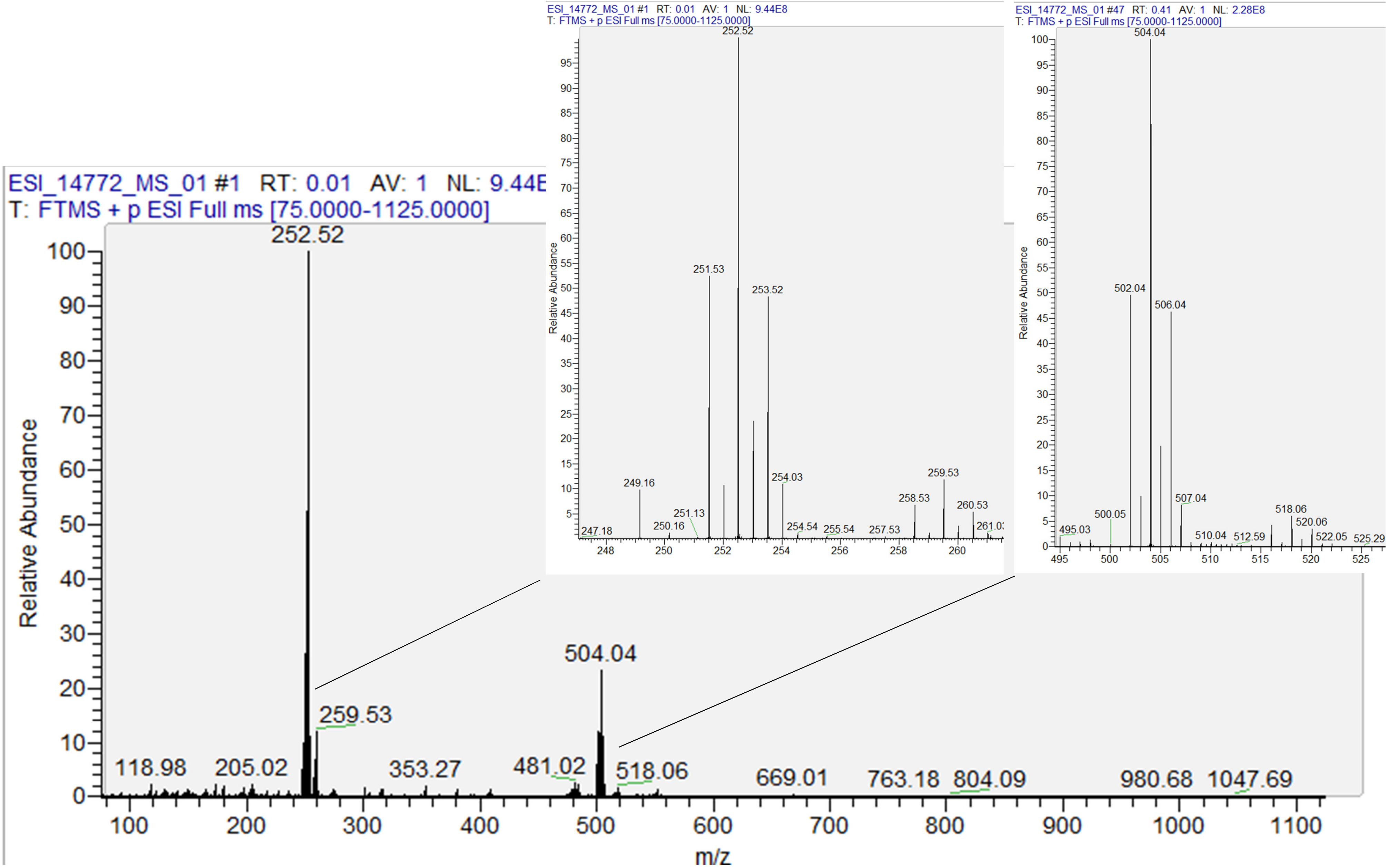

Figure S39. HRESIMS of compound **6**

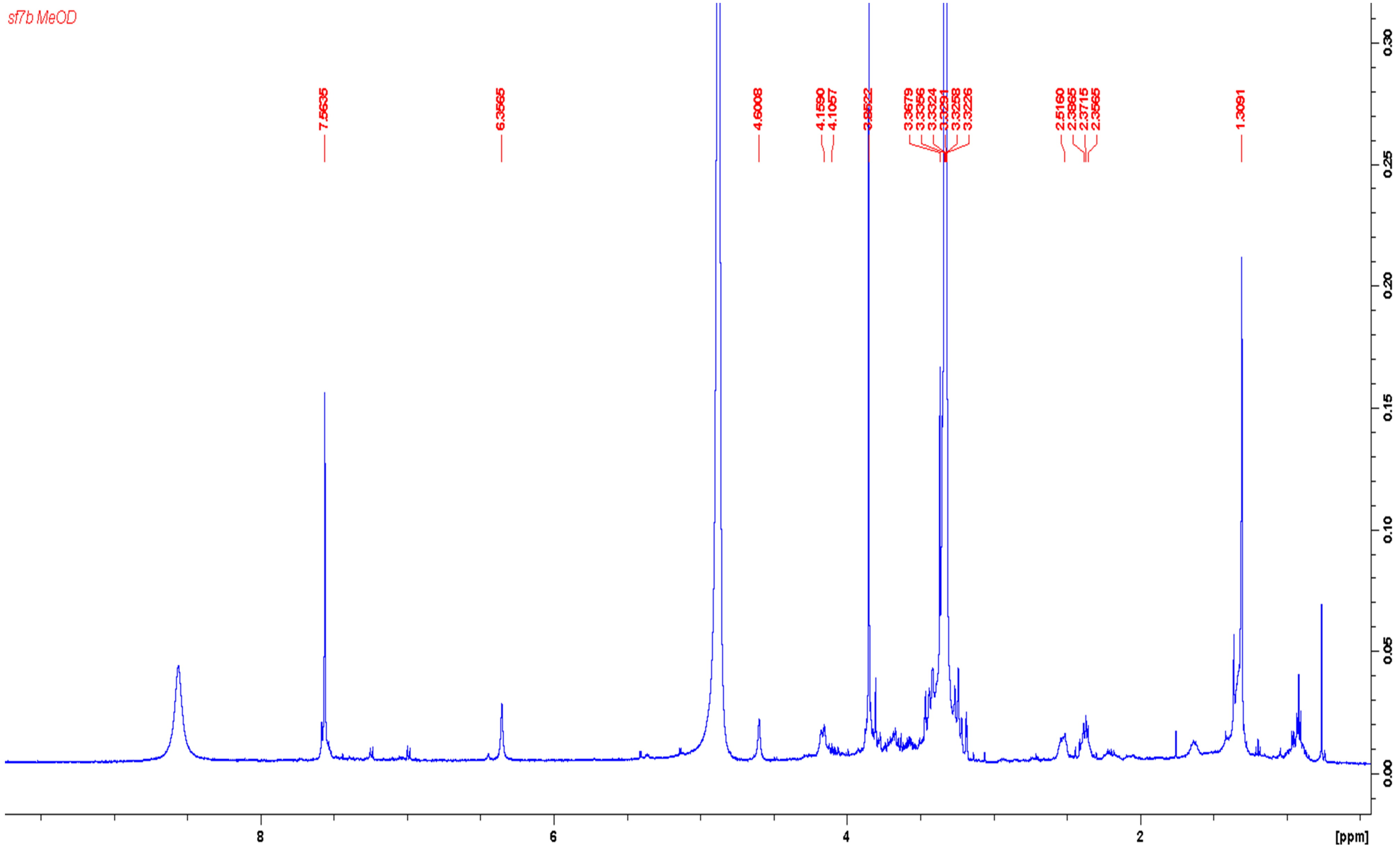

Figure S40. NMR ^{1}H in CD_3OD of compound **6**

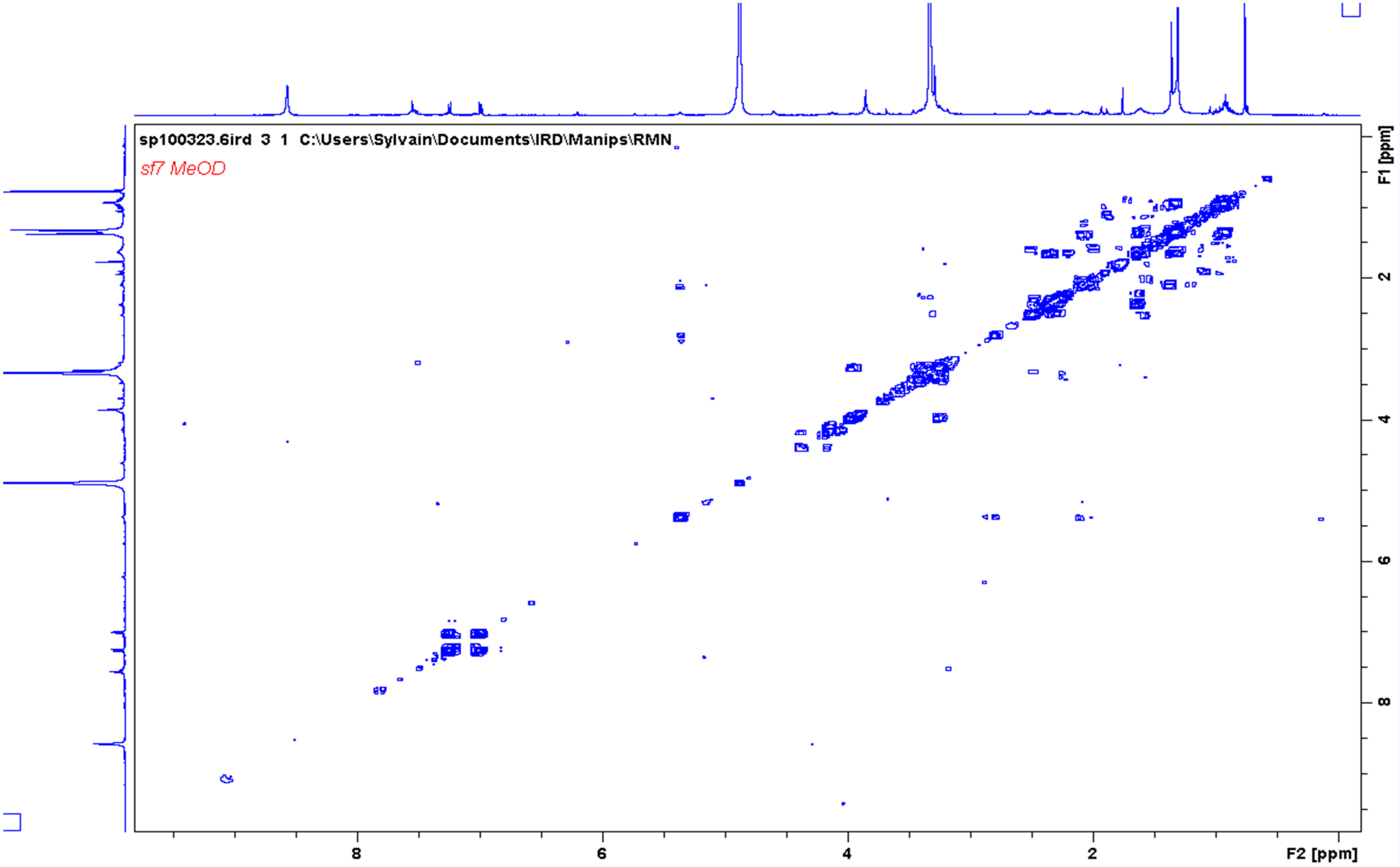

Figure S41. NMR COSY ^{1}H-^{1}H in CD_3OD of compound **6**

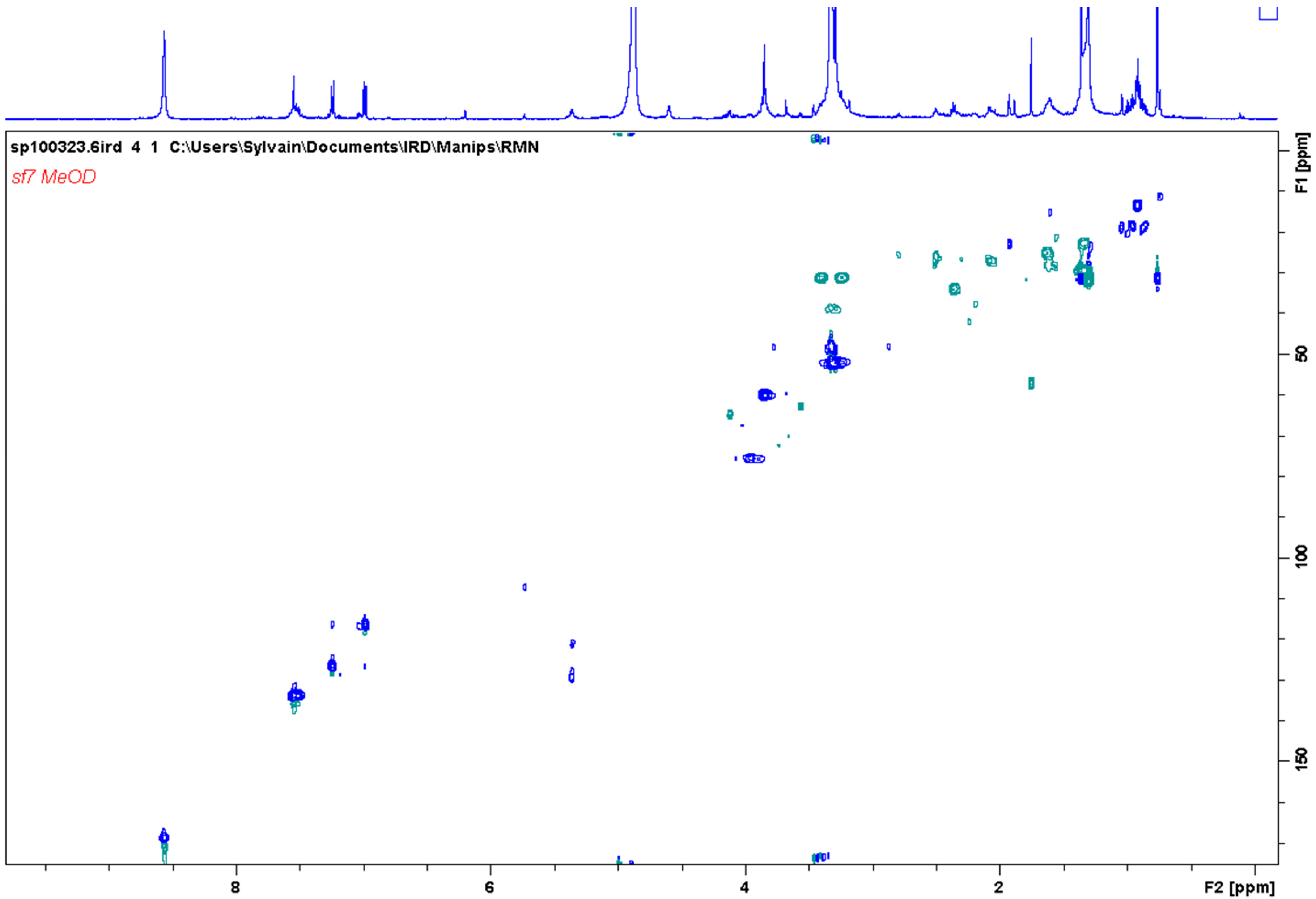

10

Figure S42. NMR HSQC ^{1}H-^{13}C in CD_3OD of compound **6** 11

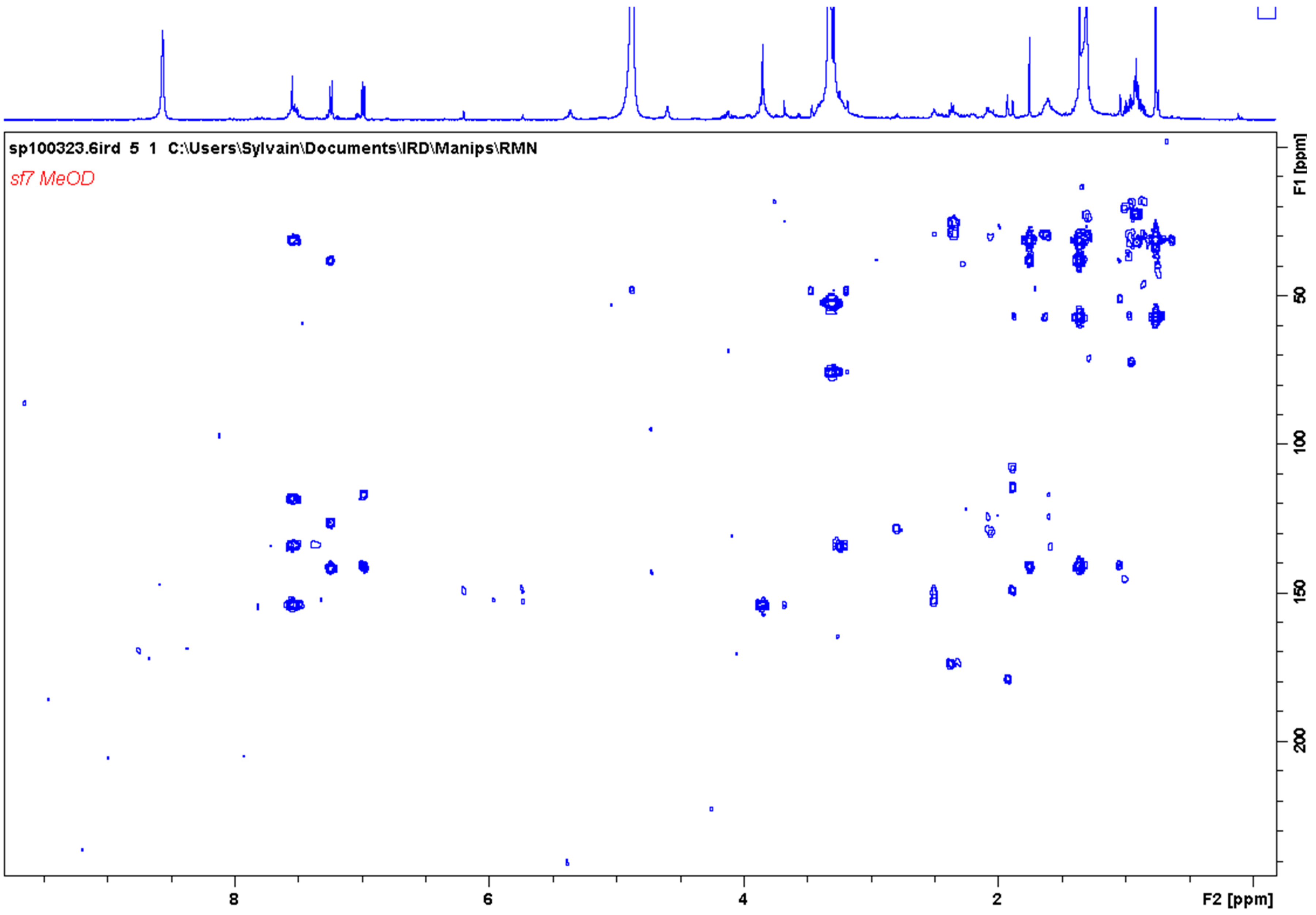

Figure S43. NMR HMBC ^{1}H-^{13}C in CD_3OD of compound **6**

Figure S1

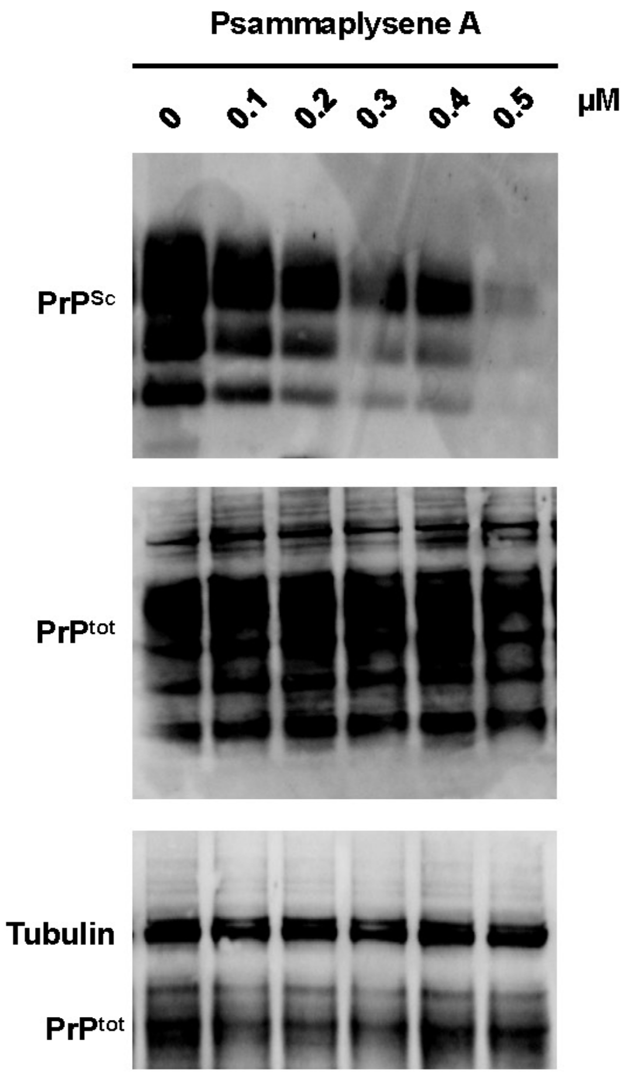

Figure S44. Original blots related to Figure S1

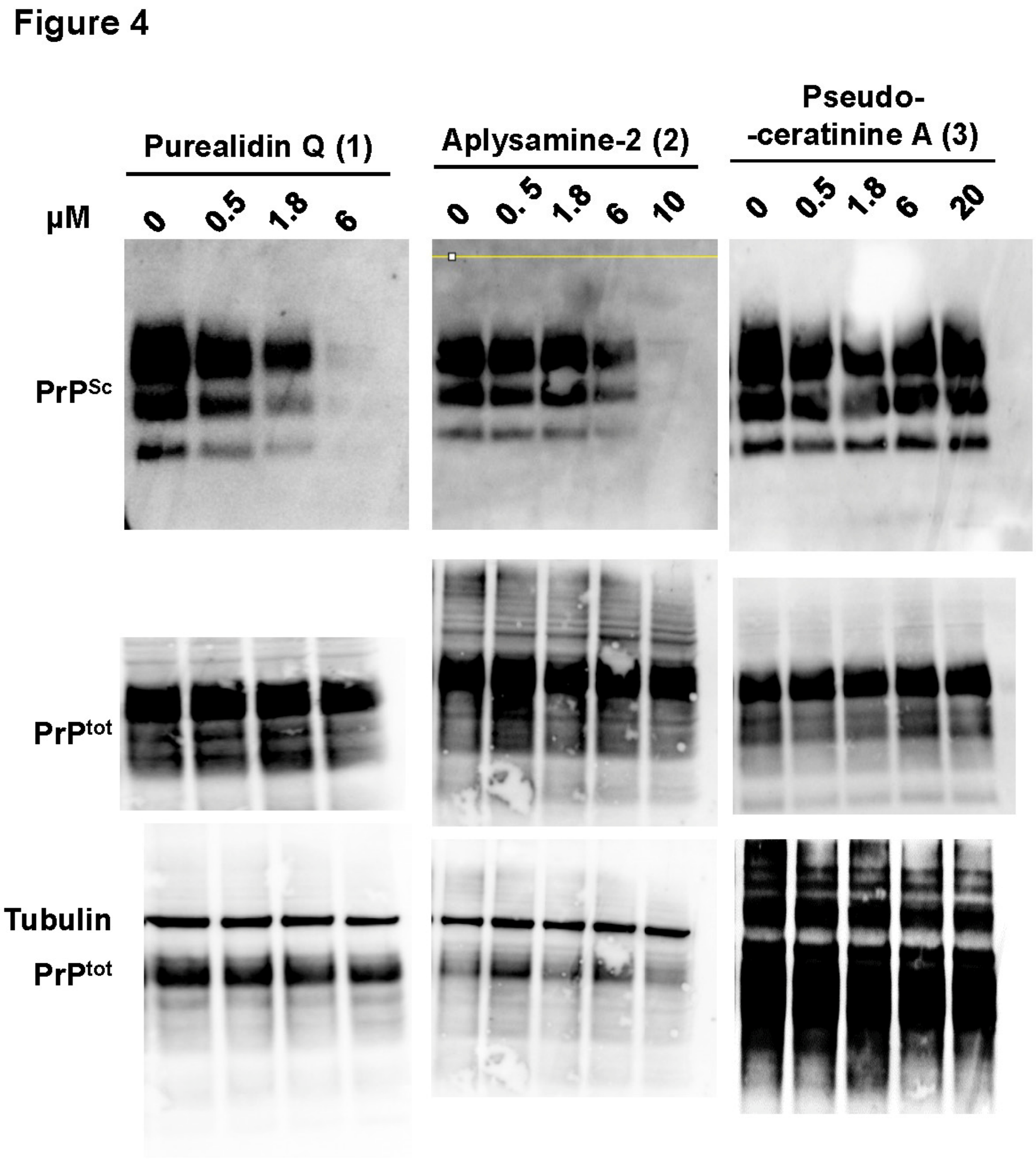

Figure S45. Original blots related to Figure 4 – part 1/2

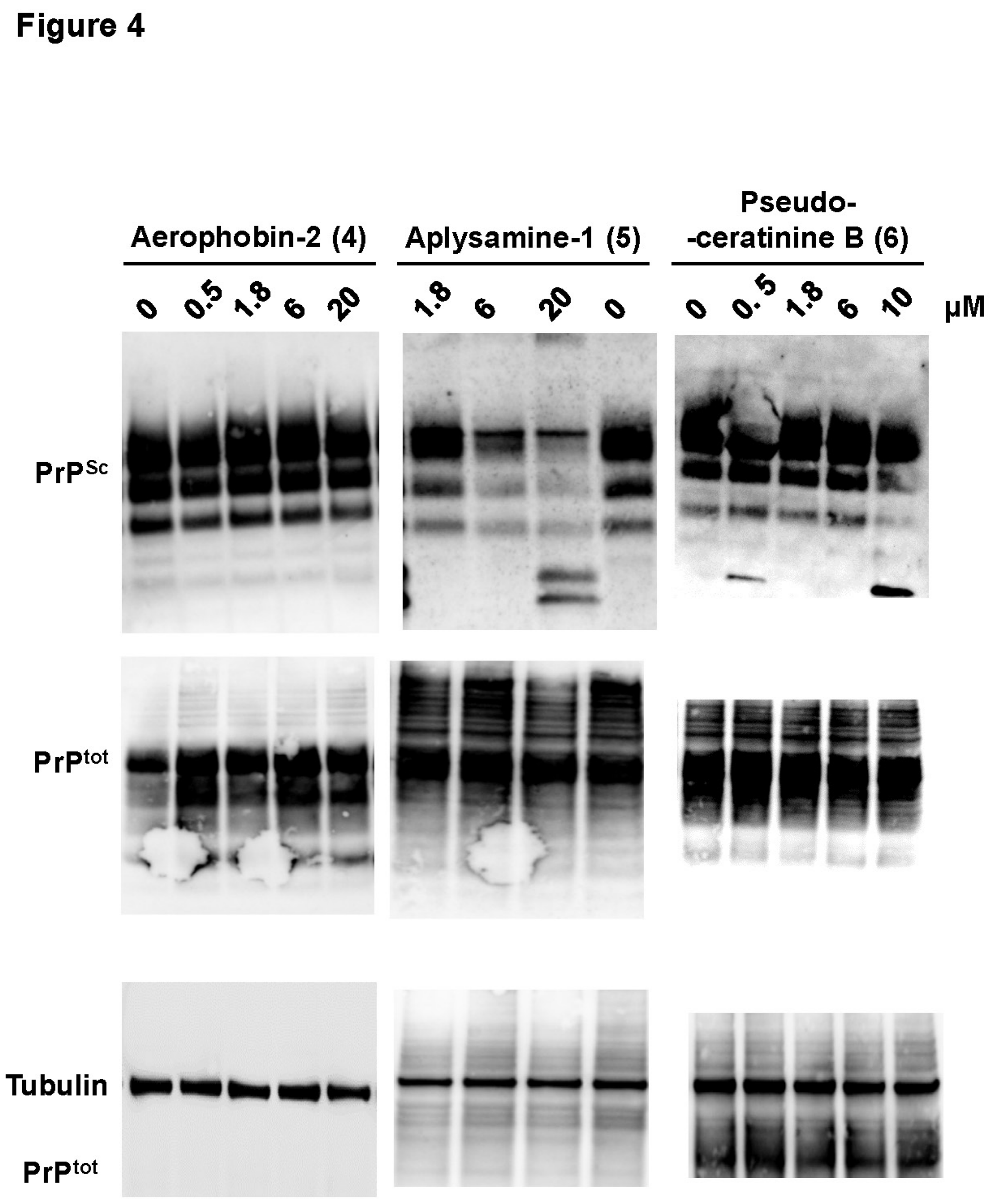

Figure S46. Original blots related to Figure 4 – part 2/2

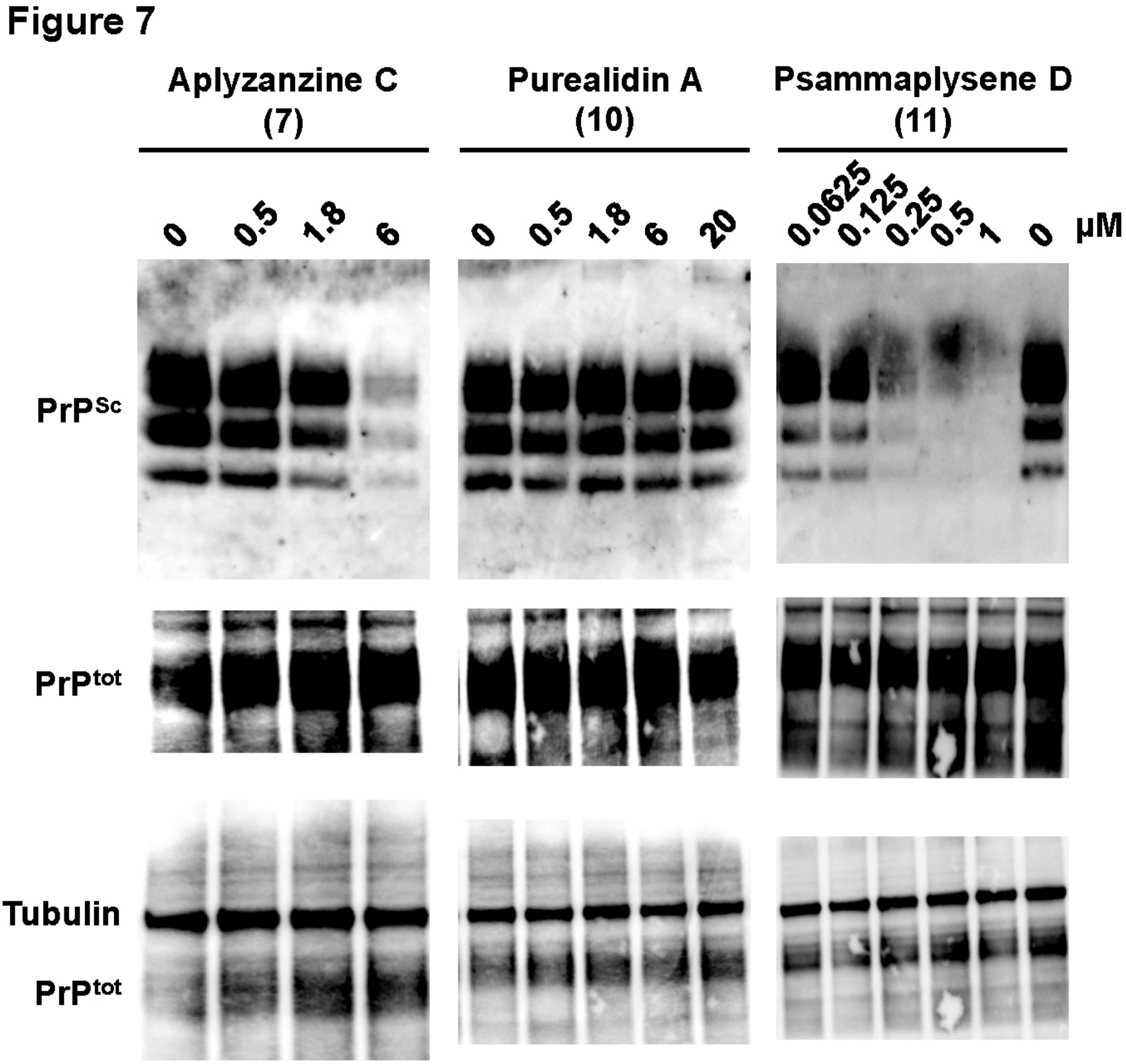

Figure S47. Original blots related to Figure 7 – part 1/2

Figure 7

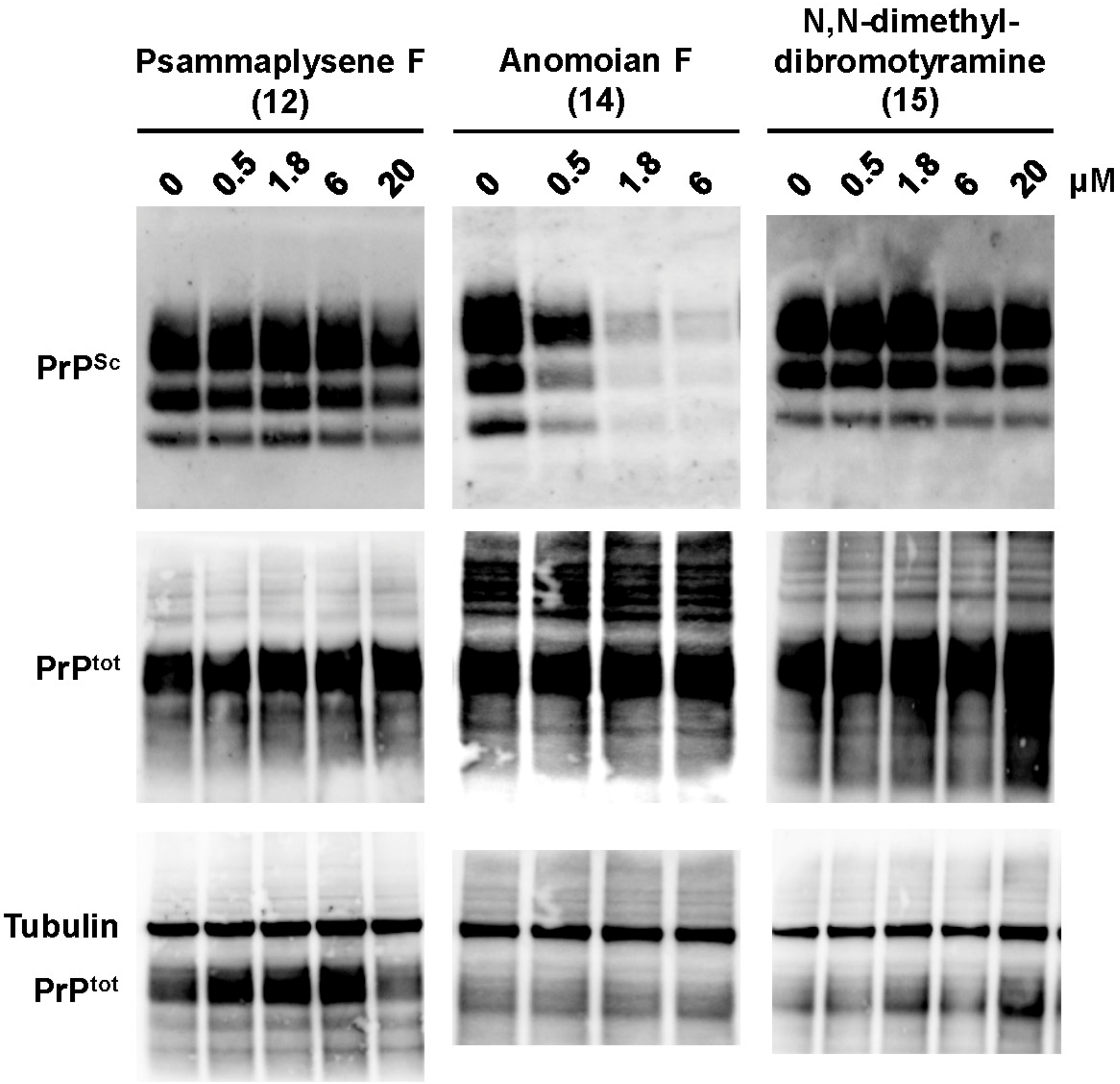

Figure S48. Original blots related to Figure 7 – part 2/2
